# The Use of Wearable Devices in Oncology Patients: A Systematic Review

**DOI:** 10.1093/oncolo/oyad305

**Published:** 2023-11-16

**Authors:** Ronald Chow, Hannah Drkulec, James H B Im, Jane Tsai, Abdulwadud Nafees, Swetlana Kumar, Tristan Hou, Rouhi Fazelzad, Natasha B Leighl, Monika Krzyzanowska, Philip Wong, Srinivas Raman

**Affiliations:** Princess Margaret Cancer Centre, University Health Network, Toronto, ON, Canada; Temerty Faculty of Medicine, University of Toronto, Toronto, ON, Canada; Institute of Biomedical Engineering, Faculty of Applied Sciences & Engineering, University of Toronto, Toronto, ON, Canada; Temerty Faculty of Medicine, University of Toronto, Toronto, ON, Canada; The Hospital for Sick Children, Toronto, ON, Canada; Princess Margaret Cancer Centre, University Health Network, Toronto, ON, Canada; Princess Margaret Cancer Centre, University Health Network, Toronto, ON, Canada; Princess Margaret Cancer Centre, University Health Network, Toronto, ON, Canada; Princess Margaret Cancer Centre, University Health Network, Toronto, ON, Canada; Princess Margaret Cancer Centre, University Health Network, Toronto, ON, Canada; Princess Margaret Cancer Centre, University Health Network, Toronto, ON, Canada; Temerty Faculty of Medicine, University of Toronto, Toronto, ON, Canada; Princess Margaret Cancer Centre, University Health Network, Toronto, ON, Canada; Temerty Faculty of Medicine, University of Toronto, Toronto, ON, Canada; Princess Margaret Cancer Centre, University Health Network, Toronto, ON, Canada; Temerty Faculty of Medicine, University of Toronto, Toronto, ON, Canada; Princess Margaret Cancer Centre, University Health Network, Toronto, ON, Canada; Temerty Faculty of Medicine, University of Toronto, Toronto, ON, Canada

**Keywords:** wearable devices, oncology, treatment monitoring, prognostication

## Abstract

**Introduction:**

The aim of this systematic review was to summarize the current literature on wearable technologies in oncology patients for the purpose of prognostication, treatment monitoring, and rehabilitation planning.

**Methods:**

A search was conducted in Medline ALL, Cochrane Central Register of Controlled Trials, Embase, Emcare, CINAHL, Scopus, and Web of Science, up until February 2022. Articles were included if they reported on consumer grade and/or non-commercial wearable devices in the setting of either prognostication, treatment monitoring or rehabilitation.

**Results:**

We found 199 studies reporting on 18 513 patients suitable for inclusion. One hundred and eleven studies used wearable device data primarily for the purposes of rehabilitation, 68 for treatment monitoring, and 20 for prognostication. The most commonly-reported brands of wearable devices were ActiGraph (71 studies; 36%), Fitbit (37 studies; 19%), Garmin (13 studies; 7%), and ActivPAL (11 studies; 6%). Daily minutes of physical activity were measured in 121 studies (61%), and daily step counts were measured in 93 studies (47%). Adherence was reported in 86 studies, and ranged from 40% to 100%; of these, 63 (74%) reported adherence in excess of 80%.

**Conclusion:**

Wearable devices may provide valuable data for the purposes of treatment monitoring, prognostication, and rehabilitation. Future studies should investigate live-time monitoring of collected data, which may facilitate directed interventions.

## Teaser

This article summarizes the current literature on wearable technologies in oncology patients for the purpose of prognostication, treatment monitoring, and rehabilitation planning.

**Implications for Practice**

Wearable devices may provide valuable data for the purposes of treatment monitoring, prognostication, and rehabilitation. Future studies should investigate live-time monitoring of collected data, which may facilitate directed interventions.

## Introduction

Over the past decade, there has been an increased interest in medicine and oncology for the adoption of wearable health technologies, such as smart watches, patches, and clothing that can track and record health vitals.^1-3^ It has been postulated that the data provided from wearable devices could provide additional information to the medical team about a patient’s health state, and facilitate better care.^4,5^

For patients with cancer, tracking biometric data could provide valuable insights to clinicians during various phases of treatment. For example, baseline activity metrics such as steps and heart rate can inform about a patient’s health state and be used for prognostication and treatment selection. Likewise, longitudinal analysis of health vitals can help to identify any concerning patterns related to adverse events, as well as monitor rehabilitation and exercise regimens.

To date, very few systematic reviews have reported on the application of wearable technologies in oncology patients. In a review of wearables in oncology trials by Beauchamp et al,^5^ 25 studies were included and notable heterogeneity of measured variables by wearable technologies was reported. In another review by Kos et al, 14 studies were included, and a weak to moderate association was observed between wearable technologies and performance status.^6^ However, no review has yet to report on the use of wearable technologies in patients with cancer, for the specific purposes of prognostication, treatment monitoring, and rehabilitation planning. As well, given the rapidly developing body of literature, a rigorous systematic review and overview of the literature can be valuable to understanding the current landscape of the literature, and if needed, recommend standardized research and reporting practices for future work.

The aim of this systematic review was to summarize the current literature on wearable technologies in oncology patients for the purpose of prognostication, treatment monitoring, and rehabilitation planning.

## Methods

### Search Strategy

In collaboration with an information specialist (RF), a comprehensive search was executed in Medline ALL (Medline and Epub Ahead of Print and In-Process and Other Non-Indexed Citations), Cochrane Central Register of Controlled Trials, Embase Classic + Embase, Emcare, all from the OvidSP platform; CINAHL from EBSCOhost; Scopus from Elsevier; and Web of Science from Clarivate Analytics. The literature searches were conducted from the inception of each database to February 2022, and there were no language restrictions. Each search strategy comprised a combination of controlled vocabulary terms and text words, adapting the database-specific search syntax. Where available, the search was limited to human studies, clinical trials, controlled clinical trials, randomized controlled trials, multicenter studies, and comparative studies. The randomized controlled trials filter by CADTH1^7^ was adapted with additional terms to ensure the study designs’ robustness (Supplementary Appendix 1).

### Screening and Eligibility

Following a calibration exercise of 20 articles, search results were screened via level 1 title and abstract screening independently and in-duplicate for each record by 2 review authors (R.C., T.H.), to identify studies that reported on wearable devices in oncology. Wearable devices were defined as any medical- or consumer-grade electronic devices that could be worn on the user’s body to measure physiologic or activity data. All solid and hematologic malignancies of any stage were included. Any discrepancies were resolved by discussion and consensus. If consensus was not achieved, a third and senior review author (S.R.) resolved the despite.

Relevant articles subsequently underwent level 2 full text screening, to review the articles and categorize them by their focus on intended use: prognostication, treatment monitoring, or rehabilitation. The prognostication category primarily included papers which use biometric data to correlate or predict for a clinical outcome. The treatment monitoring category primarily included studies in which patients received cancer therapy, and biometric data were used to characterize changes in clinical parameters or detect adverse events. The rehabilitation category primarily included patients who received cancer therapy, and biometric data were utilized to correlate with physical activity, quality of life, or other measures of well-being. Some papers met criteria for inclusion in multiple categories, and in this case, the studies were arbitrarily assigned a category that was felt to best match the above descriptions. Articles that reported solely on wearables for sleep (ie, actigraph only) were excluded. All level 2 screening was conducted by 2 of 4 review authors (R.C., H.D., A.N., S.K.) independently and in-duplicate for each record. Discrepancies were resolved by discussion and consensus; if consensus was not achieved, a third and senior review author (S.R.) resolved the despite.

### Data Extraction

For each included study, patient demographics and oncology treatment characteristics were noted. As well, details pertaining to wearable devices (brand, measured data, pattern of use, adherence, intervention vs. monitoring intent) and outcomes of the study were recorded. Each study was also appraised for study quality; randomized controlled trials were assessed using the Risk of Bias version 2 tool,^8^ and non-randomized studies using the Risk of Bias in Non-Randomised Studies of Interventions tool.^9^ Narrative synthesis was conducted to identify the common trends/patterns across the literature.

## Results

**Figure 1.**
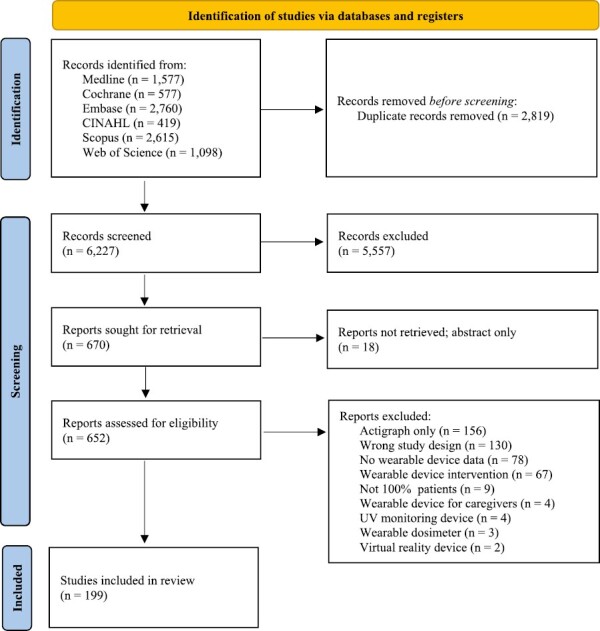
PRISMA flow diagram.

The search strategy identified 9046 articles. After duplicates were removed, 6227 records were screened. Ultimately, 199 studies^10-208^ reporting on 18 513 patients were included in this systematic review (Fig. 1). Two studies reported on different wearable device data about one patient population from Australia,^73,74^ another 2 from the US in the late 2000s^150,151^ and mid 2010s,^154,155^ and another 2 from Canada^183,184^; in total, 17 805 unique patients were reported across 195 unique datasets. There was a low risk of bias observed in the reported studies (>75%) (Supplementary Fig. S1).

Over half of studies (101; 51%) were conducted in the US. Eighteen were conducted in Canada, 18 in The Netherlands, 16 in Australia, 8 in the United Kingdom, 7 in Japan, 6 in Sweden, and 5 in Germany. Sample size ranged from 5 to 1447 patients (median = 46 patients). Sixty-eight studies (34%) reported on exclusively patients with breast cancer and 17 (9%) on exclusively patients with lung cancer. Forty studies (20%) reported on patients with any cancer diagnosis. Individual study characteristics are presented in Supplementary Table S1.

A description of each study’s wearable device, its usage, adherence, and conclusion of measured data by the wearable device is reported in Supplementary Table S2. One hundred and twelve studies used wearable device data primarily for the purposes of rehabilitation, 68 for treatment monitoring, and 19 for prognostication. Over three-quarters of studies (184; 92%) reported on a wearable device that functioned as a pedometer. Ten studies (5%) reported on a wearable device that functioned as both a pedometer and heart rate monitor. Four studies (2%) reported on a heart rate monitor, and one study (<1%) reported on both a pedometer and continuous glucose monitor. The most commonly-reported brands of wearable devices were ActiGraph (71 studies; 36%), Fitbit (37 studies; 19%), Garmin (13 studies; 7%), and ActivPAL (11 studies; 6%). Duration of wearable device use ranged from 1 week to over 1 year, with the majority of studies reporting activity monitoring for less than 1 month.

Daily minutes of physical activity were measured in 121 studies (61%) and daily step counts were measured in 93 studies (47%). Heart rate was measured in 12 studies (6%). One hundred and sixty-eight studies (84%) reported on the use of wearable devices during a single continuous period; 31 studies (16%) reported on the use of wearable devices for a short-time period at discrete timepoints. Twenty-eight studies (14%) investigated the use of wearable devices before treatment, 81 (41%) while patients were receiving treatment, and 140 (70%) after treatment. Only 6 studies (3%) involved live-monitoring of wearable device data; the majority of studies (193; 97%) collected the data for retrospective offline review. Adherence was reported in 86 studies and ranged from 40% to 100%; of these, 63 (73%) reported adherence in excess of 80%.

With regard to the various categories of applications, the majority of prognostic studies were conducted in the pretreatment phases, whereas most of the treatment monitoring and rehabilitation planning studies were conducted in the on-treatment and post-treatment phases. The average sample sizes in the prognostication (*n* = 87.9) and rehabilitation planning (*n* = 119.5) categories were larger than the treatment monitoring (*n* = 50.8) category. The average adherence rates in the 3 applications were very similar: prognostication (83%), treatment monitoring (88%), and rehabilitation planning (84%). The most common wearable device in all 3 applications was a pedometer.

Wearable device data were most assessed for correlation relative to physical activity, sedentary behavior, performance status, mood, and hospital outcomes of length of stay and hospitalization risk. Of the 111 articles reporting degree of significance between wearable device data and clinical outcome of interest, 87 studies reporting significant relationships using a type I error of 0.05.

## Discussion

To our knowledge, this is the first comprehensive systematic review reporting on the use of wearable technologies in oncology patients with applications categorized by prognostication, treatment monitoring, and rehabilitation planning. The results of thematic analysis in this review suggest that the use of wearable devices in oncology provides significant added value at each of the aforementioned treatment phases.

These added values include (1) providing objective, reliable, and relevant metrics; that can (2) inform the efficacy of various fitness/lifestyle interventions on increasing physical activity; which is (3) associated with various clinical outcomes of interest, which in sum can inform management in the different phases of the patient with cancer’s journey.

1. Wearable devices supplement cancer care and research with objective and reliable data on patient physical activity, and add value by providing clinically relevant metrics that are otherwise difficult to capture.

Across studies that examined the accuracy and reliability of wearable devices, results showed good convergence on the robustness of data derived from wearable technologies, especially when compared to patient- or clinician-reported data on physical activity or fitness.^21,40,45,147,173,194^ While medical-grade wearable devices such as ActiGraph provided more sensitive and accurate measurements, consumer-grade devices such as Fitbit provided sufficient accuracy to achieve its intended purposes of objectively measuring patient physical activity.^209^ Consumer-grade devices are therefore budget-friendly and viable alternatives that may be effectively utilized,^13,14^ particularly in less resource-rich regions. Moreover, wearable devices add value to cancer care by monitoring and quantifying the impact of cancer and cancer treatments on relevant metrics that are otherwise difficult to capture. Such methods of data capture are especially valuable when concerned with demographics such as children and the elderly who may struggle to accurately and consistently track their own physical activities.^147^In addition, wearable devices capture physical activity data in continuous, real-time, free-living settings without interrupting participants’ day-to-day.^11,17,18,21-23,25,40,44,45,51,62,63,66,76,83,85,86,89,90,92,104,108,111,112,119,122,126,128,129,136,139,142,143,147,157,158,161,163,166,172,173,176-178,181,188,192,194,208^ Of the studies that reported on the adherence of wearable devices, 43 (70%) of them cited high adherence at greater than 80%. Additionally, feasibility studies generally noted positive experience/satisfaction using wearable devices, citing ease of use, comfort, usefulness, and no interference with daily activity related to Fitbits, ActivPal, and Biovotion AG devices.^34,47,54,60,67,104,129,170,187,197^ However, one study by Finley et al noted considerable frustrations with its bulk/comfort and ease of use related to the Garmin Vivoactive HR device.^49^ While it is important for the adoption of wearable device to address such potential concerns, its generally high adherence and acceptability, in conjunction with the added benefits of objective, continuous data collection in free-living settings, provide strong support that wearable devices can be readily and effectively integrated into cancer care.

1. Wearable devices can play a key role in motivating patient physical activity throughout the treatment phases, by directly increasing physical activity or indirectly through informing the efficacy of various fitness/lifestyle interventions.

Considering the correlation between increased physical activity and improved patient outcomes (as discussed later), as well as observed good adherence and feasibility of wearable devices, wearable technology provides value in cancer care by increasing physical activity both directly and indirectly. While wearable devices were usually coupled with various fitness/lifestyle interventions across the studies included in this review, some studies suggest wearable devices alone were primary contributing factors to intervention efficacy.^28,47,48,170^ They suggest that the continuous monitoring itself enables patients to better manage and motivate their physical activity levels, directly contributing to increased physical activity. In addition, the adoption of wearable devices in oncology research indirectly supports increases in physical activity by informing the efficacy of various fitness/lifestyle interventions in increasing patient physical activity relative to control/comparison groups. This in turn helps to identify effective interventions for future adoption.

Wearable devices are also a relatively low-cost and accessible way for individuals to increase physical activity relative to traditional facility-based programs, in a manner that empowers and respects the individuals’ time and lifestyle.^13,15,54^ Furthermore, from the healthcare providers’ perspective, wearable devices may be a less resource-intensive way to bolster patient physical activity compared to follow up calls and visits.^28^ Nevertheless, there remain ongoing challenges that must be addressed when integrating the use of wearable devices in cancer research and patient care. For example, one must consider the limitation of wearables in tracking specific types of physical activity^196^ (eg, weight training, swimming) or physical activity of frail individuals^171^ (eg, walking very slowly), as step count or minutes of walking or running would not suffice. Additionally, in the few studies that demonstrated no significant effect of wearable devices and interventions on increasing physical activity, possible limitations include practical barriers to adherence (eg, technical difficulties,^190^ poorer physical functioning,^46^ financial barriers^160^); psychological barriers to adherence (eg, preference for personal vs. automated support, emotional/social/cognitive challenges)^145,185^; and intervention design (eg, duration or intensity of intervention, insufficient statistical power).^65,70,80,103,150,151^ Such findings can be very informative for rehabilitation planning by highlighting the importance of accessible, personal, multimodal intervention programs tailored to the needs of individuals,^10,13,14,28,55^ particularly in the context of patients with cancer and survivors relative to the healthy population; as well as ongoing support, possibly through peer- or community-support programs.

1. Physical activity is associated with various important clinical outcomes, which can inform patient prognostication.

The studies included in this review inform prognostication by examining physical activity levels and its correlates to identify potential patient profiles and their association with various clinical outcomes. These outcomes include physical well-being measures such as anthropometric measures,^31,45,51,63,64,70,82,106,109,113,124,179^ performance status,^26,39,56,62,67,85,120,128^ motor function,^71,92,208^ functional recovery,^17,117^ bone health,^162^ and symptoms or pain,^12,27,38,39,41,51,56,63,99,126,163^ quality of life^13-16,18,24,25,29,32,34,37,39,41,46,47,53,60,67,69,75,80,87,93,101,111,113,114,116,124,126,131,132,146,149,153,181,183-185,199,206^; psychological well-being measures such as sleep,^20,27,31,61,68,91,98,111,146,153,154,156,177^ fatigue,^30,37,38,44,54,57,61,62,64,69,71,79,83,87,91,111,112,132,152,166,192^ cognition,^19,42,43,61,80,106,111^ and mood/anxiety/depression^38,41,54,63,93,121^; physiological and biochemical measures such as glycemic control,^33^ blood pressure,^30^ C-reactive protein levels,^88,101,159^ interleukin-6,^88,101^ metabolic and inflammatory markers,^65^ and respiration^23,30,89,90,200^; and notably hospital outcomes such as hospitalization risk,^62,123^ hospital length of stay,^10,21,107,141,148,207^ readmission,^129^ complications or adverse events,^21,35,148,172^ and disability-free or overall survival.^62,97,123^

It is worthy to note that 117 of the 199 studies investigated the association between wearable device data and physical and/or psychosocial outcomes such as health-related quality of life, and these studies also included relatively larger cohorts of patients. This suggests stronger evidence supporting the value of integrating wearable device data to investigate physical and/or psychosocial outcomes, as well as its feasibility and validity as a part of any oncological rehabilitation program, ranging from children and adolescents^60^ to the elderly.^114^

Conversely, far fewer studies examined the association between wearable device data with physiological/biochemical factors (6 studies) as well as hospital outcomes (13 studies). As well, of the 13 studies that examined hospital outcomes, only 2 involved wearable device interventions (while the rest only used wearable devices to monitor). In turn, future studies should seek to better understand how interventions involving the use of wearable devices can directly impact physiological/biochemical patient markers or hospital outcomes.

While studying physical and/or psychosocial outcomes such as quality of life has obvious implications for understanding patient well-being, the investigation of how wearable device data is associated with hospital outcomes can provide greater insight on the potential cost savings for healthcare providers by integrating wearable devices across different phases of patients’ treatment journey.

Despite the potential clinical utility of wearable device data suggested by these studies, there are further opportunities to demonstrate and harness its full potential through its integration with machine learning. For instance, only one study in this review conducted by Cos et al^35^ applied machine learning to clinical and physical activity data, which outperformed standard tools for predicting patient outcomes. Given the ability for machine learning to interpret data rapidly and repetitively without exhausting human resources, it can be used as a screening tool to identify events where clinical intervention may be needed.

Also, given that the majority of studies in the review are single arm studies and post hoc analyses of clinical trials, future studies should focus on comparative outcomes, ideally through randomized trial designs, to prospectively demonstrate the value added from wearable devices.

Future studies should also investigate potential economic advantages to deploying wearable technologies. Based on the results of this study, we believe that such cost-savings can occur in 3 ways. As mentioned earlier, wearable devices can increase physical activity levels at a relatively lower cost to individuals than facility- or membership-based interventions.^54,56,160^ It can also decrease the cost for healthcare providers by serving as remote monitoring and rehabilitation, thereby lowering the cost of resources related to more rigorous clinical testing (eg, administering the 6-minute walk test)^21^ and travel/clinical visits.^56^ Finally, wearable devices can be used to support increased physical activity. Particularly in the context of patients with cancer who underwent surgery, early mobilization and physical activity has been shown to be important for decreasing hospital length of stay and, in turn, healthcare costs.^10,141,207^ According to Hall et al, hospital inpatient and hospice stays, community care, outpatient appointments, out-of-hours service, and travel costs were the main costs related to cancer rehabilitation.^70^ Evidently, the adoption of wearable devices can address most if not all of these cost drivers.

In addition to measuring physical activity, as done by most of the studies in this review, there is potential to collect other biometric data from patients. Only 4 studies in our review used wearables to primarily measure other parameters: heart rate and gait. van der Stam et al^189^ found that HealthDot is an acceptable wearable device that provides good quality heart rate data. Shih et al^168^ found that continuously measured heart-rate variability correlated strongly with fatigue scores in patients with lung cancer. Schink et al^164^ and Zahiri et al^208^ used wearables to investigate the associations between gait and functional declines due to cancer or cancer treatment. While the majority of trials focused on physical activity and step count, there are opportunities to collect other sources of active and passive sensor data from wearable devices as well. Other metrics that could be collected could include glucose and vital signs using commercially available technology. These tools hold great potential in increasing the frequency of data collection and identifying novel insights that are not otherwise captured through current follow-up schedules. However, the accuracy of the data must be clearly established before clinical deployment. For example, Lee et al showed that different wearable devices had error rates between 9.3% and 23.5% in the measurement of daily energy expenditure.^210^ Such findings further highlight the importance of assessing data accuracy and adequate uses given limited accuracy or robustness, especially if used to replace some element of clinical assessment.

Based on the results of this review as well as aforementioned future uses of wearable devices, it is clear that wearable devices can be used to collect large amounts of clinically relevant patient data that is not routinely captured with current workflows. Naturally, this could present significant security and privacy challenges that will need to be overcome. Depending on where the data are stored, this information may be vulnerable to attacks and data leakage. Furthermore, wearable technologies themselves may be subject to faults that cancreate possibilities for data and privacy leaks.^211^ Ultimately, once these challenges related to infrastructure, legal or regulatory issues, and reimbursement are addressed, it remains to be seen how wearable devices and wearable data can be integrated and harnessed to optimize patient care and research in oncology.

In conclusion, our review of 199 studies indicate that there is a wealth of evidence to support that the use of wearable devices is feasible in oncology patients with high-adherence rates and can provide valuable data across all phases of a patient’s journey. The most well-researched applications of wearable device data are its associations with physical and psychosocial outcomes such as quality of life, which have implications for the potential benefits to patient or survivor well-being by integrating wearable devices into oncological rehabilitation programs. Clinical applications for these devices worth further investigating include physiological/biochemical measures and hospital outcome measures and their association with wearable device data. Finally, wearable device data can be coupled with machine learning for promising opportunities in characterizing patient profiles for early prognostication.

## Supplementary Material

Supplementary material is available at *The Oncologist* online.

oyad305_suppl_Supplementary_Material

## Data Availability

This is a systematic review of published literature. All data are publicly available.
