# Supplementary material for: The Use of Wearable Devices in Oncology Patients: A Systematic Review
Source: Oncologist. 2023 Nov 16;29(4):e419–30. doi: 10.1093/oncolo/oyad305 (PMC10994271; doi:10.1093/oncolo/oyad305)
Supplement: oyad305_suppl_Supplementary_Material [file oyad305_suppl_supplementary_material.docx]

**Supplemental Figure 1.** Study Quality of Included Studies **1.1** Randomized Controlled Trials **1.2** Observational Studies

**S1.1**


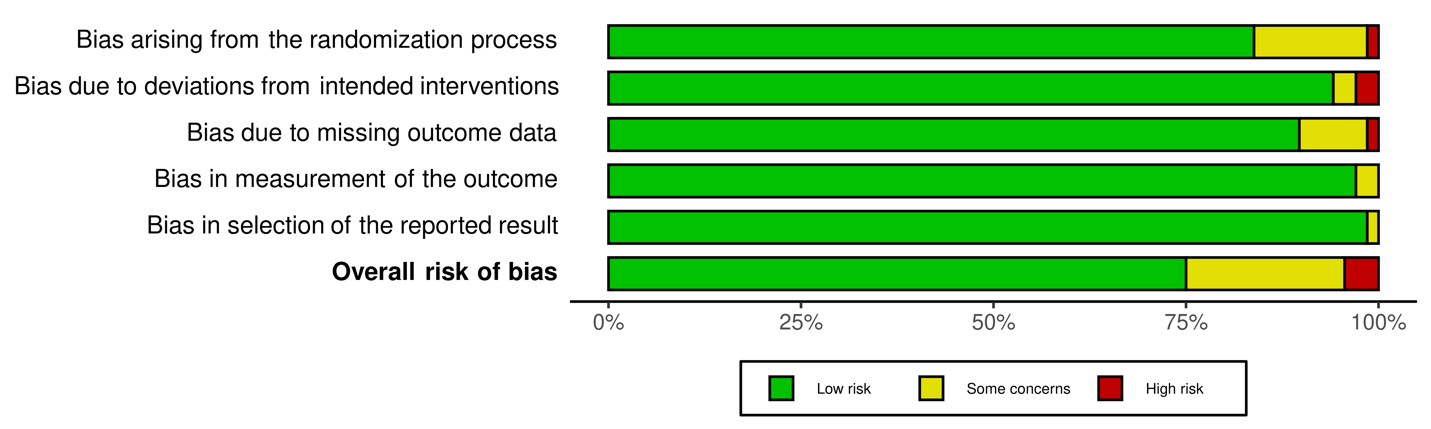


**S1.2**


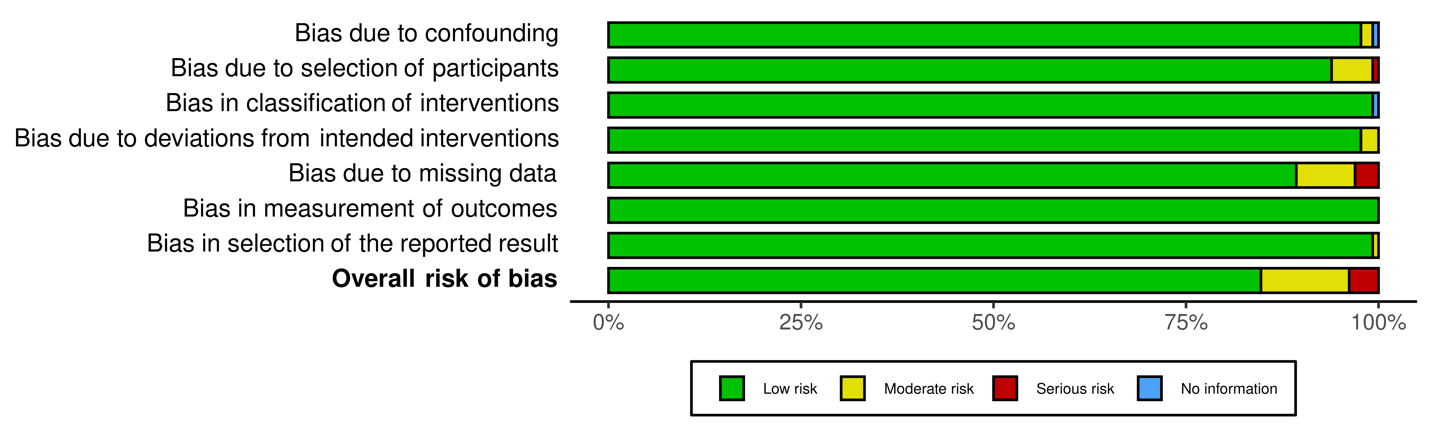


**Table S1.** Study Characteristics

| **Study** | **Country** | **N** | **Age** | | **% Female** | **Cancer Diagnosis** | **Treatment** |
| --- | --- | --- | --- | --- | --- | --- | --- |
|  |  |  | **Mean ± SD** | **Median (IQR)** |  |  |  |
| Au 2019 ^10^ | Canada | 38 | 59.9 ± 7.1 |  | 0.0% | Prostate | Radical Prostatectomy |
| Awick 2017 ^11^ | United States of America | 370 | 56.2 ± 9.4 |  | 100.0% | Breast | Any |
| Backman 2014 ^12^ | Sweden | 77 | Not reported |  | Not reported | Breast, Colorectal | Adjuvant Chemotherapy |
| Bade 2018 ^13^ | United States of America | 30 | 66.0 ± 7.8 |  | 33.3% | Lung | Any |
| Bade 2018 ^14^ | United States of America | 37 | 66.4 ± 8.6 |  | 29.7% | Lung | Any |
| Bade 2021 ^15^ | United States of America | 40 | 64.9 ± 8.7 |  | 75.0% | Lung | Any |
| Ballinger 2021 ^16^ | United States of America | 57 | 59.2 ± 9.4 |  | 100.0% | Breast | Any |
| Barkley 2019 ^17^ | United States of America | 22 |  | 68 (63-73) | 31.8% | Abdominal | Surgery |
| Bekkering 2012 ^18^ | The Netherlands | 44 | 14.9 ± 4.8 |  | 38.6% | Malignant Bone Tumor | Surgery |
| Bender 2021 ^19^ | United States of America | 73 | 63.7 ± 5.3 |  | 100.0% | Breast | Aromatase Inhibitor Therapy |
| Bernard 2016 ^20^ | Canada | 66 | 52.4 ± 9.6 |  | 100.0% | Breast | Radiation |
| Bille 2021 ^21^ | United Kingdom | 78 | Not reported |  | 47.4% | Lung | Surgery |
| Boyle 2017 ^22^ | Canada | 156 | 62.2 ± 12.9 |  | 48.7% | Non-Hodgkin Lymphoma | Any |
| Braam 2016 ^23^ | The Netherlands | 60 | 13.8 |  | 41.7% | Any | Any |
| Breedveld-Peters 2018 ^24^ | The Netherlands | 145 | 70.0 ± 8.7 |  | 37.2% | Colorectal | Any |
| Broderick 2014 ^25^ | United Kingdom | 24 | 50.9 ± 12.8 |  | 100.0% | Breast | Chemotherapy |
| Broderick 2019 ^26^ | United States of America | 42 | 48.2 ± 12.4 |  | 50.0% | Any | Chemotherapy |
| Bulls 2019 ^27^ | United States of America | 138 | 61.0 ± 10.3 |  | 100.0% | Gynecologic | Chemotherapy |
| Cadmus-Bertram 2019 ^28^ | United States of America | 50 | 54.4 ± 11.2 |  | 96.0% | Breast, Colorectal | Any |
| Caperchione 2019 ^29^ | Canada | 87 | Not reported |  | 100.0% | Breast | Any |
| Carter 2016 ^30^ | United States of America | 152 | 55 ± 8 |  | 100.0% | Breast | Any |
| Champ 2018 ^31^ | United States of America | 10 | 68.0 ± 9.5 |  | 100.0% | Breast | Surgery, Then Radiation |
| Chan 2022 ^32^ | United States of America | 41 | 55.0 ± 11.3 |  | 58.5% | Colorectal | Any |
| Chestnut 2020 ^33^ | United States of America | 5 | Not reported |  | 0.0% | Bladder | Any |
| Chow 2021 ^34^ | United States of America | 41 |  | 45.1 | 48.8% | Leukemia, Lymphoma | Hematopoietic Cell Transplantation |
| Cos 2021 ^35^ | United States of America | 48 | 63.2 |  | 60.4% | Pancreatic | Surgery |
| Dennett 2018 ^36^ | Australia | 46 | 59 ± 12 |  | 63.0% | Any | Chemotherapy |
| Devine 2020 ^37^ | United States of America | 49 | 18.5 ± 3.7 |  | 49.0% | Any | Chemotherapy |
| Dore 2022 ^38^ | Canada | 199 | 55.0 ± 11.0 |  | 100.0% | Breast | Any |
| Dorion 2017 ^39^ | Canada | 12 |  | 62 (58-66.5) | 41.7% | Any | Radiation |
| Douma 2018 ^40^ | The Netherlands | 72 | 63.0 ± 11.5 |  | 37.5% | Any | Any |
| Edbrooke 2019 ^41^ | Australia | 80 | 63.1 ± 12.3 |  | 43.8% | Lung | Any |
| Ehlers 2017 ^42^ | United States of America | 299 | 57.5 ± 9.5 |  | 100.0% | Breast | Chemotherapy, Radiation, Chemotherapy And Radiation, Or Hormonal Therapy |
| Ehlers 2018 ^43^ | United States of America | 286 | 57.8 ± 9.5 |  | 100.0% | Breast | Chemotherapy, Radiation, Chemotherapy And Radiation, Or Hormonal Therapy |
| Erickson 2021 ^44^ | United States of America | 47 | 32.3 ± 5.2 |  | 79.0% | Any | Chemotherapy |
| Fazzino 2017 ^45^ | United States of America | 142 | 58.6 ± 8.0 |  | 100.0% | Breast | Any |
| Fazzino 2018 ^46^ | United States of America | 176 | 58.2 ± 8.1 |  | 100.0% | Breast | Any |
| Ferrante 2020 ^47^ | United States of America | 35 | 61.5 ± 8.8 |  | 100.0% | Breast | Any |
| Ferrante 2022 ^48^ | United States of America | 44 | Not reported |  | 100.0% | Breast | Any |
| Finley 2020 ^49^ | United States of America | 30 | 67.3 |  | 57.1% | Lung | Surgery |
| Finley 2021 ^50^ | United States of America | 30 | 67.5 ± 10.6 |  | 56.7% | Lung | Surgery |
| Fouladiun 2007 ^51^ | Sweden | 39 | 71 ± 2 |  | 28.2% | Any | Any |
| Frensham 2020 ^52^ | Australia | 91 | 65.6 ± 9.3 |  | 48.4% | Any | Any |
| Gaskin 2016 ^53^ | Australia | 147 | 65.6 ± 8.5 |  | 0.0% | Prostate | Any |
| Gell 2017 ^54^ | United States of America | 24 | 57.9 ± 10.4 |  | 83.3% | Any | Any |
| Gell 2019 ^55^ | United States of America | 66 | 61.4 ± 9.0 |  | 83.3% | Any | None |
| Ghods 2021 ^56^ | United States of America | 27 |  | 58.0 (37.0-83.0) | 37.0% | Gastrointestinal | Radiation Or Chemotherapy |
| Gielissen 2012 ^57^ | The Netherlands | 83 | 47.4 ± 9.0 |  | 47.0% | Any | None |
| Gilchrist 2019 ^58^ | Canada | 199 | 55.0 ± 10.9 |  | 100.0% | Breast | None |
| Gomersall 2019 ^59^ | Australia | 36 | 64.8 ± 9.6 |  | 36.1% | Any | Surgery |
| Gotte 2018 ^60^ | Germany | 40 | 14.9 ± 3.8 |  | 45.0% | Any | Not reported |
| Gregoire 2020 ^61^ | Belgium | 95 | 53.9 ± 11.9 |  | 100.0% | Any | None |
| Gresham 2018 ^62^ | Unites States of America | 37 |  | 62 | 46.1 | Any | Any |
| Gresham 2021 ^63^ | United States of America | 31 | 67.1 ± 10.9 |  | 61.3 | Pancreatic | Not reported |
| Guest 2013 ^64^ | United States of America | 42 | 53.9 ± 9.1 |  | 100.0% | Breast | Chemotherapy |
| Guinan 2013 ^65^ | United Kingdom | 26 | 48.1 ± 8.8 |  | 100.0% | Breast | Any |
| Gundle 2017 ^66^ | United States of America | 25 | 16.0 ± 3.3 |  | 56.0% | Lower Extremity Osseous Malignancy | Surgery |
| Gupta 2018 ^67^ | United States of America | 24 | 54 ± 12.5 |  | 67.0% | Any | Chemotherapy |
| Gururaj 2021 ^68^ | India | 27 | 54.7 ± 10.3 |  | 14.8% | Head And Neck | Chemoradiotherapy |
| Hacker 2018 ^69^ | United States of America | 10 | 55.1 ± 9.3 |  | 30.0% | Any | Hematopoietic Stem Cell Transplantation |
| Hall 2021 ^70^ | Edinburgh | 45 | Not reported |  | 42.0% | Gastrointestinal, Thoracic, Breast, Urological/Gynecological, Myeloma, Head And Neck, Endocrine | Hormonal, Bisphosphonate And Steriods |
| Hamari 2019 ^71^ | Finland | 36 | 7.8 |  | 27.8% | Leukemia, Lymphoma | Vincristine |
| Hardcastle 2020 ^72^ | Australia | 28 | 65.0 ± 7.0 |  | 58.6% | Colorectal, Endometrial | Any |
| Hardcastle 2021 ^73^ | Australia | 68 | 64.1 ± 7.9 |  | 50.0% | Colorectal, Gynecologic | Any |
| Hardcastle 2021 ^74^ | Australia | 68 | 64.1 ± 7.9 |  | 50.0% | Colorectal, Gynecologic | Any |
| Hartman 2017 ^75^ | United States of America | 134 | 62.6 ± 6.6 |  | 100.0% | Breast | Surgery |
| Hartman 2018 ^76^ | United States of America | 42 | 57.9 ± 11.3 |  | 100.0% | Breast | Any |
| Hirschey 2018 ^77^ | United States of America | 60 | 58 ± 11 |  | 100.0% | Breast | Any |
| Hooke 2016 ^78^ | United States of America | 16 | 8.7 ± 3.1 |  | 73.3% | Leukemia | Maintenance Chemotherapy |
| Hooke 2019 ^79^ | United States of America | 57 | 12.0 ± 3.6 |  | 36.7% | Any | Not reported |
| Howell 2018 ^80^ | United States of America | 78 |  | 12.7 (11.0-15.0) | 55.1% | Any | Not reported |
| Irwin 2008 ^81^ | United States of America | 75 | 55.8 ± 8.6 |  | 100.0% | Breast | None |
| James 2015 ^82^ | Australia | 133 | 57.0 ± 12.0 |  | 77.4% | Any | None |
| Janssen 2021 ^83^ | The Netherlands | 330 | 56.0 ± 13.0 |  | 58.0% | Leukemia | Tyrosine Kinase Inhibitor |
| Javaheri 2015 ^84^ | Canada | 21 |  | 56.0 (38.0-78.0) | 85.7% | Breast, Head And Neck | Radiation |
| Jeffery 2017 ^85^ | Australia | 46 | 68.5 ± 7.9 |  | 28.3% | Malignant Pleural Effusion | Any |
| Jeffery 2022 ^86^ | Australia | 18 | 68.9 ± 7.1 |  | 11.1% | Mesothelioma | Any |
| Johnson 2021 ^87^ | United States of America | 50 | 33.6 ± 4.9 |  | 55.1% | Any | Any |
| Jones 2013 ^88^ | United States of America | 75 | 55.9 ± 8.7 |  | 100.0% | Breast | Any |
| Jonsson 2019 ^89^ | Sweden | 94 | 69 ± 8 |  | 52.1% | Lung | Surgery |
| Jonsson 2019 ^90^ | Sweden | 107 | 68.6 ± 7.8 |  | 56.0% | Lung | Surgery |
| Keadle 2021 ^91^ | United States of America | 51 | 36.9 ± 5.7 |  | 86.3% | Any | Any |
| Kong 2020 ^92^ | South Korea | 614 | 61.2 ± 9.0 |  | Not reported | Lung | Surgery |
| Koontz 2021 ^93^ | United States of America | 30 |  | 55 | 70.0% | Any | Any |
| Leach 2019 ^94^ | United States of America | 27 | 51.8 ± 8.6 |  | 100.0% | Breast | Surgery |
| Leach 2020 ^95^ | United States of America | 20 | 59.1 ± 8.4 |  | 50.0% | Colorectal | None |
| Long 2018 ^96^ | Australia | 13 |  | 19 | 53.8% | Cancer With Cerebral Insult | Any |
| Loprinzi 2019 ^97^ | United States of America | 515 | 67.7 |  | 56.1% | Any | Any |
| Loughney 2017 ^98^ | United Kingdom | 33 | 66 |  | 30.3% | Rectal | Chemoradiotherapy |
| Low 2021 ^99^ | United States of America | 44 | 65.7 |  | 40.9% | Pancreatic | Surgery |
| Lowe 2015 ^100^ | Canada | 31 | Not reported |  | Not reported | Brain Metastases | Radiotherapy |
| Lozano-Lozano 2019 ^101^ | Spain | 73 | 51.4 ± 8.6 |  | 100.0% | Breast | Chemotherapy |
| Lynch 2019 ^102^ | Australia | 83 | 61.6 ± 6.4 |  | 100.0% | Breast | Any |
| Maddocks 2009 ^103^ | United Kingdom | 16 | 60 ± 7 |  | 43.8% | Lung | Any |
| Maddocks 2010 ^104^ | United Kingdom | 60 | 68 ± 9 |  | 33.3% | Any | Any |
| Maeda 2016 ^105^ | Japan | 19 | 73.9 ± 7.7 |  | 15.8% | Lung | Surgery |
| Marinac 2015 ^106^ | United States of America | 136 | 62.6 ± 6.6 |  | 100.0% | Breast | Any |
| Martin 2020 ^107^ | Switzerland and France | 50 | 58.7 ± 18.2 |  | 32.0% | Colorectal | Surgery |
| Matsui 2021 ^108^ | Japan | 20 | 65.2 ± 10.0 |  | 10.0% | Esophageal | Surgery |
| Matthews 2007 ^109^ | United States of America | 36 | Not reported |  | 100.0% | Breast | Chemotherapy |
| McNeil 2019 ^110^ | Canada | 45 | 59 ± 9 |  | 100.0% | Breast | Any |
| Minton 2012 ^111^ | United Kingdom | 114 | 57.6 |  | 100.0% | Breast | Any |
| Miyaji 2020 ^112^ | Japan | 30 | 58.6 ± 10.6 |  | 30.0% | Any | Chemotherapy |
| Modesitt 2021 ^113^ | United States of America | 99 | 59.9 ± 9.4 |  | 100.0% | Breast, Gynecologic | Any |
| Mouri 2018 ^114^ | Japan | 30 |  | 75 | 33.3% | Lung, Pancreatic | Chemotherapy |
| Muller 2014 ^115^ | Germany | 21 | 13.9 ± 2.7 |  | 57.1% | Bone | Any |
| Muller 2016 ^116^ | Germany | 150 | 10.7 ± 4.3 |  | 48.7% | Any | Any |
| Mylius 2021 ^117^ | The Netherlands | 38 |  | 66.0 (58.3-74.8) | 42.1% | Hepato-Pancreato-Biliary | Surgery |
| Naito 2019 ^118^ | Japan | 30 |  | 75 | 33.3% | Lung, Pancreatic | Chemotherapy |
| Nelson 2021 ^119^ | United States of America | 32 | 49.6 ± 10.7 |  | 100.0% | Breast | Chemotherapy |
| Nilanon 2020 ^120^ | United States of America | 41 |  | 48.0 (24.0-72.0) | 56.1% | Not reported | Chemotherapy |
| Nyrop 2018 ^121^ | United States of America | 100 | 48.3 ± 9.4 |  | 100.0% | Breast | Chemotherapy |
| Ohri 2017 ^122^ | United States of America | 38 |  | 64 (33-82) | 39.5% | Head And Neck, Lung, Gastrointestinal | Fractionated Radiotherapy With Concurrent Chemotherapy |
| Ohri 2019 ^123^ | United States of America | 50 |  | 66 (38-90) | 40.0% | Lung | Chemoradiation |
| Ormel 2021 ^124^ | The Netherlands | 141 |  | 61.0 (34.0-74.0) | 100.0% | Breast | Endocrine Therapy |
| Park 2019 ^125^ | South Korea | 339 | 50.4 ± 9.5 |  | 100.0% | Breast | None |
| Park 2020 ^126^ | South Korea | 21 |  | 66.5 (61.0-71.8) | 0.0% | Prostate | Not reported |
| Parker 2019 ^127^ | United States of America | 50 | 66.0 ± 8.0 |  | 48.0% | Pancreatic | Systemic Chemotherapy, Chemoradiation Therapy |
| Paul 2020 ^128^ | United States of America | 46 |  | 66.0 (38.0-90.0) | 41.3% | Lung | Radiation Therapy |
| Pavic 2020 ^129^ | Switzerland | 31 |  | 64.0 (53.0-71.0) | 29.0% | Any | None |
| Perkins 2009 ^130^ | United States of America | 20 | Not reported |  | 100.0% | Endometrial | None |
| Phillips 2014 ^131^ | United States of America | 370 | 56.2 ± 9.4 |  | 100.0% | Breast | None |
| Phillips 2015 ^132^ | United States of America | 358 | 56.5 (9.0) |  | 100.0% | Breast | None |
| Phillips 2022 ^133^ | United States of America | 269 | 52.5 ± 9.9 |  | 100.0% | Breast | None |
| Pinto 2015 ^134^ | United States of America | 76 | 55.6 ± 9.6 |  | 100.0% | Breast | Chemotherapy, Radiation, Hormone Treatment |
| Pinto 2017 ^135^ | United States of America | 76 | 55.6 ± 9.6 |  | 100.0% | Breast | Chemotherapy, Radiation, Hormone Treatment |
| Pinto 2020 ^136^ | United States of America | 22 | 51.5 ± 8.4 |  | 100.0% | Breast | Chemotherapy, Radiation |
| Pinto 2021 ^137^ | United States of America | 20 | 71.6 ± 4.0 |  | 95.0% | Any | None |
| Pinto 2021 ^138^ | United States of America | 161 | 57.3 ± 10.9 |  | 100.0% | Breast | Radiation, Chemotherapy, Hormone Therapy |
| Piringer 2020 ^139^ | Austria | 25 | 62.4 ± 10.9 |  | 40.0% | Colorectal | Regorafenib |
| Pope 2018 ^140^ | United States of America | 30 | 57.0 ± 12.0 |  | 100.0% | Breast | None |
| Porserud 2019 ^141^ | Sweden | 133 | 68.1 ± 12.3 |  | 49.6% | Colorectal, Urinary Bladder, Ovarian | Any |
| Prinsen 2013 ^142^ | The Netherlands | 37 | 49.3 ± 9.8 |  | 51.4% | Malignant, Solid Tumor, Non-Hodgkin's Lymphoma | Any |
| Prinsen 2013 ^143^ | The Netherlands | 40 | 48.8 ± 10.4 |  | 50.0% | Malignant, Solid Tumor, Non-Hodgkin's Lymphoma | Any |
| Quintiliani 2016 ^144^ | United States of America | 10 | 58.6 ± 6.1 |  | 100.0% | Breast | Any |
| Rahimy 2021 ^145^ | United States of America | 46 |  | 61.0 (30.0-72.0) | 100.0% | Endometrial | None |
| Rastogi 2020 ^146^ | United States of America | 50 | 54.4 ± 11.2 |  | 96.0% | Colorectal, Breast | Chemotherapy And/Or Radiation |
| Rehorst-Kleinlugtenbelt 2019 ^147^ | The Netherlands | 25 |  | 8.2 (3.1-17.0) | 36.0% | Any | Not reported |
| Richards 2020 ^148^ | New Zealand | 85 |  | 76.0 (72.0-81.0) | 50.6% | Colorectal | Elective Colrorectal Surgery |
| Robertson 2019 ^149^ | United States of America | 74 | Not reported |  | Not reported | Endometrial | None |
| Rogers 2009 ^150^ | United States of America | 41 | 53.0 ± 9.0 |  | 100.0% | Breast | None |
| Rogers 2009 ^151^ | United States of America | 41 | 53.0 ± 9.0 |  | 100.0% | Breast | None |
| Rogers 2014 ^152^ | United States of America | 44 | 56.2 ± 7.7 |  | 100.0% | Breast | None |
| Rogers 2015 ^153^ | United States of America | 42 | 56.2 ± 7.7 |  | 100.0% | Breast | None |
| Rogers 2015 ^154^ | United States of America | 222 | 54.4 ± 8.5 |  | 100.0% | Breast | None |
| Rogers 2017 ^155^ | United States of America | 222 | 54.4 ± 8.5 |  | 100.0% | Breast | None |
| Roveda 2017 ^156^ | Italy | 42 | 55.2 ± 6.8 |  | 100.0% | Breast | None |
| Sabiston 2014 ^157^ | Canada | 177 | 54.9 ± 11.1 |  | 100.0% | Breast | None |
| Sabiston 2017 ^158^ | Canada | 187 | 55.0 ± 10.9 |  | 100.0% | Breast | None |
| Sabiston 2018 ^159^ | Canada | 138 | 55.3 ± 11.1 |  | 100.0% | Breast | None |
| Sabiston 2019 ^160^ | Canada | 30 | 55.7 ± 8.9 |  | 100.0% | Breast | None |
| Sada 2021 ^161^ | United States of America | 28 | 65.8 ± 9.8 |  | 32.1% | Not reported | None |
| Saito 2020 ^162^ | Japan | 53 | 67.4 ± 8.4 |  | 100.0% | Breast | Adjuvant Aromatase Inhibitors |
| Sande 2014 ^163^ | United Kingdom | 60 |  | 63.5 (38.0-88.0) | 58.3% | Bone Metastases | Radiation Therapy |
| Schink 2020 ^164^ | Germany | 41 | 60.0 ± 13.0 |  | 43.9% | Any | Not reported |
| Schrier 2021 ^165^ | United States of America | 24 |  | 63.0 (37.0-79.0) | 100.0% | Ovarian | None |
| Servaes 2002 ^166^ | The Netherlands | 114 | 45.0 ± 6.3 |  | 100.0% | Breast | None |
| Shah 2021 ^167^ | United States of America | 40 | 61.6 ± 10.6 |  | 27.5% | Gastrointestinal | Chemoradiation |
| Shih 2021 ^168^ | Taiwan | 12 | 65.3 ± 6.2 |  | 41.7% | Lung | Chemotherapy |
| Short 2015 ^169^ | Australia | 330 | 55.0 |  | 100.0% | Breast | None |
| Singh 2020 ^170^ | Australia | 52 | 51.2 ± 9.1 |  | 100.0% | Breast | Not reported |
| Skipworth 2011 ^171^ | Norway, Germany, Switzerland | 45 | 64.8 ± 12.5 |  | 51.1% | Aerodigestive, Urogenital, Breast | Not reported |
| Slade 2021 ^172^ | United States of America | 20 |  | 65.0 (61.0-74.0) | 10.0% | Bladder | Radical Cystectomy |
| Smith 2019 ^173^ | United States of America | 310 | 61.1 ± 6.9 |  | 0.0% | Prostate | Radical Prostatectomy |
| Stacey 2017 ^174^ | Australia | 29 | 56.4 ± 12.2 |  | 86.2% | Any | None |
| Strother 2021 ^175^ | United States of America | 33 |  | 65.0 (60.0-72.0) | 9.1% | Bladder | Radical Cystectomy |
| Sweegers 2019 ^176^ | Australia, Canada, The Netherlands, United States of America | 1447 | 59.3 ± 11.4 |  | 78.4% | Breast, Testicular, Haematological, Colorectal, Gynaecological, Lung | None, Surgery, Chemotherapy, Radiotherapy |
| Thuman 2019 ^177^ | United States of America | 8 | 54.4 |  | 100.0% | Breast | Masectomy, Implant-Based Reconstruction, Autologous Reconstruction |
| Timmerman 2018 ^178^ | The Netherlands | 23 | 59.0 ± 10.0 |  | 56.5% | Lung | Lung Resection |
| Tonorezos 2013 ^179^ | United States of America | 117 | 24.3 ± 4.9 |  | 55.6% | Leukemia | None |
| Trinh 2018 ^180^ | Canada | 46 | 73.2 ± 7.3 |  | 0.0% | Prostate | Androgen Deprivation Therapy |
| Trinh 2022 ^181^ | Canada, United States of America | 106 | 72.2 ± 7.6 |  | 0.0% | Prostate | Androgen Deprivation Therapy |
| Ungar 2016 ^182^ | Germany | 67 | 55.5 ± 12.6 |  | 52.2% | Any | Any |
| Vallance 2007 ^184^ | Canada | 377 | 58.0 |  | 100.0% | Breast | None |
| Vallance 2008 ^183^ | Canada | 377 | 58.0 |  | 100.0% | Breast | None |
| Vallance 2016 ^185^ | Canada | 95 | 52.8 ± 9.8 |  | 100.0% | Breast | Chemotherapy |
| Van Blarigan 2019 ^186^ | United States of America | 41 | 54.0 ± 11.0 |  | 58.5% | Colon, Rectal | None |
| Van Blarigan 2022 ^187^ | United States of America | 44 |  | 54.0 (45.0-62.0) | 56.8% | Colon, Rectal | Chemotherapy |
| Van Dam 2001 ^188^ | The Netherlands | 20 |  | 49.0 (18.0-69.0) | 50.0% | Bone | Limb-Salvage Surgery, Amputation, Rotationplasty |
| van der Stam 2021 ^189^ | The Netherlands | 27 | 64.0 ± 11.0 |  | 44.4% | Any | Major Abdominal Oncological Surgery |
| van de Wiel 2021 ^190^ | The Netherlands | 137 | 59.4 ± 12.5 |  | 48.9% | Breast, Prostate | Any Primary Curative Treatment |
| Van Dijk-Lokkart 2019 ^191^ | The Netherlands | 68 | 13.2 ± 3.1 |  | 47.1% | Any | Chemotherapy Or Radiotherapy |
| Vermaete 2014 ^192^ | Belgium | 29 | 56.0 ± 15.0 |  | 13.8% | Lymphoid | Chemotherapy |
| Von Gruenigen 2011 ^193^ | United States of America | 27 | 59.6 ± 9.2 |  | 100.0% | Ovarian, Fallopian Tube, Peritoneal | Chemotherapy |
| Waliany 2014 ^194^ | United States of America | 25 | 52.3 |  | 100.0% | Breast | Chemotherapy |
| Walsh 2021 ^195^ | Ireland | 123 | 57.4 ± 8.1 |  | 74.0% | Any | None |
| Wang 2021 ^196^ | United States of America | 49 | 55.8 ± 10.0 |  | 100.0% | Breast | None |
| Ward 2021 ^197^ | United States of America | 80 |  | 59.5 (29.0-90.0) | 45.0% | Colorectal | Chemotherapy, Curative Resection |
| Weiner 2019 ^198^ | United States of America | 87 | 57.2 ± 10.4 |  | 100.0% | Breast | None |
| Welch 2019 ^199^ | United States of America | 753 | 56.4 ± 9.5 |  | 100.0% | Breast | None |
| Wiestad 2020 ^200^ | Sweden | 55 | 58.8 ± 11.1 |  | 80.8% | Breast, Colorectal, Prostate | Chemotherapy, Endocrine Therapy, Radiotherapy |
| Wilson 2005 ^201^ | United States of America | 22 | 55.0 |  | 100.0% | Breast | None |
| Withycombe 2022 ^202^ | United States of America | 65 | 13.4 ± 2.8 |  | 53.1% | Any | Chemotherapy, Radiation, Bone Marrow Transplant |
| Wolin 2012 ^203^ | United States of America | 16 | Not reported |  | 81.3% | Colon | Colon Resection |
| Wolvers 2017 ^204^ | The Netherlands | 172 | 55.8 ± 10.2 |  | 72.0% | Any | Any |
| Wright 2018 ^205^ | United States of America | 10 | 60.0 ± 11.0 |  | 100.0% | Gynecologic | Chemotherapy |
| Xu 2021 ^206^ | China | 91 | 48.6 ± 8.0 |  | 100.0% | Breast | Surgery, Radiotherapy, Chemotherapy |
| Yonenaga 2021 ^207^ | Japan | 21 |  | 75.0 | 38.1% | Lung | Chemotherapy |
| Zahiri 2019 ^208^ | United States of America | 139 | 70.4 ± 9.7 |  | 65.5% | Any | Chemotherapy |

**Table S2.** Wearable Device Characteristics, by Study

| **Study** | **Type of Wearable** | **Brand of Wearable Device** | **Purpose of Wearable Device** | **Pattern of Use of Wearable Device** | **Timing of Use of Wearable Device** | **Duration of Wearable Device Use** | **Monitoring of Wearable Device Data** | **Adherence of Wearable Device Use** | **Intervention or Monitoring Intent** | **Clinical Outcome** | **Degree of Significance with Clinical Outcome** |  |
| --- | --- | --- | --- | --- | --- | --- | --- | --- | --- | --- | --- | --- |
| Primary Use: Prognostication | | | | | | | | | | | | |
| Au 2019 ^10^ | Pedometer | Actiwatch 2 | Daily Minutes of Physical Activity | Continuous | Post-Treatment | 1 week | Retrospective Monitoring | 84% | Intervention | Length of stay | >0.05 (inversely correlated) |  |
| Barkley 2019 ^17^ | Pedometer | Fitbit | Daily Step Count | Continuous | Pre-Treatment; On Treatment; Post-Treatment | 25 days | Retrospective Monitoring | 82% | Monitoring | Functional recovery |  |  |
| Bille 2021 ^21^ | Pedometer | 3D TriSport | Daily Step Count | Continuous | Pre-Treatment | 15 days | Retrospective Monitoring | 87% | Monitoring | Hospital length of stay, cardiac and respiratory complications | Length of stay - >0.05  Complications - <0.05 (inversely correlated) |  |
| Cos 2021 ^35^ | Pedometer | Fitbit | Daily Step Count | Continuous | Pre-Treatment; On Treatment; Post-Treatment | 2 weeks | Live Monitoring | 100% | Monitoring | Treatment failure |  |  |
| Dore 2022 ^38^ | Pedometer | ActiGraph | Daily Minutes of Physical Activity | Discrete Timepoints | Post-Treatment | 7 days every 3 months for 1 year and at years 2 and 4 | Retrospective Monitoring | Not reported | Monitoring | Depression, pain, fatigue | Depression - <0.05 (inversely correlated)  Pain - <0.05 (inversely correlated)  Fatigue - >0.05 |  |
| Finley 2020 ^49^ | Pedometer; Heart Rate Monitor | Garmin | Daily Minutes of Physical Activity and Heart Rate | Continuous | Pre-Treatment; Post-Treatment | Varying by patient (pre and postoperatively) | Retrospective Monitoring | 75% | Intervention | Exercise |  |  |
| Hamari 2019 ^71^ | Pedometer | Fitbit | Daily Minutes of Physical Activity | Continuous | On Treatment | 1 week | Retrospective Monitoring | 77% | Intervention | Physical activity, motor performance, fatigue |  |  |
| Hartman 2017 ^75^ | Pedometer | ActiGraph | Daily Minutes of Physical Activity | Continuous | Post-Treatment | 7 days | Retrospective Monitoring | 97% | Monitoring | Quality of life | 0.03 (proportionally correlated) |  |
| Kong 2020 ^92^ | Pedometer | Fitbit | Daily Step Count and Daily Minutes of Physical Activity | Continuous | Pre-Treatment | 7 days | Retrospective Monitoring | 90% | Monitoring | Sarcopenia, physical fitness |  |  |
| Mylius 2021 ^117^ | Pedometer | ActiGraph | Daily Step Count | Continuous | Pre-Treatment | 7 days | Retrospective Monitoring | Not reported | Monitoring | Functional recovery | <0.01 (proportionally correlated) |  |
| Ohri 2019 ^123^ | Pedometer | Garmin | Daily Minutes of Physical Activity | Discrete Timepoints | Pre-Treatment; On Treatment | Varying (median of 17 days) | Retrospective Monitoring | Not reported | Monitoring | Survival | <0.01 (proportionally correlated) |  |
| Pavic 2020 ^129^ | Pedometer | Biovotion AG | Daily Minutes of Physical Activity | Continuous | Post-Treatment | 12 weeks | Retrospective Monitoring | 53% | Monitoring | Readmission | <0.05 (proportionally correlated) |  |
| Richards 2020 ^148^ | Pedometer | Garmin | Daily Step Count | Discrete Timepoints | Pre-Treatment | 90 days | Retrospective Monitoring | Not reported | Monitoring | Length of stay, post-operative complications, discharge to care facilities, requiring support on discharge | Length of stay - <0.01 (inversely correlated)  Major post-operative complication - 0.04 (inversely correlated)  Discharge to care facilities - <0.01 (inversely correlated)  Requiring support on discharge – 0.03 (inversely correlated) |  |
| Saito 2020 ^162^ | Pedometer | Lifecorder Ex 4-s version | Daily Step Count and Minutes of Physical Activity | Continuous | On Treatment | 9 days | Retrospective Monitoring | Not reported | Monitoring | Bone health | Bone formation – 0.02 (inversely correlated)  Bone absorption – 0.03 (inversely correlated) |  |
| Shih 2021 ^168^ | Heart Rate Monitor | ViPCare | Heart Rate | Continuous | On Treatment | 7 days | Retrospective Monitoring | Not reported | Monitoring | Cancer-related fatigue |  |  |
| Slade 2021 ^172^ | Pedometer; Heart Rate Monitor | Garmin | Daily Step Count and Heart Rate | Continuous | On Treatment; Post-Treatment | 30 days | Retrospective Monitoring | Not reported | Monitoring | Adverse events | 0.05 (inversely correlated) |  |
| Timmerman 2018 ^178^ | Pedometer | Inertia Technology | Daily Minutes of Physical Activity | Continuous | Pre-Treatment; Post-Treatment | Minimum of 3 days at 1 and 6 months | Retrospective Monitoring | Not reported | Monitoring | Physical activity | 0.03 (proportionally correlated) |  |
| Yonenaga 2021 ^207^ | Pedometer | Suzuken Co., Ltd | Daily Step Count | Continuous | On Treatment | 7 days | Retrospective Monitoring | Not reported | Monitoring | Disability-free survival, hospital length of stay, inpatient care cost | Disability-free survival - <0.05 (inversely correlated)  Hospital length of stay - >0.05  Inpatient care cost - >0.05 |  |
| Zahiri 2019 ^208^ | Pedometer | Biosensics | Gait/Balance | Discrete Timepoints | On Treatment; Post-Treatment |  | Retrospective Monitoring | Not reported | Monitoring | Motor deterioration | <0.001 |  |

| Primary Use: Treatment Monitoring | | | | | | | | | | | |
| --- | --- | --- | --- | --- | --- | --- | --- | --- | --- | --- | --- |
| Backman 2014 ^12^ | Pedometer | SILVA Ex Connect | Daily Minutes of Physical Activity | Continuous | On Treatment | 10 weeks | Retrospective Monitoring | 91% | Intervention | Symptoms | <0.05 (inversely correlated) |
| Bade 2018 ^14^ | Pedometer | Fitbit | Daily Step Count | Continuous | On Treatment; Post-Treatment | 7 days | Retrospective Monitoring | 86% | Intervention | Physical activity participation |  |
| Bade 2021 ^15^ | Pedometer | Fitbit | Daily Minutes of Physical Activity | Continuous | On Treatment | 12 weeks | Retrospective Monitoring | 90% | Intervention | Quality of life |  |
| Braam 2016 ^23^ | Pedometer | Actical Activity Monitor B Series | Daily Minutes of Physical Activity | Continuous | On Treatment; Post-Treatment | 4 days | Retrospective Monitoring | 88% | Monitoring | Physical activity, sedentary behavior, cardiorespiratory fitness |  |
| Broderick 2019 ^26^ | Pedometer | Microsoft | Daily Step Count | Continuous | On Treatment; Post-Treatment | 60 days | Retrospective Monitoring | 86% | Monitoring | Performance status |  |
| Cadmus-Bertram 2019 ^28^ | Pedometer | ActiGraph | Daily Minutes of Physical Activity | Continuous | Post-Treatment | 12 weeks | Retrospective Monitoring | Not reported | Intervention | Physical activity | <0.05 (proportionally correlated) |
| Champ 2018 ^31^ | Pedometer | Misfit | Daily Step Count | Continuous | Pre-Treatment; On Treatment; Post-Treatment | 50 days | Retrospective Monitoring | Not reported | Monitoring | Physical activity, sleep, fatigue, body mass index | >0.05 |
| Chestnut 2020 ^33^ | Pedometer; Continuous Glucose Monitor | Garmin | Daily Step Count | Continuous | Pre-Treatment; On Treatment; Post-Treatment | 12 months | Retrospective Monitoring | Not reported | Intervention | Glycemic control |  |
| Dorion 2017 ^39^ | Pedometer | Misfit | Daily Step Count | Continuous | Pre-Treatment; On Treatment; Post-Treatment | 1 month | Retrospective Monitoring | 92% | Monitoring | Quality of life, pain, performance status | Quality of life - >0.05  Pain - >0.05  Performance status – 0.04 (proportionally correlated) |
| Erickson 2021 ^44^ | Pedometer | ActiGraph | Daily Minutes of Physical Activity | Continuous | On Treatment |  | Live Monitoring | 84% | Intervention | Fatigue, self-efficacy |  |
| Fouladiun 2007 ^51^ | Pedometer | ActiGraph | Daily Step Count | Continuous | On Treatment; Post-Treatment | 3 days every 4 months for 1 year | Retrospective Monitoring | 56% | Monitoring | Weight loss, physical functioning, pain | <0.05 (inversely correlated) |
| Ghods 2021 ^56^ | Pedometer | Misfit | Daily Step Count | Discrete Timepoints | On Treatment | 21 days | Retrospective Monitoring | 68% | Monitoring | Performance status, symptoms | Performance status - <0.05 (proportionally correlated)  Symptoms - >0.05 |
| Gotte 2018 ^60^ | Pedometer | Fitbit | Daily Minutes of Physical Activity | Continuous | On Treatment | 8-10 weeks | Retrospective Monitoring | 93% | Intervention | Quality of life, motor performance |  |
| Gresham 2018 ^62^ | Pedometer | Fitbit | Daily Minutes of Physical Activity | Discrete Timepoints | On Treatment | 7 days twice in 1 year | Retrospective Monitoring | 92% | Monitoring | Performance status, adverse events, hospitalizations, survival, fatigue, sleep | Performance status - <0.01 (proportionally correlated)  Adverse events – <0.05 (inversely correlated)  Hospitalizations - <0.05 (inversely correlated)  Survival - <0.05 (inversely correlated)  Fatigue and sleep - >0.05 |
| Gresham 2021 ^63^ | Pedometer; Heart Rate Monitor | Fitbit | Daily Minutes of Physical Activity | Continuous | On Treatment | 3 months | Live Monitoring | 94% | Monitoring | Weight stability, lean body mass, quality of life, depression, pain |  |
| Guest 2013 ^64^ | Pedometer | ActiGraph | Daily Step Count and Daily Minutes of Physical Activity | Continuous | On Treatment |  | Retrospective Monitoring | 100% | Monitoring | Fatigue, diet, body mass index | >0.05 |
| Gupta 2018 ^67^ | Pedometer | Fitbit | Daily Step Count and Daily Minutes of Physical Activity | Continuous | On Treatment | 4 weeks | Retrospective Monitoring | 96% | Monitoring | Performance status, quality of life | Performance status - <0.01 (proportionally correlated)  Quality of life – 0.02 (proportionally correlated) |
| Gururaj 2021 ^68^ | Pedometer | ActiGraph | Daily Step Count | Discrete Timepoints | On Treatment | 7 weeks | Retrospective Monitoring | Not reported | Monitoring | Physical activity, sleep efficiency | <0.05 |
| Hacker 2018 ^69^ | Pedometer | Actical | Daily Step Count | Continuous | Pre-Treatment; Post-Treatment | 35 days | Retrospective Monitoring | 91% | Intervention | Fatigue, quality of life |  |
| Hooke 2016 ^78^ | Pedometer | Fitbit | Daily Step Count | Continuous | On Treatment | 22 days | Retrospective Monitoring | 94% | Intervention | Physical activity |  |
| Hooke 2019 ^79^ | Pedometer | ActiGraph | Daily Step Count | Discrete Timepoints | On Treatment | 3 days every 2 months for 6 months | Retrospective Monitoring | Not reported | Intervention | Physical activity, fatigue |  |
| Janssen 2021 ^83^ | Pedometer | ActivPAL | Daily Minutes of Physical Activity | Continuous | On Treatment | 7 days | Retrospective Monitoring | Not reported | Monitoring | Fatigue | <0.02 (inversely correlated) |
| Javaheri 2015 ^84^ | Pedometer | StepsCount | Daily Step Count | Continuous | On Treatment | 2 weeks | Retrospective Monitoring | 91% | Intervention | Physical fitness, happiness | <0.01 (proportionally correlated) |
| Johnson 2021 ^87^ | Pedometer | Fitbit | Daily Step Count | Continuous | On Treatment; Post-Treatment | 12 weeks | Retrospective Monitoring | 83% | Intervention | Fatigue, self-determination, quality of life |  |
| Jonsson 2019 ^89^ | Pedometer | ActiGraph | Daily Minutes of Physical Activity | Continuous | On Treatment; Post-Treatment | 3 days post-operatively, 7 days 3 months later | Retrospective Monitoring | Not reported | Intervention | Physical activity, physical fitness |  |
| Jonsson 2019 ^90^ | Pedometer | ActiGraph | Daily Minutes of Physical Activity | Continuous | On Treatment; Post-Treatment | Min 3 days postoperatively | Retrospective Monitoring | Not reported | Intervention | Physical activity, physical fitness, dyspnea |  |
| Koontz 2021 ^93^ | Pedometer | Fitbit | Daily Step Count and Daily Minutes of Physical Activity | Continuous | On Treatment; Post-Treatment | 12 weeks | Retrospective Monitoring | 72% | Intervention | Physical activity, physical fitness, depression, quality of life |  |
| Long 2018 ^96^ | Pedometer | Actical | Daily Minutes of Physical Activity | Continuous | On Treatment; Post-Treatment | 7 days | Retrospective Monitoring | 100% | Intervention | Physical fitness |  |
| Low 2021 ^99^ | Pedometer; Heart Rate Monitor | Fitbit | Daily Step Count and Heart Rate | Continuous | Pre-Treatment; On Treatment | Varying (mean of 1353 days) | Retrospective Monitoring | Not reported | Monitoring | Symptom burden | <0.05 (inversely correlated) |
| Lowe 2015 ^100^ | Pedometer | ActivPAL | Daily Step Count and Daily Minutes of Physical Activity | Continuous | On Treatment | 7 days | Retrospective Monitoring | 100% | Monitoring | Physical activity, attitude | <0.05 (proportionally correlated) |
| Maddocks 2009 ^103^ | Pedometer | ActivPAL | Daily Step Count | Continuous | Pre-Treatment | 7 days | Retrospective Monitoring | 100% | Intervention | Physical fitness |  |
| Maddocks 2010 ^104^ | Pedometer | ActivPAL | Daily Minutes of Physical Activity | Continuous | Post-Treatment | 10 days | Retrospective Monitoring | Not reported | Monitoring | Physical activity |  |
| Marinac 2015 ^106^ | Pedometer | ActiGraph | Daily Minutes of Physical Activity | Continuous | Pre-Treatment; Post-Treatment | 7 days | Retrospective Monitoring | Not reported | Monitoring | Physical activity, cognitive function, body mass index | Cognitive function – 0.04 (proportionally correlated)  Body mass index – 0.02 (inversely correlated) |
| Martin 2020 ^107^ | Pedometer | E-care Fit | Daily Step Count | Continuous | Pre-Treatment; On Treatment; Post-Treatment | 8 days | Retrospective Monitoring | 85% | Monitoring | Physical activity, treatment complications, prolonged length of stay | Treatment complication - >0.05  Prolonged length of stay - <0.01 (inversely correlated) |
| Matsui 2021 ^108^ | Pedometer | Active Style Pro HJA-750C | Daily Minutes of Physical Activity | Discrete Timepoints | Pre-Treatment; On Treatment; Post-Treatment | 1-day pre and post-surgery, and 6 months post-surgery | Retrospective Monitoring | 71% | Monitoring | Physical activity, sedentary behavior, skeletal muscle composition |  |
| Nelson 2021 ^119^ | Pedometer | Fitbit | Daily Minutes of Physical Activity | Discrete Timepoints | Pre-Treatment; On Treatment; Post-Treatment | Varying (mean of 17 weeks) | Retrospective Monitoring | Not reported | Monitoring | Physical activity |  |
| Nilanon 2020 ^120^ | Pedometer; Heart Rate Monitor | Microsoft | Daily Step Counts and Heart Rate | Discrete Timepoints | On Treatment | 60 days | Retrospective Monitoring | 68% | Monitoring | Performance status | <0.01 (proportionally correlated) |
| Nyrop 2018 ^121^ | Pedometer | Fitbit | Daily Step Count | Discrete Timepoints | On Treatment | Varying (6-12 weeks) | Retrospective Monitoring | 79% | Intervention | Physical activity, quality of life, anxiety | Physical activity - <0.01 (proportionally correlated)  Quality of life – 0.02 (proportionally correlated)  Anxiety – <0.01 (inversely correlated) |
| Ohri 2017 ^122^ | Pedometer | Garmin | Daily Minutes of Physical Activity | Discrete Timepoints | On Treatment | Varying | Retrospective Monitoring | 94% | Monitoring | Hospitalization risk | <0.01 (inversely correlated) |
| Paul 2020 ^128^ | Pedometer | Garmin | Daily Step Count | Continuous | On Treatment | 4-7 weeks | Retrospective Monitoring | Not reported | Monitoring | Performance status | >0.05 |
| Piringer 2020 ^139^ | Pedometer | HMM Diagnostics | Daily Step Count | Discrete Timepoints | On Treatment | 1-week intervals for varying periods | Retrospective Monitoring | 88% | Monitoring | Physical activity |  |
| Pope 2018 ^140^ | Pedometer | ActiGraph | Daily Minutes of Physical Activity | Continuous | Post-Treatment | 7 days followed by 7 days 10 weeks later | Retrospective Monitoring | Not reported | Intervention | Physical activity, energy expenditure | Physical activity – >0.05  Energy expenditure - >0.05 |
| Porserud 2019 ^141^ | Pedometer | ActivPAL | Daily Minutes of Physical Activity | Discrete Timepoints | On Treatment | 3 days | Retrospective Monitoring | 89% | Intervention | Mobilisation, length of stay | Mobilisation – <0.01 (proportionally correlated)  Length of stay – 0.03 (inversely correlated) |
| Prinsen 2013 ^142^ | Pedometer | Not reported | Daily Minutes of Physical Activity | Discrete Timepoints | Post-Treatment | 12 days during registration, baseline, and at 6 months. | Retrospective Monitoring | Not reported | Intervention | Physical activity | >0.05 |
| Rahimy 2021 ^145^ | Pedometer; Heart Rate Monitor | Fitbit | Daily Step Count | Continuous | Post-Treatment | 21 weeks | Retrospective Monitoring | Not reported | Intervention | Physical activity |  |
| Rehorst-Kleinlugtenbelt 2019 ^147^ | Pedometer | Philips Respironics | Daily Minutes of Physical Activity | Discrete Timepoints | On Treatment | 7 days | Retrospective Monitoring | 81% | Monitoring | Physical activity |  |
| Sande 2014 ^163^ | Pedometer | ActivPAL | Daily Minutes of Physical Activity | Discrete Timepoints | On Treatment |  | Retrospective Monitoring | Not reported | Monitoring | Cancer-induced bone pain | <0.001 (inversely correlated) |
| Schink 2020 ^164^ | Pedometer | Shimmer Research Ltd | Daily Minutes of Physical Activity | Discrete Timepoints | On Treatment | Twice a week for 12 weeks | Retrospective Monitoring | 51% | Intervention | Gait | <0.05 (proportionally correlated) |
| Shah 2021 ^167^ | Pedometer | Fitbit | Daily Step Count | Continuous | On Treatment | Varying | Retrospective Monitoring | Not reported | Monitoring | Rates of triage visits | 0.02 (inversely correlated) |
| Singh 2020 ^170^ | Pedometer | ActiGraph, Fitbit | Daily Step Count and Minutes of Physical Activity | Continuous | On Treatment; Post-Treatment | 12 weeks | Retrospective Monitoring | Not reported | Intervention | Physical activity | <0.01 (proportionally correlated) |
| Skipworth 2011 ^171^ | Pedometer | ActivPAL | Daily Minutes of Physical Activity | Continuous | On Treatment; Post-Treatment | 2 weeks | Retrospective Monitoring | Not reported | Monitoring | Physical activity |  |
| Strother 2021 ^175^ | Pedometer | Fitbit | Daily Step Count | Continuous | Pre-Treatment; Post-Treatment | Between 33 and 44 days | Retrospective Monitoring | 97% | Intervention | Physical activity | >0.05 |
| Trinh 2018 ^180^ | Pedometer | ActiGraph | Daily Step Count and Minutes of Physical Activity | Continuous | On Treatment | 12 weeks | Retrospective Monitoring | 72% | Intervention | Sedentary behavior, physical activity | Sedentary behavior – <0.01 (inversely correlated)  Physical activity – 0.01 (proportionally correlated) |
| Trinh 2022 ^181^ | Pedometer | ActiGraph | Daily Minutes of Physical Activity | Continuous | On Treatment |  | Retrospective Monitoring | 96% | Monitoring | Quality of life | 0.01 (proportionally correlated) |
| Ungar 2016 ^182^ | Pedometer | ActiGraph | Daily Minutes of Physical Activity | Continuous | On Treatment; Post-Treatment | 2 weeks followed by a week 10 weeks later. | Retrospective Monitoring | Not reported | Intervention | Physical activity | 0.02 (proportionally correlated) |
| Vallance 2016 ^185^ | Pedometer | StepsCount | Daily Step Count | Continuous | On Treatment | 4-6 months | Retrospective Monitoring | 95% | Intervention | Physical activity | >0.05 |
| Van Blarigan 2022 ^187^ | Pedometer | Fitbit, ActiGraph | Daily Step Count and Minutes of Physical Activity | Continuous | On Treatment | 12 weeks | Retrospective Monitoring | 91% | Intervention | Physical activity | >0.05 |
| Van Dam 2001 ^188^ | Pedometer | McRoberts BV | Daily Minutes of Physical Activity | Continuous | Post-Treatment | 2 days and another 2 days 1-2 weeks later | Retrospective Monitoring | Not reported | Monitoring | Physical activity |  |
| van der Stam 2021 ^189^ | Heart Rate Monitor | Healthdot | Heart Rate | Continuous | Post-Treatment | 2 weeks | Retrospective Monitoring | 74% | Monitoring | Heart rate, respiration rate |  |
| Van Dijk-Lokkart 2019 ^191^ | Pedometer | Philips Respironics | Daily Step Count | Continuous | On Treatment; Post-Treatment | 4 days at 0, 4 and 12 months | Retrospective Monitoring | Not reported | Monitoring | Physical activity | >0.05 |
| Vermaete 2014 ^192^ | Pedometer | DynaPort MiniMod | Daily Minutes of Physical Activity | Continuous | Pre-Treatment; On Treatment; Post-Treatment | Median of 15 weeks | Retrospective Monitoring | Not reported | Monitoring | Physical activity, physical fitness, fatigue | Physical activity – <0.01 (proportionally correlated)  Physical fitness - <0.01 (proportionally correlated)  Fatigue – <0.01 (inversely correlated) |
| Von Gruenigen 2011 ^193^ | Pedometer | New Lifestyles | Daily Step Count | Continuous | On Treatment | Varying (based on chemotherapy cycles) | Retrospective Monitoring | 92% | Intervention | Physical activity, diet | Physical activity - >0.05  Diet - >0.05 |
| Waliany 2014 ^194^ | Pedometer | Bodybugg | Daily Minutes of Physical Activity | Continuous | Pre-Treatment; Post-Treatment | 7 weeks | Retrospective Monitoring | Not reported | Monitoring | Metabolic equivalent of task | <0.01 (inversely correlated) |
| Ward 2021 ^197^ | Pedometer | Fitbit | Daily Step Count | Continuous | On Treatment | 4 days | Retrospective Monitoring | 85% | Monitoring | Physical activity | <0.01 (proportionally correlated) |
| Withycombe 2022 ^202^ | Pedometer | Garmin | Daily Step Count | Continuous | On Treatment | 7 days and y days 4+ weeks later | Retrospective Monitoring | 94% | Monitoring | Physical activity | <0.01 (proportionally correlated) |
| Wolin 2012 ^203^ | Pedometer | ActiGraph | Daily Minutes of Physical Activity | Continuous | Post-Treatment | 12 weeks | Retrospective Monitoring | 81% | Intervention | Physical activity | 0.01 (proportionally correlated) |
| Wolvers 2017 ^204^ | Pedometer | ProMove 3D | Daily Minutes of Physical Activity | Continuous | Post-Treatment | 4 days | Retrospective Monitoring | Not reported | Monitoring | Physical activity |  |
| Wright 2018 ^205^ | Pedometer | Fitbit | Daily Step Count and Heart Rate | Continuous | On Treatment | 3 weeks | Retrospective Monitoring | 83% | Monitoring | Physical activity |  |

| Primary Use: Rehabilitation | | | | | | | | | | | |
| --- | --- | --- | --- | --- | --- | --- | --- | --- | --- | --- | --- |
| Awick 2017 ^11^ | Pedometer | ActiGraph | Daily Minutes of Physical Activity | Continuous | Post-Treatment | 3 days at 0 and 6 months | Retrospective Monitoring | Not reported | Monitoring | Self-esteem | <0.05 (proportionally correlated) |
| Bade 2018 ^13^ | Pedometer | Fitbit | Daily Step Count | Continuous | On Treatment | 7 days | Retrospective Monitoring | 67% | Monitoring | Quality of life | <0.05 (proportionally correlated) |
| Ballinger 2021 ^16^ | Pedometer | Garmin | Daily Step Count | Continuous | On Treatment | 12 weeks | Retrospective Monitoring | 95% | Intervention | Energetic capacity, energy expenditure, quality of life |  |
| Bekkering 2012 ^18^ | Pedometer | ActiLog | Number of high (peak) activity periods throughout the day | Continuous | Post-Treatment | 7 days at months 3, 6, 9, 12, 18 and 24 | Retrospective Monitoring | Not reported | Monitoring | Quality of life |  |
| Bender 2021 ^19^ | Pedometer | SenseWear | Daily Minutes of Physical Activity | Continuous | Pre-Treatment | 7 days | Retrospective Monitoring | Not reported | Monitoring | Cognitive function |  |
| Bernard 2016 ^20^ | Pedometer | Actiwatch 64 | Daily Step Count and Minutes of Physical Activity | Continuous | Post-Treatment | 7 days | Retrospective Monitoring | 86% | Monitoring | Sleep |  |
| Boyle 2017 ^22^ | Pedometer | ActiGraph | Daily Minutes of Physical Activity | Continuous | Post-Treatment |  | Retrospective Monitoring | 70% | Monitoring | Physical activity, sedentary time |  |
| Breedveld-Peters 2018 ^24^ | Pedometer | MMOXX1 | Daily Minutes of Physical Activity | Continuous | Post-Treatment | 7 days | Retrospective Monitoring | Not reported | Intervention | Quality of life |  |
| Broderick 2014 ^25^ | Pedometer | RT3 Accelerometer | Daily Minutes of Physical Activity | Discrete Timepoints | Post-Treatment | 60 days | Retrospective Monitoring | Not reported | Monitoring | Physical activity, sedentary behavior, quality of life |  |
| Bulls 2019 ^27^ | Pedometer | ActiGraph | Daily Step Count | Continuous | On Treatment; Post-Treatment | 7 days at cycle 1 and 6, 6 months and 12 months | Retrospective Monitoring | 100% | Monitoring | Chemotherapy-induced peripheral neuropathy, sleep quality, physical activity |  |
| Caperchione 2019 ^29^ | Pedometer | ActiGraph | Daily Minutes of Physical Activity | Continuous | Post-Treatment | 7 days | Retrospective Monitoring | 74% | Intervention | Physical activity, quality of life | <0.05 (proportionally correlated) |
| Carter 2016 ^30^ | Heart Rate Monitor | Polar Electro | Heart Rate During Workouts | Continuous | Post-Treatment | 12 weeks | Retrospective Monitoring | 69% | Intervention | Fatigue, peak oxygen uptake, resting rate blood pressure | Fatigue - >0.05  Peak oxygen uptake - <0.01 (proportionally correlated)  Resting rate blood pressure - <0.05 (inversely correlated) |
| Chan 2022 ^32^ | Pedometer | ActiGraph | Daily Minutes of Physical Activity | Continuous | Post-Treatment | Median 74 days | Retrospective Monitoring | Not reported | Intervention | Quality of life |  |
| Chow 2021 ^34^ | Pedometer | Fitbit | Daily Step Count | Continuous | Post-Treatment | 16 weeks | Retrospective Monitoring | Not reported | Intervention | Dietary factors (sugar, saturated fat, sodium), quality of life, self-efficacy | >0.05 |
| Dennett 2018 ^36^ | Pedometer | ActivPAL | Daily Minutes of Physical Activity | Continuous | On Treatment; Post-Treatment | 7 days at 0 and 8 weeks | Retrospective Monitoring | 92% | Intervention | Physical activity |  |
| Devine 2020 ^37^ | Pedometer | ActiGraph | Daily Minutes of Physical Activity | Continuous | Post-Treatment | 7 days at 0, 2, 3, 6, and 9 months | Retrospective Monitoring | Not reported | Intervention | Physical fitness, quality of life, fatigue |  |
| Douma 2018 ^40^ | Pedometer | ActiGraph | Daily Step Count | Continuous | On Treatment; Post-Treatment | 14 days | Retrospective Monitoring | Not reported | Monitoring | Physical activity |  |
| Edbrooke 2019 ^41^ | Pedometer | SenseWear | Daily Step Count | Continuous | On Treatment; Post-Treatment | 7 days at 0 and 9 weeks, 6 months | Retrospective Monitoring | 87% | Monitoring | Physical fitness, quality of life, anxiety, depression, symptoms | Physical fitness - <0.01 (proportionally correlated)  Quality of life - <0.01 (proportionally correlated)  Anxiety – 0.87  Depression – 0.01 (inversely correlated)  Symptoms - <0.01 (inversely correlated) |
| Ehlers 2017 ^42^ | Pedometer | ActiGraph | Daily Minutes of Physical Activity | Continuous | Post-Treatment | 7 days | Live Monitoring | Not reported | Monitoring | Executive function, working memory | <0.05 (proportionally correlated) |
| Ehlers 2018 ^43^ | Pedometer | ActiGraph | Daily Minutes of Physical Activity | Continuous | Post-Treatment | 7 days | Retrospective Monitoring | 95% | Monitoring | Cognition | <0.05 (proportionally correlated) |
| Fazzino 2017 ^45^ | Pedometer | ActiGraph | Daily Minutes of Physical Activity | Discrete Timepoints | Post-Treatment | 7 days at 0, 6, 12 and 18 months | Retrospective Monitoring | 68% | Intervention | Weight loss | <0.05 (proportionally correlated) |
| Fazzino 2018 ^46^ | Pedometer | ActiGraph | Daily Minutes of Physical Activity | Discrete Timepoints | Post-Treatment | 7 days at 0, 6, 12, and 18 months | Retrospective Monitoring | Not reported | Intervention | Quality of life | <0.05 (proportionally correlated) |
| Ferrante 2020 ^47^ | Pedometer | Fitbit | Daily Step Count | Continuous | Post-Treatment | 7 days at 0, 6 and 12 months | Retrospective Monitoring | Not reported | Intervention | Quality of life |  |
| Ferrante 2022 ^48^ | Pedometer | Fitbit | Daily Step Count | Continuous | Post-Treatment | 12 months | Retrospective Monitoring | 77% | Intervention | Physical activity, self-efficacy | <0.05 (proportionally correlated) |
| Finley 2021 ^50^ | Pedometer; Heart Rate Monitor | Garmin | Daily Step Count, Minutes of Physical Activity and Heart Rate | Continuous | Pre-Treatment | Median 18.5 days | Retrospective Monitoring | 60% | Intervention | Aerobic capacity | >0.05 |
| Frensham 2020 ^52^ | Pedometer | New-Lifestyles NL-1000 | Daily Step Count | Continuous | Post-Treatment | 12 weeks | Retrospective Monitoring | Not reported | Intervention | Motivation, self-efficacy |  |
| Gaskin 2016 ^53^ | Pedometer | ActiGraph | Daily Minutes of Physical Activity | Continuous | Post-Treatment | 7 days at 0 and 12 weeks | Retrospective Monitoring | 67% | Monitoring | Quality of life, physical functioning | >0.05 |
| Gell 2017 ^54^ | Pedometer | ActiGraph | Daily Step Count and Minutes of Physical Activity | Continuous | Post-Treatment | 12 weeks | Retrospective Monitoring | 100% | Intervention | Self-efficacy, self-regulation, social support, fatigue, depression |  |
| Gell 2019 ^55^ | Pedometer | Fitbit | Daily Minutes of Physical Activity | Continuous | Post-Treatment | 8 weeks | Live Monitoring | 84% | Intervention | Exercise maintenance |  |
| Gielissen 2012 ^57^ | Pedometer | Not reported | Daily Minutes of Physical Activity | Continuous | Post-Treatment | 12 days at 0 and 6 months | Retrospective Monitoring | 85% | Intervention | Fatigue | >0.05 |
| Gilchrist 2019 ^58^ | Pedometer | ActiGraph | Daily Minutes of Physical Activity | Continuous | Post-Treatment | 7 days every 3 months for a year | Retrospective Monitoring | 99% | Monitoring | Alcohol consumption | 0.02 (inversely correlated) |
| Gomersall 2019 ^59^ | Pedometer | ActivPAL | Daily Minutes of Physical Activity | Continuous | Post-Treatment | 7 days at 0, 4, and 12 months | Retrospective Monitoring | 86% | Intervention | Physical activity |  |
| Gregoire 2020 ^61^ | Pedometer | Garmin | Daily Step Count | Continuous | Post-Treatment | 8 weeks | Retrospective Monitoring | 93% | Intervention | Fatigue, sleep, emotional distress, cognitive functioning |  |
| Guinan 2013 ^65^ | Heart Rate Monitor | Polar Heart Rate Monitor; Triaxial RT3 Activity Monitor | Daily Minutes of Physical Activity and Heart Rate | Continuous | Post-Treatment | 7 days at 0, 2 and 3 months | Retrospective Monitoring | Not reported | Intervention | Physical activity |  |
| Gundle 2017 ^66^ | Pedometer | Fitbit | Daily Step Count | Continuous | Post-Treatment | Median 15.5 days | Retrospective Monitoring | Not reported | Monitoring | Physical health, mental health | Physical health – 0.04 (proportionally correlated)  Mental health – 0.66 |
| Hall 2021 ^70^ | Pedometer | Fitbit | Daily Step Count | Continuous | On Treatment | 7 days at 0 and 8 weeks | Retrospective Monitoring | 76% | Intervention | Weight change |  |
| Hardcastle 2020 ^72^ | Pedometer | ActiGraph | Daily Step Count and Daily Minutes of Physical Activity | Discrete Timepoints | Post-Treatment | 24 weeks | Retrospective Monitoring | 100% | Intervention | Physical activity |  |
| Hardcastle 2021 ^73^ | Pedometer | ActiGraph | Daily Minutes of Physical Activity | Continuous | Post-Treatment | 7 days at 0, 12 and 24 months | Retrospective Monitoring | Not reported | Intervention | Self-efficacy, outcome expectancies, risk perceptions |  |
| Hardcastle 2021 ^74^ | Pedometer | ActiGraph | Daily Minutes of Physical Activity | Continuous | Post-Treatment | 7 days at 0, 12 and 24 weeks | Retrospective Monitoring | Not reported | Monitoring | Physical activity |  |
| Hartman 2018 ^76^ | Pedometer | ActiGraph | Daily Minutes of Physical Activity | Discrete Timepoints | Post-Treatment | 12 weeks | Retrospective Monitoring | Not reported | Intervention | Physical activity | <0.01 (proportionally correlated) |
| Hirschey 2018 ^77^ | Pedometer | Fitbit | Daily Step Count | Continuous | Post-Treatment | Mean 75 days | Retrospective Monitoring | 60% | Intervention | Exercise expectations |  |
| Howell 2018 ^80^ | Pedometer | ActiGraph | Daily Minutes of Physical Activity | Discrete Timepoints | Post-Treatment | 7 days at 0, 12 and 24 weeks | Retrospective Monitoring | Not reported | Intervention | Physical fitness, cognition, quality of life |  |
| Irwin 2008 ^81^ | Pedometer | Not reported | Daily Step Count | Continuous | Post-Treatment | 7 days at 0 and 6 months | Retrospective Monitoring | Not reported | Intervention | Physical fitness |  |
| James 2015 ^82^ | Pedometer | Yamax | Daily Step Count | Discrete Timepoints | Post-Treatment | 7 days at 0, 8 and 20 weeks | Retrospective Monitoring | Not reported | Intervention | Physical activity, diet, alcohol intake, body mass index | >0.05 |
| Jeffery 2017 ^85^ | Pedometer | ActiGraph | Daily Minutes of Physical Activity | Continuous | On Treatment; Post-Treatment | 7 days | Retrospective Monitoring | Not reported | Monitoring | Performance status | <0.05 (proportionally correlated) |
| Jeffery 2022 ^86^ | Pedometer | ActiGraph | Daily Minutes of Physical Activity | Continuous | On Treatment; Post-Treatment | 3 days every 3 months for 18 months | Retrospective Monitoring | Not reported | Monitoring | Physical activity, energy intake, protein intake | >0.05 |
| Jones 2013 ^88^ | Pedometer; Heart Rate Monitor | Polar Heart Rate Monitor | Daily Step Count and Heart Rate | Continuous | Post-Treatment | 7 days at 0 and 6 months | Retrospective Monitoring | Not reported | Intervention | Interleukin-6, C-reactive protein, tumor necrosis factor α | IL-6 - <0.01 (inversely correlated)  CRP - <0.01 (inversely correlated)  TNFα – 0.38 |
| Keadle 2021 ^91^ | Pedometer | ActivPAL | Daily Step Count | Continuous | Post-Treatment | 12 weeks | Retrospective Monitoring | 57% | Intervention | Physical activity, sleep quality, fatigue |  |
| Leach 2019 ^94^ | Pedometer | Polar A300 | Daily Step Count | Continuous | Post-Treatment | 7 days at 0, 2 and 5 months | Retrospective Monitoring | Not reported | Intervention | Physical activity |  |
| Leach 2020 ^95^ | Pedometer | ActivPAL | Daily Step Count | Continuous | Pre-Treatment | 7 days | Retrospective Monitoring | 100% | Intervention | Physical activity |  |
| Loprinzi 2019 ^97^ | Pedometer | ActiGraph | Daily Minutes of Physical Activity | Continuous | Post-Treatment | 7 days | Retrospective Monitoring | 100% | Monitoring | Survival | 0.04 (proportionally correlated) |
| Loughney 2017 ^98^ | Pedometer | SenseWear | Daily Step Count and Daily Minutes of Physical Activity | Discrete Timepoints | Post-Treatment | 3 days at week 0, 3 and 6 | Retrospective Monitoring | 82% | Intervention | Physical activity, sleep |  |
| Lozano-Lozano 2019 ^101^ | Pedometer | ActiGraph | Daily Minutes of Physical Activity | Continuous | Post-Treatment | 8 days at 0 and 8 weeks | Retrospective Monitoring | Not reported | Intervention | Quality of life, interleukin-6, C-reactive protein |  |
| Lynch 2019 ^102^ | Pedometer | Garmin | Daily Step Count and Daily Minutes of Physical Activity | Continuous | Post-Treatment | 12 weeks | Retrospective Monitoring | 93% | Intervention | Physical activity, sedentary time |  |
| Maeda 2016 ^105^ | Pedometer | Actimarker | Daily Minutes of Physical Activity | Discrete Timepoints | Pre-Treatment; Post-Treatment | 5 days at 0 and 2 months | Retrospective Monitoring | Not reported | Intervention | Physical activity |  |
| Matthews 2007 ^109^ | Pedometer | ActiGraph | Daily Minutes of Physical Activity | Continuous | Post-Treatment | 7 days at 0, 6 and 12 weeks | Retrospective Monitoring | Not reported | Intervention | Physical activity, body weight, body composition |  |
| McNeil 2019 ^110^ | Pedometer | ActiGraph | Daily Minutes of Physical Activity | Continuous | Post-Treatment | 12 weeks | Retrospective Monitoring | Not reported | Intervention | Physical activity, physical fitness |  |
| Minton 2012 ^111^ | Pedometer | Actiwatch | Daily Minutes of Physical Activity | Continuous | Post-Treatment | 1 week | Retrospective Monitoring | Not reported | Monitoring | Fatigue, mood, sleep, quality of life, cognition |  |
| Miyaji 2020 ^112^ | Pedometer | Garmin | Daily Step Count | Continuous | Post-Treatment | 29 days | Live Monitoring | 90% | Monitoring | Fatigue |  |
| Modesitt 2021 ^113^ | Pedometer | Not reported | Daily Step Count | Continuous | Pre-Treatment; Post-Treatment | 7 days | Retrospective Monitoring | Not reported | Intervention | Physical fitness, quality of life, body mass index |  |
| Mouri 2018 ^114^ | Pedometer | Kenz Lifecorder-GS | Daily Step Count | Continuous | Post-Treatment | 8 weeks | Retrospective Monitoring | Not reported | Intervention | Physical activity, quality of life |  |
| Muller 2014 ^115^ | Pedometer | StepWatch | Daily Step Count | Continuous | Post-Treatment | 7 days at 6 and 12 months | Retrospective Monitoring | Not reported | Intervention | Physical activity, bone mineral content, bone mineral density |  |
| Muller 2016 ^116^ | Pedometer | StepWatch | Daily Step Count | Continuous | Post-Treatment | 7 days at 0, 1, 6 and 12 months | Retrospective Monitoring | Not reported | Intervention | Physical activity, quality of life |  |
| Naito 2019 ^118^ | Pedometer | Kenz Lifecorder-GS | Daily Step Count | Continuous | Post-Treatment | 8 weeks | Retrospective Monitoring | 98% | Intervention | Physical function |  |
| Ormel 2021 ^124^ | Pedometer | ActiGraph | Daily Minutes of Physical Activity | Continuous | On Treatment | 7 days at 0, 12 and 26 weeks | Retrospective Monitoring | Not reported | Intervention | Physical activity, body composition, quality of life, self-efficacy |  |
| Park 2019 ^125^ | Pedometer | InBody Co., Ltd | Daily Minutes of Physical Activity | Continuous | Post-Treatment | 12 weeks | Retrospective Monitoring | Not reported | Intervention | Physical function, diarrhea | Physical function - >0.05  Diarrhea – 0.03 (proportionally correlated) |
| Park 2020 ^126^ | Pedometer | ActiGraph | Daily Minutes of Physical Activity | Discrete Timepoints | On Treatment | 7 days at 0, 4, 8, 12 weeks | Retrospective Monitoring | Not reported | Intervention | Physical activity, symptoms, quality of life | Physical activity – 0.25  Symptoms – 0.25 |
| Parker 2019 ^127^ | Pedometer | ActiGraph | Daily Minutes of Physical Activity | Continuous | On Treatment | 14 days at 3 different points during chemotherapy | Retrospective Monitoring | Not reported | Intervention | Physical activity |  |
| Perkins 2009 ^130^ | Pedometer | ActiGraph | Daily Minutes of Physical Activity | Continuous | Post-Treatment | 7 days | Retrospective Monitoring | Not reported | Intervention | Physical activity, outcome expectation | >0.05 |
| Phillips 2014 ^131^ | Pedometer | Health One Technology | Daily Minutes of Physical Activity | Continuous | Post-Treatment | 7 days at 0 and 6 months | Retrospective Monitoring | Not reported | Monitoring | Quality of life | <0.01 (proportionally correlated) |
| Phillips 2015 ^132^ | Pedometer | ActiGraph | Daily Minutes of Physical Activity | Continuous | Post-Treatment | 7 days at 0 and 6 months | Retrospective Monitoring | Not reported | Monitoring | Fatigue, quality of life | Fatigue - <0.05 (inversely correlated)  Quality of life - <0.05 (proportionally correlated) |
| Phillips 2022 ^133^ | Pedometer | ActiGraph | Daily Minutes of Physical Activity | Continuous | Post-Treatment | 12 weeks | Retrospective Monitoring | 85% | Intervention | Physical activity |  |
| Pinto 2015 ^134^ | Pedometer | ActiGraph | Daily Step Count and Minutes of Physical Activity | Continuous | On Treatment | 7 days at 0, 12, and 24 months | Retrospective Monitoring | Not reported | Intervention | Physical activity | <0.05 (proportionally correlated) |
| Pinto 2017 ^135^ | Pedometer; Heart Rate Monitor | ActiGraph | Daily Step Count and Heart Rate | Continuous | On Treatment | 7 days at 0, 12, and 24 months | Retrospective Monitoring | Not reported | Intervention | Sedentary behavior | >0.05 |
| Pinto 2020 ^136^ | Pedometer | ActiGraph | Daily Minutes of Physical Activity | Continuous | On Treatment | 7 days ever 3 months for a year | Retrospective Monitoring | Not reported | Monitoring | Sedentary behavior |  |
| Pinto 2021 ^137^ | Pedometer | Fitbit, ActiGraph | Daily Step Count and Minutes of Physical Activity | Continuous | Post-Treatment | 12 weeks | Retrospective Monitoring | Not reported | Intervention | Physical activity |  |
| Pinto 2021 ^138^ | Pedometer | ActiGraph | Daily Minutes of Physical Activity | Continuous | On Treatment | 7 days every 3 months for a year | Retrospective Monitoring | Not reported | Intervention | Physical activity | >0.05 |
| Prinsen 2013 ^143^ | Pedometer | Not reported | Daily Minutes of Physical Activity | Discrete Timepoints | Post-Treatment | 12 days at 0 and 6 months | Retrospective Monitoring | Not reported | Monitoring | Maximal exercise performance | 0.011 (proportionally correlated) |
| Quintiliani 2016 ^144^ | Pedometer | Not reported | Daily Step Count and Minutes of Physical Activity | Continuous | Post-Treatment | 10 weeks | Retrospective Monitoring | Not reported | Intervention | Physical activity, self-efficacy |  |
| Rastogi 2020 ^146^ | Pedometer | Fitbit | Daily Minutes of Physical Activity | Discrete Timepoints | Post-Treatment | 12 weeks | Retrospective Monitoring | Not reported | Intervention | Quality of life, social support, sleep impairment | Quality of life - >0.05  Sleep impairment – 0.04 (inversely correlated)  Social support - >0.05 |
| Robertson 2019 ^149^ | Pedometer | ActiGraph | Daily Minutes of Physical Activity | Continuous | Post-Treatment | 7 days at 0 and 6 months | Retrospective Monitoring | Not reported | Intervention | Physical activity, quality of life |  |
| Rogers 2009 ^150^ | Pedometer | ActiGraph | Daily Step Count and Minutes of Physical Activity | Continuous | Post-Treatment | 7 days at 0, 3, and 6 months | Retrospective Monitoring | Not reported | Intervention | Physical activity | 0.012 (proportionally correlated) |
| Rogers 2009 ^151^ | Pedometer | ActiGraph | Daily Step Count and Minutes of Physical Activity | Continuous | Post-Treatment | 7 days at 0, and 3 months | Retrospective Monitoring | 99% | Intervention | Physical activity | 0.004 (proportionally correlated) |
| Rogers 2014 ^152^ | Pedometer | ActiGraph | Daily Minutes of Physical Activity | Continuous | Post-Treatment | 7 days at 0 and 3 months | Retrospective Monitoring | Not reported | Intervention | Fatigue |  |
| Rogers 2015 ^153^ | Pedometer | ActiGraph | Daily Minutes of Physical Activity | Continuous | Post-Treatment | 7 days at 0, 3 and 6 months | Retrospective Monitoring | Not reported | Intervention | Physical activity, aerobic fitness, quality of life | Physical activity – <0.01 (proportionally correlated)  Aerobic fitness – <0.01 (proportionally correlated)  Quality of life – 0.03 (proportionally correlated) |
| Rogers 2015 ^154^ | Pedometer | ActiGraph | Daily Minutes of Physical Activity | Continuous | Post-Treatment | 7 days at 0 and 3 months | Retrospective Monitoring | Not reported | Intervention | Sleep | 0.03 (inversely correlated) |
| Rogers 2017 ^155^ | Pedometer | ActiGraph | Daily Minutes of Physical Activity | Continuous | Post-Treatment | 6 months | Retrospective Monitoring | Not reported | Intervention | Self-efficacy, perceived barrier interference | Self-efficacy - <0.05 (proportionally correlated)  Perceived barrier interference - <0.01 (inversely correlated) |
| Roveda 2017 ^156^ | Pedometer; Heart Rate Monitor | Body-Media | Daily Minutes of Physical Activity | Continuous | Post-Treatment | 3 months | Retrospective Monitoring | Not reported | Intervention | Sleep | 0.04 (inversely correlated) |
| Sabiston 2014 ^157^ | Pedometer | ActiGraph | Daily Minutes of Physical Activity | Continuous | Post-Treatment | 7 days every 3 months for a year | Retrospective Monitoring | Not reported | Monitoring | Physical activity | >0.05 |
| Sabiston 2017 ^158^ | Pedometer | ActiGraph | Daily Minutes of Physical Activity | Continuous | Post-Treatment | 7 days at 0 and 3 months | Retrospective Monitoring | Not reported | Monitoring | Sedentary behavior |  |
| Sabiston 2018 ^159^ | Pedometer | ActiGraph | Daily Minutes of Physical Activity | Continuous | Post-Treatment | 7 days every 3 months for a year | Retrospective Monitoring | Not reported | Monitoring | C-reactive protein | 0.02 (inversely correlated) |
| Sabiston 2019 ^160^ | Pedometer | StepsCount | Daily Step Count | Continuous | Post-Treatment | 12 weeks | Retrospective Monitoring | Not reported | Intervention | Physical activity | >0.05 |
| Sada 2021 ^161^ | Pedometer | PAMSys | Daily Minutes of Physical Activity | Continuous | Post-Treatment | 48 hours | Retrospective Monitoring | Not reported | Monitoring | Sedentary behavior, physical activity | Sedentary behavior – <0.01 (inversely correlated)  Physical activity – <0.01 (proportionally correlated) |
| Schrier 2021 ^165^ | Pedometer | Fitbit | Daily Step Count | Continuous | Post-Treatment | 12 weeks | Retrospective Monitoring | Not reported | Intervention | Physical activity | <0.01 (proportionally correlated) |
| Servaes 2002 ^166^ | Pedometer | Not reported | Daily Minutes of Physical Activity | Continuous | Post-Treatment | 12 days | Retrospective Monitoring | Not reported | Monitoring | Fatigue | <0.01 (inversely correlated) |
| Short 2015 ^169^ | Pedometer | Not reported | Daily Step Count | Continuous | Post-Treatment | 3 days at 0 and 4 months | Retrospective Monitoring | Not reported | Intervention | Physical activity | >0.05 |
| Smith 2019 ^173^ | Pedometer | ActiGraph | Daily Minutes of Physical Activity | Continuous | Pre-Treatment; Post-Treatment | 7 days at 0 and 5 weeks, 6 and 12 months | Retrospective Monitoring | 40% | Monitoring | Physical activity | >0.05 |
| Stacey 2017 ^174^ | Pedometer | Not reported | Daily Step Count | Continuous | Post-Treatment | 7 days at 0, 8 and 20 weeks | Retrospective Monitoring | Not reported | Intervention | Physical activity | 0.05 (proportionally correlated) |
| Sweegers 2019 ^176^ | Pedometer | ActiGraph | Daily Minutes of Physical Activity | Continuous | Post-Treatment | 7 days | Retrospective Monitoring | Not reported | Monitoring | Sedentary time, physical activity |  |
| Thuman 2019 ^177^ | Pedometer | Microsoft | Daily Step Count | Continuous | Post-Treatment | 3 months | Retrospective Monitoring | Not reported | Monitoring | Sleep, physical activity, heart rate variability |  |
| Tonorezos 2013 ^179^ | Pedometer | BodyMedia | Daily Minutes of Physical Activity | Continuous | Post-Treatment | 7 days | Retrospective Monitoring | Not reported | Monitoring | Body fat, body mass index, waist circumference, insulin resistance, metabolic syndrome | Body fat - <0.01 (inversely correlated)  Other outcomes - >0.05 |
| Vallance 2007 ^184^ | Pedometer | New Lifestyles Inc | Daily Step Count | Continuous | Post-Treatment | 12 weeks | Retrospective Monitoring | Not reported | Intervention | Physical activity, health-related quality of life | Physical activity – 0.02 (proportionally correlated)  Health-related quality of life – <0.01 (proportionally correlated) |
| Vallance 2008 ^183^ | Pedometer | New Lifestyles Inc | Daily Step Count | Continuous | Post-Treatment | 7 days at 0, 3, 6 months | Retrospective Monitoring | Not reported | Intervention | Physical activity, health-related quality of life | Physical activity - >0.05  Health-related quality of life - >0.05 |
| Van Blarigan 2019 ^186^ | Pedometer | ActiGraph, Fitbit | Daily Minutes of Physical Activity | Continuous | Post-Treatment | 7 days at 0 and 12 weeks | Retrospective Monitoring | 93% | Intervention | Physical activity | >0.05 |
| van de Wiel 2021 ^190^ | Pedometer | ActiGraph | Daily Minutes of Physical Activity | Continuous | Post-Treatment | 7 days at 0 and 6 months | Retrospective Monitoring | Not reported | Intervention | Physical activity | >0.05 |
| Walsh 2021 ^195^ | Pedometer | Fitbit | Daily Step Count | Continuous | Post-Treatment | 12 weeks | Retrospective Monitoring | Not reported | Intervention | Physical activity |  |
| Wang 2021 ^196^ | Pedometer | New Lifestyles | Daily Step Count | Continuous | Post-Treatment | 7 days every 4 weeks for 16 weeks | Retrospective Monitoring | Not reported | Intervention | Physical activity, physical well-being | Physical activity – 0.05 (proportionally correlated)  Physical fitness – 0.02 (proportionally correlated) |
| Weiner 2019 ^198^ | Pedometer | ActiGraph, Fitbit | Daily Minutes of Physical Activity | Continuous | On Treatment | 7 days at 0 and 12 weeks | Retrospective Monitoring | Not reported | Intervention | Sedentary behavior | 0.02 (inversely correlated) |
| Welch 2019 ^199^ | Pedometer | ActiGraph | Daily Minutes of Physical Activity | Continuous | Post-Treatment | 7 days | Retrospective Monitoring | Not reported | Monitoring | Quality of life | <0.05 (proportionally correlated) |
| Wiestad 2020 ^200^ | Pedometer | BodyMedia Inc | Daily Minutes of Physical Activity | Continuous | On Treatment | 7 days at 0 and 6 months | Retrospective Monitoring | Not reported | Monitoring | Maximal oxygen uptake | 0.01 (proportionally correlated) |
| Wilson 2005 ^201^ | Pedometer | Not reported | Daily Step Count | Continuous | Post-Treatment | 8 weeks | Retrospective Monitoring | 100% | Intervention | Physical activity | <0.01 (proportionally correlated) |
| Xu 2021 ^206^ | Pedometer | Huami Technology Company | Daily Step Count | Continuous | Post-Treatment | 3 months | Retrospective Monitoring | Not reported | Intervention | Body composition, quality of life | Body composition - <0.05 (inversely correlated)  Quality of life - >0.05 |

**Appendix 1.** Search Strategies

Ovid MEDLINE(R) ALL 1946 to February 17, 2022

| **#** | **Searches** | **Results** | **Type** |  |  |  |
| --- | --- | --- | --- | --- | --- | --- |
|  | | | | | | |
| 1 | exp Neoplasms/ | 3626383 | Advanced |  |  |  |
| 2 | Survivors/ | 6656 | Advanced |  |  |  |
| 3 | neoplas*.mp,kw. | 3166126 | Advanced |  |  |  |
| 4 | paraneoplas*.mp,kw. | 15715 | Advanced |  |  |  |
| 5 | *.mp,kw. | 2062227 | Advanced |  |  |  |
| 6 | tumo?r*.mp,kw. | 2335732 | Advanced |  |  |  |
| 7 | onco*.mp,kw. | 601877 | Advanced |  |  |  |
| 8 | metast*.mp,kw. | 641735 | Advanced |  |  |  |
| 9 | malignan*.mp,kw. | 640717 | Advanced |  |  |  |
| 10 | aberrant crypt foci.mp,kw. | 1626 | Advanced |  |  |  |
| 11 | acanthoma*.mp,kw. | 815 | Advanced |  |  |  |
| 12 | acrospiroma*.mp,kw. | 597 | Advanced |  |  |  |
| 13 | adamantinom*.mp,kw. | 1183 | Advanced |  |  |  |
| 14 | adenocarc*.mp,kw. | 261647 | Advanced |  |  |  |
| 15 | adenofibrom*.mp,kw. | 2366 | Advanced |  |  |  |
| 16 | adenolymphom*.mp,kw. | 1847 | Advanced |  |  |  |
| 17 | adenomat*.mp,kw. | 24280 | Advanced |  |  |  |
| 18 | adenomyo*.mp,kw. | 4584 | Advanced |  |  |  |
| 19 | adenosarcom*.mp,kw. | 692 | Advanced |  |  |  |
| 20 | adenosquam*.mp,kw. | 4019 | Advanced |  |  |  |
| 21 | ameloblastom*.mp,kw. | 5000 | Advanced |  |  |  |
| 22 | androblastom*.mp,kw. | 332 | Advanced |  |  |  |
| 23 | angiofibrom*.mp,kw. | 2576 | Advanced |  |  |  |
| 24 | angiokeratom*.mp,kw. | 1268 | Advanced |  |  |  |
| 25 | angiolipom*.mp,kw. | 741 | Advanced |  |  |  |
| 26 | angioma*.mp,kw. | 13710 | Advanced |  |  |  |
| 27 | angiomyolipom*.mp,kw. | 4595 | Advanced |  |  |  |
| 28 | angiomyom*.mp,kw. | 654 | Advanced |  |  |  |
| 29 | angiosarcom*.mp,kw. | 6997 | Advanced |  |  |  |
| 30 | apudoma*.mp,kw. | 561 | Advanced |  |  |  |
| 31 | arrhenoblastom*.mp,kw. | 354 | Advanced |  |  |  |
| 32 | astrocytom*.mp,kw. | 23187 | Advanced |  |  |  |
| 33 | blastom*.mp,kw. | 11529 | Advanced |  |  |  |
| 34 | Bowen*.mp,kw. | 3709 | Advanced |  |  |  |
| 35 | Brenner*.mp,kw. | 1679 | Advanced |  |  |  |
| 36 | Buschke-Lowenstein*.mp,kw. | 336 | Advanced |  |  |  |
| 37 | carcin*.mp,kw. | 1127153 | Advanced |  |  |  |
| 38 | cementoma*.mp,kw. | 529 | Advanced |  |  |  |
| 39 | chemodectomas*.mp,kw. | 309 | Advanced |  |  |  |
| 40 | cholangiocarcin*.mp,kw. | 17482 | Advanced |  |  |  |
| 41 | chondroblastom*.mp,kw. | 1529 | Advanced |  |  |  |
| 42 | chondroma*.mp,kw. | 6898 | Advanced |  |  |  |
| 43 | chordoma*.mp,kw. | 5080 | Advanced |  |  |  |
| 44 | chondrosarcom*.mp,kw. | 10543 | Advanced |  |  |  |
| 45 | choriocarcin*.mp,kw. | 9824 | Advanced |  |  |  |
| 46 | craniopharyngioma*.mp,kw. | 6187 | Advanced |  |  |  |
| 47 | cystadenofibrom*.mp,kw. | 257 | Advanced |  |  |  |
| 48 | cystosarcom*.mp,kw. | 648 | Advanced |  |  |  |
| 49 | cytoma*.mp,kw. | 377 | Advanced |  |  |  |
| 50 | dermatofibrosarcom*.mp,kw. | 2334 | Advanced |  |  |  |
| 51 | desmoplas*.mp,kw. | 5384 | Advanced |  |  |  |
| 52 | dysgerminoma*.mp,kw. | 6228 | Advanced |  |  |  |
| 53 | DCIS.mp,kw. | 5528 | Advanced |  |  |  |
| 54 | DSRCT.mp,kw. | 382 | Advanced |  |  |  |
| 55 | ependymom*.mp,kw. | 7656 | Advanced |  |  |  |
| 56 | Ewing*.mp,kw. | 12226 | Advanced |  |  |  |
| 57 | fibroadenom*.mp,kw. | 4751 | Advanced |  |  |  |
| 58 | fibroepithelial*.mp,kw. | 1275 | Advanced |  |  |  |
| 59 | fibroma*.mp,kw. | 21008 | Advanced |  |  |  |
| 60 | fibrosarcom*.mp,kw. | 17649 | Advanced |  |  |  |
| 61 | FAMMM.mp,kw. | 84 | Advanced |  |  |  |
| 62 | gangliogliom*.mp,kw. | 1744 | Advanced |  |  |  |
| 63 | ganglioneurom*.mp,kw. | 3480 | Advanced |  |  |  |
| 64 | gastrinoma*.mp,kw. | 2027 | Advanced |  |  |  |
| 65 | germinoma*.mp,kw. | 4195 | Advanced |  |  |  |
| 66 | glioblastom*.mp,kw. | 49170 | Advanced |  |  |  |
| 67 | glioma*.mp,kw. | 73136 | Advanced |  |  |  |
| 68 | gliosarcom*.mp,kw. | 1346 | Advanced |  |  |  |
| 69 | glomus jugulare*.mp,kw. | 1455 | Advanced |  |  |  |
| 70 | glomus tympanicum*.mp,kw. | 299 | Advanced |  |  |  |
| 71 | glucagonoma*.mp,kw. | 1217 | Advanced |  |  |  |
| 72 | gonadoblastom*.mp,kw. | 891 | Advanced |  |  |  |
| 73 | GCTOB.mp,kw. | 10 | Advanced |  |  |  |
| 74 | GIST?.mp,kw. | 8961 | Advanced |  |  |  |
| 75 | hemangioendotheliom*.mp,kw. | 4849 | Advanced |  |  |  |
| 76 | hemangiom*.mp,kw. | 36730 | Advanced |  |  |  |
| 77 | hemangiopericytom*.mp,kw. | 4003 | Advanced |  |  |  |
| 78 | hemangiosarcom*.mp,kw. | 7896 | Advanced |  |  |  |
| 79 | hamartoblastom*.mp,kw. | 49 | Advanced |  |  |  |
| 80 | hepatoblastom*.mp,kw. | 4173 | Advanced |  |  |  |
| 81 | hepatoma*.mp,kw. | 29737 | Advanced |  |  |  |
| 82 | histiocytom*.mp,kw. | 9046 | Advanced |  |  |  |
| 83 | hodgkin*.mp,kw. | 96187 | Advanced |  |  |  |
| 84 | nonhodgkin*.mp,kw. | 136 | Advanced |  |  |  |
| 85 | (hutchinson* adj2 freckle*).mp,kw. | 803 | Advanced |  |  |  |
| 86 | HNPCC.mp,kw. | 2303 | Advanced |  |  |  |
| 87 | immunocytom*.mp,kw. | 611 | Advanced |  |  |  |
| 88 | incidentaloma?.mp,kw. | 2543 | Advanced |  |  |  |
| 89 | insulinoma*.mp,kw. | 8177 | Advanced |  |  |  |
| 90 | kasabach merrit*.mp,kw. | 890 | Advanced |  |  |  |
| 91 | leiomyoblastom*.mp,kw. | 402 | Advanced |  |  |  |
| 92 | leiomyom*.mp,kw. | 26260 | Advanced |  |  |  |
| 93 | leiomyosarcom*.mp,kw. | 13106 | Advanced |  |  |  |
| 94 | leukem*.mp,kw. | 339567 | Advanced |  |  |  |
| 95 | preleukem*.mp,kw. | 2164 | Advanced |  |  |  |
| 96 | leukoplak*.mp,kw. | 7195 | Advanced |  |  |  |
| 97 | li-fraumeni*.mp,kw. | 1580 | Advanced |  |  |  |
| 98 | lipoblastom*.mp,kw. | 546 | Advanced |  |  |  |
| 99 | lipoma*.mp,kw. | 20866 | Advanced |  |  |  |
| 100 | liposarcom*.mp,kw. | 8280 | Advanced |  |  |  |
| 101 | luteoma*.mp,kw. | 249 | Advanced |  |  |  |
| 102 | lymphangio*.mp,kw. | 17141 | Advanced |  |  |  |
| 103 | lymphoblastom*.mp,kw. | 378 | Advanced |  |  |  |
| 104 | lymphocytom*.mp,kw. | 359 | Advanced |  |  |  |
| 105 | lymphoma*.mp,kw. | 264553 | Advanced |  |  |  |
| 106 | lymphosarcom*.mp,kw. | 5222 | Advanced |  |  |  |
| 107 | lynch*.mp,kw. | 5059 | Advanced |  |  |  |
| 108 | macroglobulinem*.mp,kw. | 6539 | Advanced |  |  |  |
| 109 | m?croprolactinom*.mp,kw. | 892 | Advanced |  |  |  |
| 110 | mastocytom*.mp,kw. | 2132 | Advanced |  |  |  |
| 111 | mastocytos?s*.mp,kw. | 4783 | Advanced |  |  |  |
| 112 | medulloblastom*.mp,kw. | 10714 | Advanced |  |  |  |
| 113 | meigs*.mp,kw. | 940 | Advanced |  |  |  |
| 114 | melanoameloblastom*.mp,kw. | 11 | Advanced |  |  |  |
| 115 | melanoblastom*.mp,kw. | 493 | Advanced |  |  |  |
| 116 | melanocarcin*.mp,kw. | 105 | Advanced |  |  |  |
| 117 | melanoma*.mp,kw. | 147132 | Advanced |  |  |  |
| 118 | melanosis.mp,kw. | 4856 | Advanced |  |  |  |
| 119 | melanotic*.mp,kw. | 3464 | Advanced |  |  |  |
| 120 | meningiom*.mp,kw. | 27796 | Advanced |  |  |  |
| 121 | mesenchymom*.mp,kw. | 2131 | Advanced |  |  |  |
| 122 | mesoblast*.mp,kw. | 834 | Advanced |  |  |  |
| 123 | mesonephrom*.mp,kw. | 1175 | Advanced |  |  |  |
| 124 | mesotheliom*.mp,kw. | 20059 | Advanced |  |  |  |
| 125 | metaplas*.mp,kw. | 26207 | Advanced |  |  |  |
| 126 | micrometast*.mp,kw. | 7148 | Advanced |  |  |  |
| 127 | muir-torre*.mp,kw. | 517 | Advanced |  |  |  |
| 128 | myelolipom*.mp,kw. | 1232 | Advanced |  |  |  |
| 129 | myoepitheliom*.mp,kw. | 1503 | Advanced |  |  |  |
| 130 | myofibrom*.mp,kw. | 911 | Advanced |  |  |  |
| 131 | myeloma*.mp,kw. | 67118 | Advanced |  |  |  |
| 132 | myoma*.mp,kw. | 6988 | Advanced |  |  |  |
| 133 | myosarcom*.mp,kw. | 415 | Advanced |  |  |  |
| 134 | myxofibrosarcom*.mp,kw. | 646 | Advanced |  |  |  |
| 135 | myxoma*.mp,kw. | 11383 | Advanced |  |  |  |
| 136 | myxosarcom*.mp,kw. | 504 | Advanced |  |  |  |
| 137 | n?evocarcin*.mp,kw. | 83 | Advanced |  |  |  |
| 138 | neurilemmom*.mp,kw. | 14563 | Advanced |  |  |  |
| 139 | neurocytom*.mp,kw. | 942 | Advanced |  |  |  |
| 140 | neuroectodermal*.mp,kw. | 8975 | Advanced |  |  |  |
| 141 | neurofibroma*.mp,kw. | 23284 | Advanced |  |  |  |
| 142 | neurofibrosarcom*.mp,kw. | 668 | Advanced |  |  |  |
| 143 | neurilemmom*.mp,kw. | 14563 | Advanced |  |  |  |
| 144 | neuroblastom*.mp,kw. | 45019 | Advanced |  |  |  |
| 145 | neuroma*.mp,kw. | 16215 | Advanced |  |  |  |
| 146 | neurothekeom*.mp,kw. | 307 | Advanced |  |  |  |
| 147 | NSCLC.mp,kw. | 52508 | Advanced |  |  |  |
| 148 | odontoma*.mp,kw. | 1723 | Advanced |  |  |  |
| 149 | oligo*.mp,kw. | 450903 | Advanced |  |  |  |
| 150 | osteoblastom*.mp,kw. | 1393 | Advanced |  |  |  |
| 151 | osteochondrom*.mp,kw. | 3780 | Advanced |  |  |  |
| 152 | osteoclastom*.mp,kw. | 384 | Advanced |  |  |  |
| 153 | osteoma*.mp,kw. | 15062 | Advanced |  |  |  |
| 154 | osteosarcom*.mp,kw. | 34529 | Advanced |  |  |  |
| 155 | papilloma*.mp,kw. | 79086 | Advanced |  |  |  |
| 156 | papillary*.mp,kw. | 73656 | Advanced |  |  |  |
| 157 | paragangliom*.mp,kw. | 10046 | Advanced |  |  |  |
| 158 | pheochromocytom*.mp,kw. | 23632 | Advanced |  |  |  |
| 159 | phyllo?des*.mp,kw. | 2832 | Advanced |  |  |  |
| 160 | pinealocytoma*.mp,kw. | 17 | Advanced |  |  |  |
| 161 | pinealoma*.mp,kw. | 2005 | Advanced |  |  |  |
| 162 | pineoblastoma*.mp,kw. | 461 | Advanced |  |  |  |
| 163 | pineocytoma*.mp,kw. | 273 | Advanced |  |  |  |
| 164 | plasmacytom*.mp,kw. | 11372 | Advanced |  |  |  |
| 165 | (polycythem* adj2 vera?).mp,kw. | 8326 | Advanced |  |  |  |
| 166 | prolactinom*.mp,kw. | 4668 | Advanced |  |  |  |
| 167 | retinoblastom*.mp,kw. | 23696 | Advanced |  |  |  |
| 168 | rhabdoid*.mp,kw. | 3161 | Advanced |  |  |  |
| 169 | rhabdomyom*.mp,kw. | 1929 | Advanced |  |  |  |
| 170 | rhabdomyosarcom*.mp,kw. | 15646 | Advanced |  |  |  |
| 171 | sarcom*.mp,kw. | 142716 | Advanced |  |  |  |
| 172 | seminoma*.mp,kw. | 8269 | Advanced |  |  |  |
| 173 | Sertoli- Leydig.mp,kw. | 1285 | Advanced |  |  |  |
| 174 | somatostatinoma*.mp,kw. | 495 | Advanced |  |  |  |
| 175 | somatotrophinom*.mp,kw. | 82 | Advanced |  |  |  |
| 176 | struma ovarii*.mp,kw. | 750 | Advanced |  |  |  |
| 177 | thecoma*.mp,kw. | 1112 | Advanced |  |  |  |
| 178 | teratocarcin*.mp,kw. | 2935 | Advanced |  |  |  |
| 179 | teratoma*.mp,kw. | 23815 | Advanced |  |  |  |
| 180 | thymom*.mp,kw. | 12279 | Advanced |  |  |  |
| 181 | trophoblast*.mp,kw. | 27757 | Advanced |  |  |  |
| 182 | vipoma*.mp,kw. | 659 | Advanced |  |  |  |
| 183 | wilms*.mp,kw. | 13849 | Advanced |  |  |  |
| 184 | or/1-183 | 5722168 | Advanced |  |  |  |
| 185 | Wearable Electronic Devices/ | 5536 | Advanced |  |  |  |
| 186 | Accelerometry/ | 6836 | Advanced |  |  |  |
| 187 | Actigraphy/ | 4394 | Advanced |  |  |  |
| 188 | Fitness Trackers/ | 975 | Advanced |  |  |  |
| 189 | Remote Sensing Technology/ | 3577 | Advanced |  |  |  |
| 190 | Smart Glasses/ | 117 | Advanced |  |  |  |
| 191 | wearable?.mp,kw. | 20490 | Advanced |  |  |  |
| 192 | wear-able?.mp,kw. | 3 | Advanced |  |  |  |
| 193 | acceleromet?r*.mp,kw. | 21456 | Advanced |  |  |  |
| 194 | actigraph*.mp,kw. | 9908 | Advanced |  |  |  |
| 195 | (activit* adj3 mobile?).mp,kw. | 418 | Advanced |  |  |  |
| 196 | (activit* adj3 track*).mp,kw. | 2571 | Advanced |  |  |  |
| 197 | (activit* adj3 monitor*).mp,kw. | 19623 | Advanced |  |  |  |
| 198 | (fitness* adj3 mobile?).mp,kw. | 42 | Advanced |  |  |  |
| 199 | (fitness* adj3 band?).mp,kw. | 22 | Advanced |  |  |  |
| 200 | (fitness* adj3 device?).mp,kw. | 117 | Advanced |  |  |  |
| 201 | (fitness* adj3 track*).mp,kw. | 1370 | Advanced |  |  |  |
| 202 | (fitness* adj3 monitor*).mp,kw. | 313 | Advanced |  |  |  |
| 203 | (move* adj3 mobile?).mp,kw. | 392 | Advanced |  |  |  |
| 204 | movemonitor*.mp,kw. | 22 | Advanced |  |  |  |
| 205 | move-monitor*.mp,kw. | 4 | Advanced |  |  |  |
| 206 | (physical* adj3 mobile?).mp,kw. | 185 | Advanced |  |  |  |
| 207 | (physical* adj3 track*).mp,kw. | 670 | Advanced |  |  |  |
| 208 | ((selfmonitor* or self-monitor*) adj3 mobile?).mp,kw. | 80 | Advanced |  |  |  |
| 209 | ((selfmonitor* or self-monitor*) adj3 device?).mp,kw. | 185 | Advanced |  |  |  |
| 210 | selftrack*.mp,kw. | 1 | Advanced |  |  |  |
| 211 | (self adj3 track*).mp,kw. | 692 | Advanced |  |  |  |
| 212 | ((wellness* or well-ness*) adj3 mobile?).mp,kw. | 40 | Advanced |  |  |  |
| 213 | ((wellness* or well-ness*) adj3 device?).mp,kw. | 19 | Advanced |  |  |  |
| 214 | ((wellness* or well-ness*) adj3 track*).mp,kw. | 28 | Advanced |  |  |  |
| 215 | mtrack*.mp,kw. | 7 | Advanced |  |  |  |
| 216 | m-track*.mp,kw. | 128 | Advanced |  |  |  |
| 217 | etrack*.mp,kw. | 19 | Advanced |  |  |  |
| 218 | e-track*.mp,kw. | 30 | Advanced |  |  |  |
| 219 | ((biometric? or bio-metric?) adj3 mobile?).mp,kw. | 20 | Advanced |  |  |  |
| 220 | ((biometric? or bio-metric?) adj3 monitor*).mp,kw. | 90 | Advanced |  |  |  |
| 221 | ((biometric? or bio-metric?) adj3 sensor?).mp,kw. | 74 | Advanced |  |  |  |
| 222 | (remote* adj3 sensing).mp,kw. | 12667 | Advanced |  |  |  |
| 223 | (embedded adj3 sensor?).mp,kw. | 1182 | Advanced |  |  |  |
| 224 | (movement? adj3 sensor?).mp,kw. | 1523 | Advanced |  |  |  |
| 225 | (ehealth* adj3 mobile?).mp,kw. | 118 | Advanced |  |  |  |
| 226 | (phealth* adj3 mobile?).mp,kw. | 0 | Advanced |  |  |  |
| 227 | (uhealth* adj3 mobile?).mp,kw. | 1 | Advanced |  |  |  |
| 228 | (smart* adj3 device?).mp,kw. | 3184 | Advanced |  |  |  |
| 229 | (smart* adj3 technolog*).mp,kw. | 2681 | Advanced |  |  |  |
| 230 | (smart* adj3 wireless*).mp,kw. | 428 | Advanced |  |  |  |
| 231 | (smart* adj3 sensor?).mp,kw. | 1923 | Advanced |  |  |  |
| 232 | (smart* adj3 biosensor?).mp,kw. | 235 | Advanced |  |  |  |
| 233 | (smart* adj3 monitor*).mp,kw. | 1121 | Advanced |  |  |  |
| 234 | (smart* adj3 track*).mp,kw. | 307 | Advanced |  |  |  |
| 235 | (smart* adj3 health*).mp,kw. | 1933 | Advanced |  |  |  |
| 236 | (smart* adj3 ehealth*).mp,kw. | 160 | Advanced |  |  |  |
| 237 | (smart* adj3 mhealth*).mp,kw. | 707 | Advanced |  |  |  |
| 238 | (smart* adj3 phealth*).mp,kw. | 1 | Advanced |  |  |  |
| 239 | (smart* adj3 uhealth*).mp,kw. | 1 | Advanced |  |  |  |
| 240 | (smart* adj3 (count or counts or counter?)).mp,kw. | 45 | Advanced |  |  |  |
| 241 | (smart* adj3 logger?).mp,kw. | 6 | Advanced |  |  |  |
| 242 | (smart* adj3 wear*).mp,kw. | 1317 | Advanced |  |  |  |
| 243 | (smart* adj3 worn*).mp,kw. | 58 | Advanced |  |  |  |
| 244 | smartband?.mp,kw. | 18 | Advanced |  |  |  |
| 245 | (smart* adj3 band?).mp,kw. | 65 | Advanced |  |  |  |
| 246 | (smart* adj3 armband?).mp,kw. | 5 | Advanced |  |  |  |
| 247 | smartglass*.mp,kw. | 31 | Advanced |  |  |  |
| 248 | (smart* adj3 glass*).mp,kw. | 288 | Advanced |  |  |  |
| 249 | (smart* adj3 (eyewear? or eye-wear?)).mp,kw. | 3 | Advanced |  |  |  |
| 250 | smartring?.mp,kw. | 0 | Advanced |  |  |  |
| 251 | (smart* adj3 ring?).mp,kw. | 24 | Advanced |  |  |  |
| 252 | smartwatch*.mp,kw. | 623 | Advanced |  |  |  |
| 253 | (smart* adj3 watch*).mp,kw. | 236 | Advanced |  |  |  |
| 254 | (smart* adj3 cloth*).mp,kw. | 139 | Advanced |  |  |  |
| 255 | (smart* adj3 fabric?).mp,kw. | 101 | Advanced |  |  |  |
| 256 | (smart* adj3 apparel?).mp,kw. | 5 | Advanced |  |  |  |
| 257 | (smart* adj3 garment?).mp,kw. | 96 | Advanced |  |  |  |
| 258 | (electronic* adj3 textile?).mp,kw. | 424 | Advanced |  |  |  |
| 259 | (e-textile? or etextile?).mp,kw. | 220 | Advanced |  |  |  |
| 260 | (smart* adj3 textile?).mp,kw. | 445 | Advanced |  |  |  |
| 261 | (smart* adj3 cap?).mp,kw. | 12 | Advanced |  |  |  |
| 262 | (smart* adj3 dress*).mp,kw. | 79 | Advanced |  |  |  |
| 263 | (smart* adj3 glove?).mp,kw. | 47 | Advanced |  |  |  |
| 264 | (smart* adj3 hat?).mp,kw. | 6 | Advanced |  |  |  |
| 265 | (smart* adj3 helmet?).mp,kw. | 7 | Advanced |  |  |  |
| 266 | (smart* adj3 jacket?).mp,kw. | 4 | Advanced |  |  |  |
| 267 | (smart* adj3 insoles*).mp,kw. | 16 | Advanced |  |  |  |
| 268 | (smart* adj3 pant?).mp,kw. | 2 | Advanced |  |  |  |
| 269 | (smart* adj3 scarf?).mp,kw. | 0 | Advanced |  |  |  |
| 270 | (smart* adj3 shirt?).mp,kw. | 36 | Advanced |  |  |  |
| 271 | (smart* adj3 footwear?).mp,kw. | 3 | Advanced |  |  |  |
| 272 | (smart* adj3 shoe?).mp,kw. | 42 | Advanced |  |  |  |
| 273 | (smart* adj3 sock?).mp,kw. | 22 | Advanced |  |  |  |
| 274 | (smart* adj3 strap?).mp,kw. | 3 | Advanced |  |  |  |
| 275 | (smart* adj3 suit?).mp,kw. | 25 | Advanced |  |  |  |
| 276 | (smart* adj3 vest?).mp,kw. | 7 | Advanced |  |  |  |
| 277 | (smart* adj3 societ*).mp,kw. | 40 | Advanced |  |  |  |
| 278 | (intelligent adj3 sensor?).mp,kw. | 373 | Advanced |  |  |  |
| 279 | (intelligen* adj3 biosensor?).mp,kw. | 43 | Advanced |  |  |  |
| 280 | (intelligen* adj3 wear*).mp,kw. | 179 | Advanced |  |  |  |
| 281 | (intelligen* adj3 worn*).mp,kw. | 2 | Advanced |  |  |  |
| 282 | (intelligen* adj3 band?).mp,kw. | 12 | Advanced |  |  |  |
| 283 | (intelligen* adj3 armband?).mp,kw. | 1 | Advanced |  |  |  |
| 284 | (intelligen* adj3 glass*).mp,kw. | 11 | Advanced |  |  |  |
| 285 | (intelligen* adj3 (eyewear? or eye-wear?)).mp,kw. | 0 | Advanced |  |  |  |
| 286 | (intelligen* adj3 ring?).mp,kw. | 1 | Advanced |  |  |  |
| 287 | (intelligen* adj3 watch*).mp,kw. | 9 | Advanced |  |  |  |
| 288 | (intelligen* adj3 cloth*).mp,kw. | 26 | Advanced |  |  |  |
| 289 | (intelligen* adj3 fabric?).mp,kw. | 15 | Advanced |  |  |  |
| 290 | (intelligen* adj3 apparel?).mp,kw. | 2 | Advanced |  |  |  |
| 291 | (intelligen* adj3 garment?).mp,kw. | 11 | Advanced |  |  |  |
| 292 | (intelligen* adj3 textile?).mp,kw. | 38 | Advanced |  |  |  |
| 293 | (intelligen* adj3 belt?).mp,kw. | 4 | Advanced |  |  |  |
| 294 | (intelligen* adj3 cap?).mp,kw. | 2 | Advanced |  |  |  |
| 295 | (intelligen* adj3 dress*).mp,kw. | 17 | Advanced |  |  |  |
| 296 | (intelligen* adj3 glove?).mp,kw. | 7 | Advanced |  |  |  |
| 297 | (intelligen* adj3 hat?).mp,kw. | 1 | Advanced |  |  |  |
| 298 | (intelligen* adj3 helmet?).mp,kw. | 0 | Advanced |  |  |  |
| 299 | (intelligen* adj3 jacket?).mp,kw. | 0 | Advanced |  |  |  |
| 300 | (intelligen* adj3 insoles*).mp,kw. | 4 | Advanced |  |  |  |
| 301 | (intelligen* adj3 pant?).mp,kw. | 0 | Advanced |  |  |  |
| 302 | (intelligen* adj3 scarf?).mp,kw. | 0 | Advanced |  |  |  |
| 303 | (intelligen* adj3 shirt?).mp,kw. | 0 | Advanced |  |  |  |
| 304 | (intelligen* adj3 footwear?).mp,kw. | 3 | Advanced |  |  |  |
| 305 | (intelligen* adj3 shoe?).mp,kw. | 5 | Advanced |  |  |  |
| 306 | (intelligen* adj3 sock?).mp,kw. | 0 | Advanced |  |  |  |
| 307 | (intelligen* adj3 strap?).mp,kw. | 0 | Advanced |  |  |  |
| 308 | (intelligen* adj3 suit?).mp,kw. | 8 | Advanced |  |  |  |
| 309 | (intelligen* adj3 vest?).mp,kw. | 1 | Advanced |  |  |  |
| 310 | I-wear*.mp,kw. | 44 | Advanced |  |  |  |
| 311 | (ambient* adj3 intelligen*).mp,kw. | 206 | Advanced |  |  |  |
| 312 | (ambient* adj3 wear*).mp,kw. | 72 | Advanced |  |  |  |
| 313 | (ambient* adj3 worn*).mp,kw. | 6 | Advanced |  |  |  |
| 314 | sensor?-wear*.mp,kw. | 122 | Advanced |  |  |  |
| 315 | sens*wear*.mp,kw. | 540 | Advanced |  |  |  |
| 316 | (wireless* adj3 wear*).mp,kw. | 623 | Advanced |  |  |  |
| 317 | (wireless* adj3 worn*).mp,kw. | 55 | Advanced |  |  |  |
| 318 | (wireless* adj3 biosensor*).mp,kw. | 92 | Advanced |  |  |  |
| 319 | (wireless* adj3 textile?).mp,kw. | 13 | Advanced |  |  |  |
| 320 | (wireless body adj3 area network?).mp,kw. | 340 | Advanced |  |  |  |
| 321 | (wireless sensor? adj3 network-based).mp,kw. | 36 | Advanced |  |  |  |
| 322 | WBAN?.mp,kw. | 281 | Advanced |  |  |  |
| 323 | ankle?-worn*.mp,kw. | 49 | Advanced |  |  |  |
| 324 | ankle?-mount*.mp,kw. | 31 | Advanced |  |  |  |
| 325 | finger?-worn*.mp,kw. | 16 | Advanced |  |  |  |
| 326 | waist?-worn*.mp,kw. | 192 | Advanced |  |  |  |
| 327 | waist?-mount*.mp,kw. | 104 | Advanced |  |  |  |
| 328 | wrist?-worn*.mp,kw. | 1211 | Advanced |  |  |  |
| 329 | wrist?-mount*.mp,kw. | 85 | Advanced |  |  |  |
| 330 | pedometer?.mp,kw. | 2932 | Advanced |  |  |  |
| 331 | step?count*.mp,kw. | 191 | Advanced |  |  |  |
| 332 | (step? adj3 count*).mp,kw. | 3211 | Advanced |  |  |  |
| 333 | (adidas adj3 watch*).mp,kw. | 0 | Advanced |  |  |  |
| 334 | (apple adj3 watch*).mp,kw. | 212 | Advanced |  |  |  |
| 335 | (boat adj3 watch*).mp,kw. | 6 | Advanced |  |  |  |
| 336 | (casio adj3 watch*).mp,kw. | 3 | Advanced |  |  |  |
| 337 | amazfit?.mp,kw. | 5 | Advanced |  |  |  |
| 338 | fitbit?.mp,kw. | 926 | Advanced |  |  |  |
| 339 | (fossil adj3 watch*).mp,kw. | 1 | Advanced |  |  |  |
| 340 | garmin?.mp,kw. | 212 | Advanced |  |  |  |
| 341 | (hexoskin adj3 watch*).mp,kw. | 0 | Advanced |  |  |  |
| 342 | (huawei adj3 watch*).mp,kw. | 6 | Advanced |  |  |  |
| 343 | (jawbone adj3 watch*).mp,kw. | 0 | Advanced |  |  |  |
| 344 | (lg adj3 watch*).mp,kw. | 1 | Advanced |  |  |  |
| 345 | misfit?.mp,kw. | 1937 | Advanced |  |  |  |
| 346 | (mi adj3 watch*).mp,kw. | 5 | Advanced |  |  |  |
| 347 | (nike adj3 watch*).mp,kw. | 1 | Advanced |  |  |  |
| 348 | (puma adj3 watch*).mp,kw. | 0 | Advanced |  |  |  |
| 349 | (samsung adj3 watch*).mp,kw. | 8 | Advanced |  |  |  |
| 350 | (xiaomi adj3 watch*).mp,kw. | 0 | Advanced |  |  |  |
| 351 | whoop.mp,kw. | 97 | Advanced |  |  |  |
| 352 | (consumer? adj5 (base? or level? or grade?) adj5 device?).mp,kw. | 115 | Advanced |  |  |  |
| 353 | (consumer? adj5 (base? or level? or grade?) adj5 measur*).mp,kw. | 80 | Advanced |  |  |  |
| 354 | (consumer? adj5 (base? or level? or grade?) adj5 monitor*).mp,kw. | 85 | Advanced |  |  |  |
| 355 | (consumer? adj5 (base? or level? or grade?) adj5 tracker?).mp,kw. | 48 | Advanced |  |  |  |
| 356 | (consumer? adj5 activit* adj5 device?).mp,kw. | 17 | Advanced |  |  |  |
| 357 | (consumer? adj5 activit* adj5 measur*).mp,kw. | 22 | Advanced |  |  |  |
| 358 | (consumer? adj5 activit* adj5 monitor*).mp,kw. | 63 | Advanced |  |  |  |
| 359 | (consumer? adj5 activit* adj5 tracker?).mp,kw. | 60 | Advanced |  |  |  |
| 360 | (consumer? adj5 PAM?).mp,kw. | 7 | Advanced |  |  |  |
| 361 | (consumer? adj5 IMU?).mp,kw. | 5 | Advanced |  |  |  |
| 362 | or/185-361 | 99373 | Advanced |  |  |  |
| 363 | 184 and 362 | 5024 | Advanced |  |  |  |
| 364 | ((exp animals/ or exp animal experiment/) not humans/) or (in vitro or in vivo or cell lines).ti. | 5268152 | Advanced |  |  |  |
| 365 | 363 not 364 | 4396 | Advanced |  |  |  |
| 366 | clinical trials data monitoring committees/ | 543 | Advanced |  |  |  |
| 367 | clinical study/ | 4387 | Advanced |  |  |  |
| 368 | clinical studies as topic/ | 703 | Advanced |  |  |  |
| 369 | clinical trial protocol/ | 7437 | Advanced |  |  |  |
| 370 | clinical trial protocols as topic/ | 105 | Advanced |  |  |  |
| 371 | exp clinical trial/ | 929100 | Advanced |  |  |  |
| 372 | exp controlled clinical trial/ | 650163 | Advanced |  |  |  |
| 373 | exp clinical trials as topic/ | 370543 | Advanced |  |  |  |
| 374 | exp controlled clinical trials as topic/ | 162272 | Advanced |  |  |  |
| 375 | exp randomized controlled trial/ | 560349 | Advanced |  |  |  |
| 376 | exp randomized controlled trials as topic/ | 156616 | Advanced |  |  |  |
| 377 | adaptive clinical trial/ | 36 | Advanced |  |  |  |
| 378 | adaptive clinical trials as topic/ | 81 | Advanced |  |  |  |
| 379 | equivalence trial/ | 981 | Advanced |  |  |  |
| 380 | equivalence trials as topic/ | 576 | Advanced |  |  |  |
| 381 | pragmatic clinical trial/ | 2033 | Advanced |  |  |  |
| 382 | pragmatic clinical trials as topic/ | 730 | Advanced |  |  |  |
| 383 | multicenter studies/ | 315475 | Advanced |  |  |  |
| 384 | multicenter studies as topic/ | 20745 | Advanced |  |  |  |
| 385 | comparative study/ | 1908889 | Advanced |  |  |  |
| 386 | feasibility studies/ | 78194 | Advanced |  |  |  |
| 387 | control groups/ | 1814 | Advanced |  |  |  |
| 388 | random allocation/ | 106612 | Advanced |  |  |  |
| 389 | single-blind method/ | 31607 | Advanced |  |  |  |
| 390 | double-blind method/ | 170100 | Advanced |  |  |  |
| 391 | placebos/ | 35886 | Advanced |  |  |  |
| 392 | clinical study.pt. | 4387 | Advanced |  |  |  |
| 393 | clinical trial.pt. | 533886 | Advanced |  |  |  |
| 394 | clinical trial protocol.pt. | 7437 | Advanced |  |  |  |
| 395 | clinical trial protocols as topic.pt. | 0 | Advanced |  |  |  |
| 396 | clinical trial, phase i.pt. | 23236 | Advanced |  |  |  |
| 397 | clinical trial, phase ii.pt. | 37100 | Advanced |  |  |  |
| 398 | clinical trial, phase iii.pt. | 19963 | Advanced |  |  |  |
| 399 | clinical trial, phase iv.pt. | 2278 | Advanced |  |  |  |
| 400 | controlled clinical trial.pt. | 94700 | Advanced |  |  |  |
| 401 | randomized controlled trial.pt. | 558953 | Advanced |  |  |  |
| 402 | adaptive clinical trial.pt. | 36 | Advanced |  |  |  |
| 403 | equivalence trial.pt. | 981 | Advanced |  |  |  |
| 404 | pragmatic clinical trial.pt. | 2033 | Advanced |  |  |  |
| 405 | multicenter study.pt. | 315475 | Advanced |  |  |  |
| 406 | comparative study.pt. | 1908889 | Advanced |  |  |  |
| 407 | random*.mp,kw. | 1538538 | Advanced |  |  |  |
| 408 | nonrandom*.mp,kw. | 21323 | Advanced |  |  |  |
| 409 | quasirandom*.mp,kw. | 194 | Advanced |  |  |  |
| 410 | ((quasiexperimental or quasi-experimental) adj4 (study or studies)).mp,kw. | 9995 | Advanced |  |  |  |
| 411 | (control* adj4 (study or studies or group*)).mp,kw. | 1104753 | Advanced |  |  |  |
| 412 | (allocate* adj4 (study or studies or group*)).mp,kw. | 23127 | Advanced |  |  |  |
| 413 | (pragmatic* adj4 (study or studies)).mp,kw. | 2108 | Advanced |  |  |  |
| 414 | (equivalence adj4 (study or studies)).mp,kw. | 1086 | Advanced |  |  |  |
| 415 | (superiority adj4 (study or studies)).mp,kw. | 1917 | Advanced |  |  |  |
| 416 | ((noninferiority or non-inferiority) adj4 (study or studies)).mp,kw. | 1927 | Advanced |  |  |  |
| 417 | (pivotal* adj4 (study or studies)).mp,kw. | 2984 | Advanced |  |  |  |
| 418 | (comparative adj4 (study or studies)).mp,kw. | 1989598 | Advanced |  |  |  |
| 419 | (comparative adj2 effective*).mp,kw. | 11808 | Advanced |  |  |  |
| 420 | ((open label or open-label) adj4 (study or studies)).mp,kw. | 24047 | Advanced |  |  |  |
| 421 | (closed label or closed-label).mp,kw. | 16 | Advanced |  |  |  |
| 422 | (conceal* adj2 allocat*).mp,kw. | 3178 | Advanced |  |  |  |
| 423 | (intention* adj2 treat* adj2 analys?s*).mp,kw. | 12828 | Advanced |  |  |  |
| 424 | feasibility.mp,kw. | 237123 | Advanced |  |  |  |
| 425 | trial?.mp,kw. | 1886393 | Advanced |  |  |  |
| 426 | phase?.mp,kw. | 1247366 | Advanced |  |  |  |
| 427 | multicent?r*.mp,kw. | 400513 | Advanced |  |  |  |
| 428 | multi-cent?r*.mp,kw. | 23402 | Advanced |  |  |  |
| 429 | "single blind*".mp,kw. | 40771 | Advanced |  |  |  |
| 430 | "single mask*".mp,kw. | 552 | Advanced |  |  |  |
| 431 | "single dumm*".mp,kw. | 14 | Advanced |  |  |  |
| 432 | "double blind*".mp,kw. | 215167 | Advanced |  |  |  |
| 433 | "double mask*".mp,kw. | 3504 | Advanced |  |  |  |
| 434 | "double dumm*".mp,kw. | 2396 | Advanced |  |  |  |
| 435 | "triple-blind*".mp,kw. | 1213 | Advanced |  |  |  |
| 436 | "triple mask*".mp,kw. | 73 | Advanced |  |  |  |
| 437 | "triple dumm*".mp,kw. | 43 | Advanced |  |  |  |
| 438 | "treble blind*".mp,kw. | 0 | Advanced |  |  |  |
| 439 | "treble mask*".mp,kw. | 0 | Advanced |  |  |  |
| 440 | "treble dumm*".mp,kw. | 0 | Advanced |  |  |  |
| 441 | placebo*.mp,kw. | 247551 | Advanced |  |  |  |
| 442 | sham.mp,kw. | 93925 | Advanced |  |  |  |
| 443 | or/366-442 | 6156283 | Advanced |  |  |  |
| 444 | 365 and 443 | 1577 | Advanced |  |  |  |

Ovid Cochrane Central Register of Controlled Trials January 2022

|  |  |  |  |  |  |  |
| --- | --- | --- | --- | --- | --- | --- |
| **#** | **Searches** | **Results** | **Type** |  |  |  |
|  | | | | | | |
| 1 | exp Neoplasms/ | 85828 | Advanced |  |  |  |
| 2 | Survivors/ | 463 | Advanced |  |  |  |
| 3 | neoplas*.mp,kw. | 91595 | Advanced |  |  |  |
| 4 | paraneoplas*.mp,kw. | 113 | Advanced |  |  |  |
| 5 | *.mp,kw. | 193478 | Advanced |  |  |  |
| 6 | tumo?r*.mp,kw. | 88627 | Advanced |  |  |  |
| 7 | onco*.mp,kw. | 37483 | Advanced |  |  |  |
| 8 | metast*.mp,kw. | 50542 | Advanced |  |  |  |
| 9 | malignan*.mp,kw. | 31825 | Advanced |  |  |  |
| 10 | aberrant crypt foci.mp,kw. | 52 | Advanced |  |  |  |
| 11 | acanthoma*.mp,kw. | 3 | Advanced |  |  |  |
| 12 | acrospiroma*.mp,kw. | 0 | Advanced |  |  |  |
| 13 | adamantinom*.mp,kw. | 13 | Advanced |  |  |  |
| 14 | adenocarc*.mp,kw. | 12611 | Advanced |  |  |  |
| 15 | adenofibrom*.mp,kw. | 3 | Advanced |  |  |  |
| 16 | adenolymphom*.mp,kw. | 7 | Advanced |  |  |  |
| 17 | adenomat*.mp,kw. | 907 | Advanced |  |  |  |
| 18 | adenomyo*.mp,kw. | 339 | Advanced |  |  |  |
| 19 | adenosarcom*.mp,kw. | 8 | Advanced |  |  |  |
| 20 | adenosquam*.mp,kw. | 451 | Advanced |  |  |  |
| 21 | ameloblastom*.mp,kw. | 12 | Advanced |  |  |  |
| 22 | androblastom*.mp,kw. | 3 | Advanced |  |  |  |
| 23 | angiofibrom*.mp,kw. | 42 | Advanced |  |  |  |
| 24 | angiokeratom*.mp,kw. | 14 | Advanced |  |  |  |
| 25 | angiolipom*.mp,kw. | 3 | Advanced |  |  |  |
| 26 | angioma*.mp,kw. | 162 | Advanced |  |  |  |
| 27 | angiomyolipom*.mp,kw. | 101 | Advanced |  |  |  |
| 28 | angiomyom*.mp,kw. | 0 | Advanced |  |  |  |
| 29 | angiosarcom*.mp,kw. | 106 | Advanced |  |  |  |
| 30 | apudoma*.mp,kw. | 0 | Advanced |  |  |  |
| 31 | arrhenoblastom*.mp,kw. | 0 | Advanced |  |  |  |
| 32 | astrocytom*.mp,kw. | 663 | Advanced |  |  |  |
| 33 | blastom*.mp,kw. | 249 | Advanced |  |  |  |
| 34 | Bowen*.mp,kw. | 185 | Advanced |  |  |  |
| 35 | Brenner*.mp,kw. | 49 | Advanced |  |  |  |
| 36 | Buschke-Lowenstein*.mp,kw. | 0 | Advanced |  |  |  |
| 37 | carcin*.mp,kw. | 50863 | Advanced |  |  |  |
| 38 | cementoma*.mp,kw. | 0 | Advanced |  |  |  |
| 39 | chemodectomas*.mp,kw. | 0 | Advanced |  |  |  |
| 40 | cholangiocarcin*.mp,kw. | 898 | Advanced |  |  |  |
| 41 | chondroblastom*.mp,kw. | 1 | Advanced |  |  |  |
| 42 | chondroma*.mp,kw. | 110 | Advanced |  |  |  |
| 43 | chordoma*.mp,kw. | 79 | Advanced |  |  |  |
| 44 | chondrosarcom*.mp,kw. | 118 | Advanced |  |  |  |
| 45 | choriocarcin*.mp,kw. | 46 | Advanced |  |  |  |
| 46 | craniopharyngioma*.mp,kw. | 111 | Advanced |  |  |  |
| 47 | cystadenofibrom*.mp,kw. | 0 | Advanced |  |  |  |
| 48 | cystosarcom*.mp,kw. | 9 | Advanced |  |  |  |
| 49 | cytoma*.mp,kw. | 7 | Advanced |  |  |  |
| 50 | dermatofibrosarcom*.mp,kw. | 21 | Advanced |  |  |  |
| 51 | desmoplas*.mp,kw. | 93 | Advanced |  |  |  |
| 52 | dysgerminoma*.mp,kw. | 24 | Advanced |  |  |  |
| 53 | DCIS.mp,kw. | 631 | Advanced |  |  |  |
| 54 | DSRCT.mp,kw. | 8 | Advanced |  |  |  |
| 55 | ependymom*.mp,kw. | 146 | Advanced |  |  |  |
| 56 | Ewing*.mp,kw. | 436 | Advanced |  |  |  |
| 57 | fibroadenom*.mp,kw. | 139 | Advanced |  |  |  |
| 58 | fibroepithelial*.mp,kw. | 4 | Advanced |  |  |  |
| 59 | fibroma*.mp,kw. | 223 | Advanced |  |  |  |
| 60 | fibrosarcom*.mp,kw. | 73 | Advanced |  |  |  |
| 61 | FAMMM.mp,kw. | 3 | Advanced |  |  |  |
| 62 | gangliogliom*.mp,kw. | 15 | Advanced |  |  |  |
| 63 | ganglioneurom*.mp,kw. | 17 | Advanced |  |  |  |
| 64 | gastrinoma*.mp,kw. | 29 | Advanced |  |  |  |
| 65 | germinoma*.mp,kw. | 119 | Advanced |  |  |  |
| 66 | glioblastom*.mp,kw. | 2554 | Advanced |  |  |  |
| 67 | glioma*.mp,kw. | 2146 | Advanced |  |  |  |
| 68 | gliosarcom*.mp,kw. | 82 | Advanced |  |  |  |
| 69 | glomus jugulare*.mp,kw. | 0 | Advanced |  |  |  |
| 70 | glomus tympanicum*.mp,kw. | 1 | Advanced |  |  |  |
| 71 | glucagonoma*.mp,kw. | 12 | Advanced |  |  |  |
| 72 | gonadoblastom*.mp,kw. | 1 | Advanced |  |  |  |
| 73 | GCTOB.mp,kw. | 0 | Advanced |  |  |  |
| 74 | GIST?.mp,kw. | 613 | Advanced |  |  |  |
| 75 | hemangioendotheliom*.mp,kw. | 28 | Advanced |  |  |  |
| 76 | hemangiom*.mp,kw. | 523 | Advanced |  |  |  |
| 77 | hemangiopericytom*.mp,kw. | 19 | Advanced |  |  |  |
| 78 | hemangiosarcom*.mp,kw. | 13 | Advanced |  |  |  |
| 79 | hamartoblastom*.mp,kw. | 0 | Advanced |  |  |  |
| 80 | hepatoblastom*.mp,kw. | 113 | Advanced |  |  |  |
| 81 | hepatoma*.mp,kw. | 157 | Advanced |  |  |  |
| 82 | histiocytom*.mp,kw. | 80 | Advanced |  |  |  |
| 83 | hodgkin*.mp,kw. | 6272 | Advanced |  |  |  |
| 84 | nonhodgkin*.mp,kw. | 1018 | Advanced |  |  |  |
| 85 | (hutchinson* adj2 freckle*).mp,kw. | 8 | Advanced |  |  |  |
| 86 | HNPCC.mp,kw. | 48 | Advanced |  |  |  |
| 87 | immunocytom*.mp,kw. | 24 | Advanced |  |  |  |
| 88 | incidentaloma?.mp,kw. | 43 | Advanced |  |  |  |
| 89 | insulinoma*.mp,kw. | 95 | Advanced |  |  |  |
| 90 | kasabach merrit*.mp,kw. | 10 | Advanced |  |  |  |
| 91 | leiomyoblastom*.mp,kw. | 1 | Advanced |  |  |  |
| 92 | leiomyom*.mp,kw. | 1153 | Advanced |  |  |  |
| 93 | leiomyosarcom*.mp,kw. | 332 | Advanced |  |  |  |
| 94 | leukem*.mp,kw. | 15827 | Advanced |  |  |  |
| 95 | preleukem*.mp,kw. | 365 | Advanced |  |  |  |
| 96 | leukoplak*.mp,kw. | 271 | Advanced |  |  |  |
| 97 | li-fraumeni*.mp,kw. | 14 | Advanced |  |  |  |
| 98 | lipoblastom*.mp,kw. | 0 | Advanced |  |  |  |
| 99 | lipoma*.mp,kw. | 88 | Advanced |  |  |  |
| 100 | liposarcom*.mp,kw. | 272 | Advanced |  |  |  |
| 101 | luteoma*.mp,kw. | 0 | Advanced |  |  |  |
| 102 | lymphangio*.mp,kw. | 252 | Advanced |  |  |  |
| 103 | lymphoblastom*.mp,kw. | 30 | Advanced |  |  |  |
| 104 | lymphocytom*.mp,kw. | 35 | Advanced |  |  |  |
| 105 | lymphoma*.mp,kw. | 13744 | Advanced |  |  |  |
| 106 | lymphosarcom*.mp,kw. | 27 | Advanced |  |  |  |
| 107 | lynch*.mp,kw. | 224 | Advanced |  |  |  |
| 108 | macroglobulinem*.mp,kw. | 215 | Advanced |  |  |  |
| 109 | m?croprolactinom*.mp,kw. | 37 | Advanced |  |  |  |
| 110 | mastocytom*.mp,kw. | 10 | Advanced |  |  |  |
| 111 | mastocytos?s*.mp,kw. | 116 | Advanced |  |  |  |
| 112 | medulloblastom*.mp,kw. | 326 | Advanced |  |  |  |
| 113 | meigs*.mp,kw. | 12 | Advanced |  |  |  |
| 114 | melanoameloblastom*.mp,kw. | 0 | Advanced |  |  |  |
| 115 | melanoblastom*.mp,kw. | 0 | Advanced |  |  |  |
| 116 | melanocarcin*.mp,kw. | 0 | Advanced |  |  |  |
| 117 | melanoma*.mp,kw. | 6596 | Advanced |  |  |  |
| 118 | melanosis.mp,kw. | 307 | Advanced |  |  |  |
| 119 | melanotic*.mp,kw. | 28 | Advanced |  |  |  |
| 120 | meningiom*.mp,kw. | 332 | Advanced |  |  |  |
| 121 | mesenchymom*.mp,kw. | 20 | Advanced |  |  |  |
| 122 | mesoblast*.mp,kw. | 18 | Advanced |  |  |  |
| 123 | mesonephrom*.mp,kw. | 4 | Advanced |  |  |  |
| 124 | mesotheliom*.mp,kw. | 916 | Advanced |  |  |  |
| 125 | metaplas*.mp,kw. | 911 | Advanced |  |  |  |
| 126 | micrometast*.mp,kw. | 451 | Advanced |  |  |  |
| 127 | muir-torre*.mp,kw. | 4 | Advanced |  |  |  |
| 128 | myelolipom*.mp,kw. | 8 | Advanced |  |  |  |
| 129 | myoepitheliom*.mp,kw. | 6 | Advanced |  |  |  |
| 130 | myofibrom*.mp,kw. | 185 | Advanced |  |  |  |
| 131 | myeloma*.mp,kw. | 6536 | Advanced |  |  |  |
| 132 | myoma*.mp,kw. | 1293 | Advanced |  |  |  |
| 133 | myosarcom*.mp,kw. | 2 | Advanced |  |  |  |
| 134 | myxofibrosarcom*.mp,kw. | 23 | Advanced |  |  |  |
| 135 | myxoma*.mp,kw. | 47 | Advanced |  |  |  |
| 136 | myxosarcom*.mp,kw. | 27 | Advanced |  |  |  |
| 137 | n?evocarcin*.mp,kw. | 1 | Advanced |  |  |  |
| 138 | neurilemmom*.mp,kw. | 21 | Advanced |  |  |  |
| 139 | neurocytom*.mp,kw. | 0 | Advanced |  |  |  |
| 140 | neuroectodermal*.mp,kw. | 117 | Advanced |  |  |  |
| 141 | neurofibroma*.mp,kw. | 217 | Advanced |  |  |  |
| 142 | neurofibrosarcom*.mp,kw. | 10 | Advanced |  |  |  |
| 143 | neurilemmom*.mp,kw. | 21 | Advanced |  |  |  |
| 144 | neuroblastom*.mp,kw. | 625 | Advanced |  |  |  |
| 145 | neuroma*.mp,kw. | 432 | Advanced |  |  |  |
| 146 | neurothekeom*.mp,kw. | 0 | Advanced |  |  |  |
| 147 | NSCLC.mp,kw. | 10825 | Advanced |  |  |  |
| 148 | odontoma*.mp,kw. | 1 | Advanced |  |  |  |
| 149 | oligo*.mp,kw. | 7526 | Advanced |  |  |  |
| 150 | osteoblastom*.mp,kw. | 2 | Advanced |  |  |  |
| 151 | osteochondrom*.mp,kw. | 10 | Advanced |  |  |  |
| 152 | osteoclastom*.mp,kw. | 12 | Advanced |  |  |  |
| 153 | osteoma*.mp,kw. | 250 | Advanced |  |  |  |
| 154 | osteosarcom*.mp,kw. | 659 | Advanced |  |  |  |
| 155 | papilloma*.mp,kw. | 3269 | Advanced |  |  |  |
| 156 | papillary*.mp,kw. | 1922 | Advanced |  |  |  |
| 157 | paragangliom*.mp,kw. | 65 | Advanced |  |  |  |
| 158 | pheochromocytom*.mp,kw. | 189 | Advanced |  |  |  |
| 159 | phyllo?des*.mp,kw. | 20 | Advanced |  |  |  |
| 160 | pinealocytoma*.mp,kw. | 0 | Advanced |  |  |  |
| 161 | pinealoma*.mp,kw. | 10 | Advanced |  |  |  |
| 162 | pineoblastoma*.mp,kw. | 21 | Advanced |  |  |  |
| 163 | pineocytoma*.mp,kw. | 1 | Advanced |  |  |  |
| 164 | plasmacytom*.mp,kw. | 333 | Advanced |  |  |  |
| 165 | (polycythem* adj2 vera?).mp,kw. | 454 | Advanced |  |  |  |
| 166 | prolactinom*.mp,kw. | 107 | Advanced |  |  |  |
| 167 | retinoblastom*.mp,kw. | 184 | Advanced |  |  |  |
| 168 | rhabdoid*.mp,kw. | 56 | Advanced |  |  |  |
| 169 | rhabdomyom*.mp,kw. | 8 | Advanced |  |  |  |
| 170 | rhabdomyosarcom*.mp,kw. | 331 | Advanced |  |  |  |
| 171 | sarcom*.mp,kw. | 3186 | Advanced |  |  |  |
| 172 | seminoma*.mp,kw. | 256 | Advanced |  |  |  |
| 173 | Sertoli- Leydig.mp,kw. | 10 | Advanced |  |  |  |
| 174 | somatostatinoma*.mp,kw. | 3 | Advanced |  |  |  |
| 175 | somatotrophinom*.mp,kw. | 0 | Advanced |  |  |  |
| 176 | struma ovarii*.mp,kw. | 3 | Advanced |  |  |  |
| 177 | thecoma*.mp,kw. | 2 | Advanced |  |  |  |
| 178 | teratocarcin*.mp,kw. | 6 | Advanced |  |  |  |
| 179 | teratoma*.mp,kw. | 154 | Advanced |  |  |  |
| 180 | thymom*.mp,kw. | 197 | Advanced |  |  |  |
| 181 | trophoblast*.mp,kw. | 298 | Advanced |  |  |  |
| 182 | vipoma*.mp,kw. | 6 | Advanced |  |  |  |
| 183 | wilms*.mp,kw. | 268 | Advanced |  |  |  |
| 184 | or/1-183 | 293285 | Advanced |  |  |  |
| 185 | Wearable Electronic Devices/ | 121 | Advanced |  |  |  |
| 186 | Accelerometry/ | 561 | Advanced |  |  |  |
| 187 | Actigraphy/ | 542 | Advanced |  |  |  |
| 188 | Fitness Trackers/ | 136 | Advanced |  |  |  |
| 189 | Remote Sensing Technology/ | 50 | Advanced |  |  |  |
| 190 | Smart Glasses/ | 7 | Advanced |  |  |  |
| 191 | wearable?.mp,kw. | 1667 | Advanced |  |  |  |
| 192 | wear-able?.mp,kw. | 0 | Advanced |  |  |  |
| 193 | acceleromet?r*.mp,kw. | 4840 | Advanced |  |  |  |
| 194 | actigraph*.mp,kw. | 3271 | Advanced |  |  |  |
| 195 | (activit* adj3 mobile?).mp,kw. | 183 | Advanced |  |  |  |
| 196 | (activit* adj3 track*).mp,kw. | 911 | Advanced |  |  |  |
| 197 | (activit* adj3 monitor*).mp,kw. | 3270 | Advanced |  |  |  |
| 198 | (fitness* adj3 mobile?).mp,kw. | 18 | Advanced |  |  |  |
| 199 | (fitness* adj3 band?).mp,kw. | 11 | Advanced |  |  |  |
| 200 | (fitness* adj3 device?).mp,kw. | 42 | Advanced |  |  |  |
| 201 | (fitness* adj3 track*).mp,kw. | 239 | Advanced |  |  |  |
| 202 | (fitness* adj3 monitor*).mp,kw. | 82 | Advanced |  |  |  |
| 203 | (move* adj3 mobile?).mp,kw. | 33 | Advanced |  |  |  |
| 204 | movemonitor*.mp,kw. | 11 | Advanced |  |  |  |
| 205 | move-monitor*.mp,kw. | 6 | Advanced |  |  |  |
| 206 | (physical* adj3 mobile?).mp,kw. | 186 | Advanced |  |  |  |
| 207 | (physical* adj3 track*).mp,kw. | 317 | Advanced |  |  |  |
| 208 | ((selfmonitor* or self-monitor*) adj3 mobile?).mp,kw. | 65 | Advanced |  |  |  |
| 209 | ((selfmonitor* or self-monitor*) adj3 device?).mp,kw. | 94 | Advanced |  |  |  |
| 210 | selftrack*.mp,kw. | 0 | Advanced |  |  |  |
| 211 | (self adj3 track*).mp,kw. | 170 | Advanced |  |  |  |
| 212 | ((wellness* or well-ness*) adj3 mobile?).mp,kw. | 10 | Advanced |  |  |  |
| 213 | ((wellness* or well-ness*) adj3 device?).mp,kw. | 12 | Advanced |  |  |  |
| 214 | ((wellness* or well-ness*) adj3 track*).mp,kw. | 11 | Advanced |  |  |  |
| 215 | mtrack*.mp,kw. | 0 | Advanced |  |  |  |
| 216 | m-track*.mp,kw. | 32 | Advanced |  |  |  |
| 217 | etrack*.mp,kw. | 14 | Advanced |  |  |  |
| 218 | e-track*.mp,kw. | 7 | Advanced |  |  |  |
| 219 | ((biometric? or bio-metric?) adj3 mobile?).mp,kw. | 7 | Advanced |  |  |  |
| 220 | ((biometric? or bio-metric?) adj3 monitor*).mp,kw. | 17 | Advanced |  |  |  |
| 221 | ((biometric? or bio-metric?) adj3 sensor?).mp,kw. | 10 | Advanced |  |  |  |
| 222 | (remote* adj3 sensing).mp,kw. | 406 | Advanced |  |  |  |
| 223 | (embedded adj3 sensor?).mp,kw. | 54 | Advanced |  |  |  |
| 224 | (movement? adj3 sensor?).mp,kw. | 261 | Advanced |  |  |  |
| 225 | (ehealth* adj3 mobile?).mp,kw. | 13 | Advanced |  |  |  |
| 226 | (phealth* adj3 mobile?).mp,kw. | 0 | Advanced |  |  |  |
| 227 | (uhealth* adj3 mobile?).mp,kw. | 0 | Advanced |  |  |  |
| 228 | (smart* adj3 device?).mp,kw. | 565 | Advanced |  |  |  |
| 229 | (smart* adj3 technolog*).mp,kw. | 450 | Advanced |  |  |  |
| 230 | (smart* adj3 wireless*).mp,kw. | 52 | Advanced |  |  |  |
| 231 | (smart* adj3 sensor?).mp,kw. | 138 | Advanced |  |  |  |
| 232 | (smart* adj3 biosensor?).mp,kw. | 3 | Advanced |  |  |  |
| 233 | (smart* adj3 monitor*).mp,kw. | 439 | Advanced |  |  |  |
| 234 | (smart* adj3 track*).mp,kw. | 139 | Advanced |  |  |  |
| 235 | (smart* adj3 health*).mp,kw. | 557 | Advanced |  |  |  |
| 236 | (smart* adj3 ehealth*).mp,kw. | 12 | Advanced |  |  |  |
| 237 | (smart* adj3 mhealth*).mp,kw. | 61 | Advanced |  |  |  |
| 238 | (smart* adj3 phealth*).mp,kw. | 0 | Advanced |  |  |  |
| 239 | (smart* adj3 uhealth*).mp,kw. | 0 | Advanced |  |  |  |
| 240 | (smart* adj3 (count or counts or counter?)).mp,kw. | 42 | Advanced |  |  |  |
| 241 | (smart* adj3 logger?).mp,kw. | 0 | Advanced |  |  |  |
| 242 | (smart* adj3 wear*).mp,kw. | 146 | Advanced |  |  |  |
| 243 | (smart* adj3 worn*).mp,kw. | 16 | Advanced |  |  |  |
| 244 | smartband?.mp,kw. | 12 | Advanced |  |  |  |
| 245 | (smart* adj3 band?).mp,kw. | 18 | Advanced |  |  |  |
| 246 | (smart* adj3 armband?).mp,kw. | 2 | Advanced |  |  |  |
| 247 | smartglass*.mp,kw. | 3 | Advanced |  |  |  |
| 248 | (smart* adj3 glass*).mp,kw. | 30 | Advanced |  |  |  |
| 249 | (smart* adj3 (eyewear? or eye-wear?)).mp,kw. | 0 | Advanced |  |  |  |
| 250 | smartring?.mp,kw. | 0 | Advanced |  |  |  |
| 251 | (smart* adj3 ring?).mp,kw. | 3 | Advanced |  |  |  |
| 252 | smartwatch*.mp,kw. | 100 | Advanced |  |  |  |
| 253 | (smart* adj3 watch*).mp,kw. | 75 | Advanced |  |  |  |
| 254 | (smart* adj3 cloth*).mp,kw. | 2 | Advanced |  |  |  |
| 255 | (smart* adj3 fabric?).mp,kw. | 1 | Advanced |  |  |  |
| 256 | (smart* adj3 apparel?).mp,kw. | 2 | Advanced |  |  |  |
| 257 | (smart* adj3 garment?).mp,kw. | 4 | Advanced |  |  |  |
| 258 | (electronic* adj3 textile?).mp,kw. | 0 | Advanced |  |  |  |
| 259 | (e-textile? or etextile?).mp,kw. | 1 | Advanced |  |  |  |
| 260 | (smart* adj3 textile?).mp,kw. | 2 | Advanced |  |  |  |
| 261 | (smart* adj3 cap?).mp,kw. | 13 | Advanced |  |  |  |
| 262 | (smart* adj3 dress*).mp,kw. | 3 | Advanced |  |  |  |
| 263 | (smart* adj3 glove?).mp,kw. | 23 | Advanced |  |  |  |
| 264 | (smart* adj3 hat?).mp,kw. | 0 | Advanced |  |  |  |
| 265 | (smart* adj3 helmet?).mp,kw. | 0 | Advanced |  |  |  |
| 266 | (smart* adj3 jacket?).mp,kw. | 0 | Advanced |  |  |  |
| 267 | (smart* adj3 insoles*).mp,kw. | 7 | Advanced |  |  |  |
| 268 | (smart* adj3 pant?).mp,kw. | 0 | Advanced |  |  |  |
| 269 | (smart* adj3 scarf?).mp,kw. | 0 | Advanced |  |  |  |
| 270 | (smart* adj3 shirt?).mp,kw. | 6 | Advanced |  |  |  |
| 271 | (smart* adj3 footwear?).mp,kw. | 1 | Advanced |  |  |  |
| 272 | (smart* adj3 shoe?).mp,kw. | 9 | Advanced |  |  |  |
| 273 | (smart* adj3 sock?).mp,kw. | 3 | Advanced |  |  |  |
| 274 | (smart* adj3 strap?).mp,kw. | 1 | Advanced |  |  |  |
| 275 | (smart* adj3 suit?).mp,kw. | 10 | Advanced |  |  |  |
| 276 | (smart* adj3 vest?).mp,kw. | 2 | Advanced |  |  |  |
| 277 | (smart* adj3 societ*).mp,kw. | 13 | Advanced |  |  |  |
| 278 | (intelligent adj3 sensor?).mp,kw. | 13 | Advanced |  |  |  |
| 279 | (intelligen* adj3 biosensor?).mp,kw. | 0 | Advanced |  |  |  |
| 280 | (intelligen* adj3 wear*).mp,kw. | 7 | Advanced |  |  |  |
| 281 | (intelligen* adj3 worn*).mp,kw. | 0 | Advanced |  |  |  |
| 282 | (intelligen* adj3 band?).mp,kw. | 3 | Advanced |  |  |  |
| 283 | (intelligen* adj3 armband?).mp,kw. | 1 | Advanced |  |  |  |
| 284 | (intelligen* adj3 glass*).mp,kw. | 1 | Advanced |  |  |  |
| 285 | (intelligen* adj3 (eyewear? or eye-wear?)).mp,kw. | 0 | Advanced |  |  |  |
| 286 | (intelligen* adj3 ring?).mp,kw. | 1 | Advanced |  |  |  |
| 287 | (intelligen* adj3 watch*).mp,kw. | 1 | Advanced |  |  |  |
| 288 | (intelligen* adj3 cloth*).mp,kw. | 3 | Advanced |  |  |  |
| 289 | (intelligen* adj3 fabric?).mp,kw. | 0 | Advanced |  |  |  |
| 290 | (intelligen* adj3 apparel?).mp,kw. | 0 | Advanced |  |  |  |
| 291 | (intelligen* adj3 garment?).mp,kw. | 0 | Advanced |  |  |  |
| 292 | (intelligen* adj3 textile?).mp,kw. | 0 | Advanced |  |  |  |
| 293 | (intelligen* adj3 belt?).mp,kw. | 0 | Advanced |  |  |  |
| 294 | (intelligen* adj3 cap?).mp,kw. | 1 | Advanced |  |  |  |
| 295 | (intelligen* adj3 dress*).mp,kw. | 0 | Advanced |  |  |  |
| 296 | (intelligen* adj3 glove?).mp,kw. | 0 | Advanced |  |  |  |
| 297 | (intelligen* adj3 hat?).mp,kw. | 1 | Advanced |  |  |  |
| 298 | (intelligen* adj3 helmet?).mp,kw. | 0 | Advanced |  |  |  |
| 299 | (intelligen* adj3 jacket?).mp,kw. | 0 | Advanced |  |  |  |
| 300 | (intelligen* adj3 insoles*).mp,kw. | 2 | Advanced |  |  |  |
| 301 | (intelligen* adj3 pant?).mp,kw. | 0 | Advanced |  |  |  |
| 302 | (intelligen* adj3 scarf?).mp,kw. | 0 | Advanced |  |  |  |
| 303 | (intelligen* adj3 shirt?).mp,kw. | 0 | Advanced |  |  |  |
| 304 | (intelligen* adj3 footwear?).mp,kw. | 0 | Advanced |  |  |  |
| 305 | (intelligen* adj3 shoe?).mp,kw. | 1 | Advanced |  |  |  |
| 306 | (intelligen* adj3 sock?).mp,kw. | 0 | Advanced |  |  |  |
| 307 | (intelligen* adj3 strap?).mp,kw. | 0 | Advanced |  |  |  |
| 308 | (intelligen* adj3 suit?).mp,kw. | 1 | Advanced |  |  |  |
| 309 | (intelligen* adj3 vest?).mp,kw. | 0 | Advanced |  |  |  |
| 310 | I-wear*.mp,kw. | 8 | Advanced |  |  |  |
| 311 | (ambient* adj3 intelligen*).mp,kw. | 0 | Advanced |  |  |  |
| 312 | (ambient* adj3 wear*).mp,kw. | 4 | Advanced |  |  |  |
| 313 | (ambient* adj3 worn*).mp,kw. | 1 | Advanced |  |  |  |
| 314 | sensor?-wear*.mp,kw. | 34 | Advanced |  |  |  |
| 315 | sens*wear*.mp,kw. | 186 | Advanced |  |  |  |
| 316 | (wireless* adj3 wear*).mp,kw. | 49 | Advanced |  |  |  |
| 317 | (wireless* adj3 worn*).mp,kw. | 13 | Advanced |  |  |  |
| 318 | (wireless* adj3 biosensor*).mp,kw. | 2 | Advanced |  |  |  |
| 319 | (wireless* adj3 textile?).mp,kw. | 0 | Advanced |  |  |  |
| 320 | (wireless body adj3 area network?).mp,kw. | 0 | Advanced |  |  |  |
| 321 | (wireless sensor? adj3 network-based).mp,kw. | 0 | Advanced |  |  |  |
| 322 | WBAN?.mp,kw. | 0 | Advanced |  |  |  |
| 323 | ankle?-worn*.mp,kw. | 7 | Advanced |  |  |  |
| 324 | ankle?-mount*.mp,kw. | 4 | Advanced |  |  |  |
| 325 | finger?-worn*.mp,kw. | 6 | Advanced |  |  |  |
| 326 | waist?-worn*.mp,kw. | 43 | Advanced |  |  |  |
| 327 | waist?-mount*.mp,kw. | 12 | Advanced |  |  |  |
| 328 | wrist?-worn*.mp,kw. | 327 | Advanced |  |  |  |
| 329 | wrist?-mount*.mp,kw. | 16 | Advanced |  |  |  |
| 330 | pedometer?.mp,kw. | 1936 | Advanced |  |  |  |
| 331 | step?count*.mp,kw. | 7 | Advanced |  |  |  |
| 332 | (step? adj3 count*).mp,kw. | 1309 | Advanced |  |  |  |
| 333 | (adidas adj3 watch*).mp,kw. | 0 | Advanced |  |  |  |
| 334 | (apple adj3 watch*).mp,kw. | 51 | Advanced |  |  |  |
| 335 | (boat adj3 watch*).mp,kw. | 0 | Advanced |  |  |  |
| 336 | (casio adj3 watch*).mp,kw. | 1 | Advanced |  |  |  |
| 337 | amazfit?.mp,kw. | 2 | Advanced |  |  |  |
| 338 | fitbit?.mp,kw. | 645 | Advanced |  |  |  |
| 339 | (fossil adj3 watch*).mp,kw. | 0 | Advanced |  |  |  |
| 340 | garmin?.mp,kw. | 70 | Advanced |  |  |  |
| 341 | (hexoskin adj3 watch*).mp,kw. | 0 | Advanced |  |  |  |
| 342 | (huawei adj3 watch*).mp,kw. | 0 | Advanced |  |  |  |
| 343 | (jawbone adj3 watch*).mp,kw. | 0 | Advanced |  |  |  |
| 344 | (lg adj3 watch*).mp,kw. | 0 | Advanced |  |  |  |
| 345 | misfit?.mp,kw. | 50 | Advanced |  |  |  |
| 346 | (mi adj3 watch*).mp,kw. | 7 | Advanced |  |  |  |
| 347 | (nike adj3 watch*).mp,kw. | 0 | Advanced |  |  |  |
| 348 | (puma adj3 watch*).mp,kw. | 0 | Advanced |  |  |  |
| 349 | (samsung adj3 watch*).mp,kw. | 5 | Advanced |  |  |  |
| 350 | (xiaomi adj3 watch*).mp,kw. | 1 | Advanced |  |  |  |
| 351 | whoop.mp,kw. | 22 | Advanced |  |  |  |
| 352 | (consumer? adj5 (base? or level? or grade?) adj5 device?).mp,kw. | 18 | Advanced |  |  |  |
| 353 | (consumer? adj5 (base? or level? or grade?) adj5 measur*).mp,kw. | 12 | Advanced |  |  |  |
| 354 | (consumer? adj5 (base? or level? or grade?) adj5 monitor*).mp,kw. | 10 | Advanced |  |  |  |
| 355 | (consumer? adj5 (base? or level? or grade?) adj5 tracker?).mp,kw. | 7 | Advanced |  |  |  |
| 356 | (consumer? adj5 activit* adj5 device?).mp,kw. | 2 | Advanced |  |  |  |
| 357 | (consumer? adj5 activit* adj5 measur*).mp,kw. | 7 | Advanced |  |  |  |
| 358 | (consumer? adj5 activit* adj5 monitor*).mp,kw. | 9 | Advanced |  |  |  |
| 359 | (consumer? adj5 activit* adj5 tracker?).mp,kw. | 10 | Advanced |  |  |  |
| 360 | (consumer? adj5 PAM?).mp,kw. | 2 | Advanced |  |  |  |
| 361 | (consumer? adj5 IMU?).mp,kw. | 0 | Advanced |  |  |  |
| 362 | or/185-361 | 17325 | Advanced |  |  |  |
| 363 | 184 and 362 | 1473 | Advanced |  |  |  |
| 364 | (abstract or book or book article or book or book note or "book review" or book series article or book series article in press or book series chapter or book series conference paper or book series letter or "book series review" or book series short survey or chapter or conference abstract or conference proceeding or "conference review" or journal conference abstract or "journal conference review").pt. | 206780 | Advanced |  |  |  |
| 365 | conferenc*.so. | 41159 | Advanced |  |  |  |
| 366 | "http?://clinicaltrial*".so. | 216582 | Advanced |  |  |  |
| 367 | "http?://www.who.int/*".so. | 1 | Advanced |  |  |  |
| 368 | "http?://trialsearch.who.int/*".so. | 166490 | Advanced |  |  |  |
| 369 | or/364-368 | 591766 | Advanced |  |  |  |
| 370 | 363 not 369 | 577 | Advanced |  |  |  |

**Ovid Embase Classic +Embase 1947 to 2022 February 17**

| **#** | **Searches** | **Results** | **Type** |  |  |  |
| --- | --- | --- | --- | --- | --- | --- |
|  | | | | | | |
| 1 | exp neoplasm/ | 5378271 | Advanced |  |  |  |
| 2 | exp cancer patient/ | 574369 | Advanced |  |  |  |
| 3 | survivor/ | 28828 | Advanced |  |  |  |
| 4 | neoplas*.mp,kw. | 1161973 | Advanced |  |  |  |
| 5 | paraneoplas*.mp,kw. | 23973 | Advanced |  |  |  |
| 6 | *.mp,kw. | 4196811 | Advanced |  |  |  |
| 7 | tumo?r*.mp,kw. | 3742729 | Advanced |  |  |  |
| 8 | onco*.mp,kw. | 836649 | Advanced |  |  |  |
| 9 | metast*.mp,kw. | 1043787 | Advanced |  |  |  |
| 10 | malignan*.mp,kw. | 1076617 | Advanced |  |  |  |
| 11 | aberrant crypt foci.mp,kw. | 1926 | Advanced |  |  |  |
| 12 | acanthoma*.mp,kw. | 1367 | Advanced |  |  |  |
| 13 | acrospiroma*.mp,kw. | 115 | Advanced |  |  |  |
| 14 | adamantinom*.mp,kw. | 1591 | Advanced |  |  |  |
| 15 | adenocarc*.mp,kw. | 330031 | Advanced |  |  |  |
| 16 | adenofibrom*.mp,kw. | 729 | Advanced |  |  |  |
| 17 | adenolymphom*.mp,kw. | 579 | Advanced |  |  |  |
| 18 | adenomat*.mp,kw. | 35908 | Advanced |  |  |  |
| 19 | adenomyo*.mp,kw. | 9108 | Advanced |  |  |  |
| 20 | adenosarcom*.mp,kw. | 1129 | Advanced |  |  |  |
| 21 | adenosquam*.mp,kw. | 9878 | Advanced |  |  |  |
| 22 | ameloblastom*.mp,kw. | 6804 | Advanced |  |  |  |
| 23 | androblastom*.mp,kw. | 1570 | Advanced |  |  |  |
| 24 | angiofibrom*.mp,kw. | 3267 | Advanced |  |  |  |
| 25 | angiokeratom*.mp,kw. | 1549 | Advanced |  |  |  |
| 26 | angiolipom*.mp,kw. | 1269 | Advanced |  |  |  |
| 27 | angioma*.mp,kw. | 18268 | Advanced |  |  |  |
| 28 | angiomyolipom*.mp,kw. | 7581 | Advanced |  |  |  |
| 29 | angiomyom*.mp,kw. | 250 | Advanced |  |  |  |
| 30 | angiosarcom*.mp,kw. | 14214 | Advanced |  |  |  |
| 31 | apudoma*.mp,kw. | 679 | Advanced |  |  |  |
| 32 | arrhenoblastom*.mp,kw. | 472 | Advanced |  |  |  |
| 33 | astrocytom*.mp,kw. | 36646 | Advanced |  |  |  |
| 34 | blastom*.mp,kw. | 20086 | Advanced |  |  |  |
| 35 | Bowen*.mp,kw. | 6575 | Advanced |  |  |  |
| 36 | Brenner*.mp,kw. | 2529 | Advanced |  |  |  |
| 37 | Buschke-Lowenstein*.mp,kw. | 381 | Advanced |  |  |  |
| 38 | carcin*.mp,kw. | 1776230 | Advanced |  |  |  |
| 39 | cementoma*.mp,kw. | 287 | Advanced |  |  |  |
| 40 | chemodectomas*.mp,kw. | 471 | Advanced |  |  |  |
| 41 | cholangiocarcin*.mp,kw. | 25397 | Advanced |  |  |  |
| 42 | chondroblastom*.mp,kw. | 2040 | Advanced |  |  |  |
| 43 | chondroma*.mp,kw. | 8951 | Advanced |  |  |  |
| 44 | chordoma*.mp,kw. | 7965 | Advanced |  |  |  |
| 45 | chondrosarcom*.mp,kw. | 15871 | Advanced |  |  |  |
| 46 | choriocarcin*.mp,kw. | 14402 | Advanced |  |  |  |
| 47 | craniopharyngioma*.mp,kw. | 9811 | Advanced |  |  |  |
| 48 | cystadenofibrom*.mp,kw. | 421 | Advanced |  |  |  |
| 49 | cystosarcom*.mp,kw. | 2604 | Advanced |  |  |  |
| 50 | cytoma*.mp,kw. | 641 | Advanced |  |  |  |
| 51 | dermatofibrosarcom*.mp,kw. | 3948 | Advanced |  |  |  |
| 52 | desmoplas*.mp,kw. | 9052 | Advanced |  |  |  |
| 53 | dysgerminoma*.mp,kw. | 4778 | Advanced |  |  |  |
| 54 | DCIS.mp,kw. | 10450 | Advanced |  |  |  |
| 55 | DSRCT.mp,kw. | 542 | Advanced |  |  |  |
| 56 | ependymom*.mp,kw. | 13126 | Advanced |  |  |  |
| 57 | Ewing*.mp,kw. | 21217 | Advanced |  |  |  |
| 58 | fibroadenom*.mp,kw. | 8492 | Advanced |  |  |  |
| 59 | fibroepithelial*.mp,kw. | 1700 | Advanced |  |  |  |
| 60 | fibroma*.mp,kw. | 28832 | Advanced |  |  |  |
| 61 | fibrosarcom*.mp,kw. | 22877 | Advanced |  |  |  |
| 62 | FAMMM.mp,kw. | 133 | Advanced |  |  |  |
| 63 | gangliogliom*.mp,kw. | 2464 | Advanced |  |  |  |
| 64 | ganglioneurom*.mp,kw. | 5286 | Advanced |  |  |  |
| 65 | gastrinoma*.mp,kw. | 3417 | Advanced |  |  |  |
| 66 | germinoma*.mp,kw. | 5372 | Advanced |  |  |  |
| 67 | glioblastom*.mp,kw. | 91896 | Advanced |  |  |  |
| 68 | glioma*.mp,kw. | 112762 | Advanced |  |  |  |
| 69 | gliosarcom*.mp,kw. | 2150 | Advanced |  |  |  |
| 70 | glomus jugulare*.mp,kw. | 1384 | Advanced |  |  |  |
| 71 | glomus tympanicum*.mp,kw. | 364 | Advanced |  |  |  |
| 72 | glucagonoma*.mp,kw. | 2007 | Advanced |  |  |  |
| 73 | gonadoblastom*.mp,kw. | 1549 | Advanced |  |  |  |
| 74 | GCTOB.mp,kw. | 11 | Advanced |  |  |  |
| 75 | GIST?.mp,kw. | 15229 | Advanced |  |  |  |
| 76 | hemangioendotheliom*.mp,kw. | 6450 | Advanced |  |  |  |
| 77 | hemangiom*.mp,kw. | 56170 | Advanced |  |  |  |
| 78 | hemangiopericytom*.mp,kw. | 5976 | Advanced |  |  |  |
| 79 | hemangiosarcom*.mp,kw. | 1766 | Advanced |  |  |  |
| 80 | hamartoblastom*.mp,kw. | 84 | Advanced |  |  |  |
| 81 | hepatoblastom*.mp,kw. | 7081 | Advanced |  |  |  |
| 82 | hepatoma*.mp,kw. | 40600 | Advanced |  |  |  |
| 83 | histiocytom*.mp,kw. | 10110 | Advanced |  |  |  |
| 84 | hodgkin*.mp,kw. | 130933 | Advanced |  |  |  |
| 85 | nonhodgkin*.mp,kw. | 67236 | Advanced |  |  |  |
| 86 | (hutchinson* adj2 freckle*).mp,kw. | 114 | Advanced |  |  |  |
| 87 | HNPCC.mp,kw. | 3303 | Advanced |  |  |  |
| 88 | immunocytom*.mp,kw. | 854 | Advanced |  |  |  |
| 89 | incidentaloma?.mp,kw. | 4408 | Advanced |  |  |  |
| 90 | insulinoma*.mp,kw. | 12496 | Advanced |  |  |  |
| 91 | kasabach merrit*.mp,kw. | 1344 | Advanced |  |  |  |
| 92 | leiomyoblastom*.mp,kw. | 730 | Advanced |  |  |  |
| 93 | leiomyom*.mp,kw. | 31793 | Advanced |  |  |  |
| 94 | leiomyosarcom*.mp,kw. | 20662 | Advanced |  |  |  |
| 95 | leukem*.mp,kw. | 542820 | Advanced |  |  |  |
| 96 | preleukem*.mp,kw. | 3009 | Advanced |  |  |  |
| 97 | leukoplak*.mp,kw. | 10341 | Advanced |  |  |  |
| 98 | li-fraumeni*.mp,kw. | 2292 | Advanced |  |  |  |
| 99 | lipoblastom*.mp,kw. | 880 | Advanced |  |  |  |
| 100 | lipoma*.mp,kw. | 27780 | Advanced |  |  |  |
| 101 | liposarcom*.mp,kw. | 13345 | Advanced |  |  |  |
| 102 | luteoma*.mp,kw. | 463 | Advanced |  |  |  |
| 103 | lymphangio*.mp,kw. | 27529 | Advanced |  |  |  |
| 104 | lymphoblastom*.mp,kw. | 4029 | Advanced |  |  |  |
| 105 | lymphocytom*.mp,kw. | 3497 | Advanced |  |  |  |
| 106 | lymphoma*.mp,kw. | 381824 | Advanced |  |  |  |
| 107 | lymphosarcom*.mp,kw. | 9890 | Advanced |  |  |  |
| 108 | lynch*.mp,kw. | 8792 | Advanced |  |  |  |
| 109 | macroglobulinem*.mp,kw. | 11321 | Advanced |  |  |  |
| 110 | m?croprolactinom*.mp,kw. | 1227 | Advanced |  |  |  |
| 111 | mastocytom*.mp,kw. | 3912 | Advanced |  |  |  |
| 112 | mastocytos?s*.mp,kw. | 8056 | Advanced |  |  |  |
| 113 | medulloblastom*.mp,kw. | 20252 | Advanced |  |  |  |
| 114 | meigs*.mp,kw. | 1245 | Advanced |  |  |  |
| 115 | melanoameloblastom*.mp,kw. | 152 | Advanced |  |  |  |
| 116 | melanoblastom*.mp,kw. | 838 | Advanced |  |  |  |
| 117 | melanocarcin*.mp,kw. | 162 | Advanced |  |  |  |
| 118 | melanoma*.mp,kw. | 235939 | Advanced |  |  |  |
| 119 | melanosis.mp,kw. | 5739 | Advanced |  |  |  |
| 120 | melanotic*.mp,kw. | 3692 | Advanced |  |  |  |
| 121 | meningiom*.mp,kw. | 42607 | Advanced |  |  |  |
| 122 | mesenchymom*.mp,kw. | 4283 | Advanced |  |  |  |
| 123 | mesoblast*.mp,kw. | 1538 | Advanced |  |  |  |
| 124 | mesonephrom*.mp,kw. | 875 | Advanced |  |  |  |
| 125 | mesotheliom*.mp,kw. | 32759 | Advanced |  |  |  |
| 126 | metaplas*.mp,kw. | 49109 | Advanced |  |  |  |
| 127 | micrometast*.mp,kw. | 12652 | Advanced |  |  |  |
| 128 | muir-torre*.mp,kw. | 843 | Advanced |  |  |  |
| 129 | myelolipom*.mp,kw. | 1872 | Advanced |  |  |  |
| 130 | myoepitheliom*.mp,kw. | 1876 | Advanced |  |  |  |
| 131 | myofibrom*.mp,kw. | 994 | Advanced |  |  |  |
| 132 | myeloma*.mp,kw. | 125672 | Advanced |  |  |  |
| 133 | myoma*.mp,kw. | 26159 | Advanced |  |  |  |
| 134 | myosarcom*.mp,kw. | 730 | Advanced |  |  |  |
| 135 | myxofibrosarcom*.mp,kw. | 1100 | Advanced |  |  |  |
| 136 | myxoma*.mp,kw. | 15935 | Advanced |  |  |  |
| 137 | myxosarcom*.mp,kw. | 2832 | Advanced |  |  |  |
| 138 | n?evocarcin*.mp,kw. | 119 | Advanced |  |  |  |
| 139 | neurilemmom*.mp,kw. | 2667 | Advanced |  |  |  |
| 140 | neurocytom*.mp,kw. | 1134 | Advanced |  |  |  |
| 141 | neuroectodermal*.mp,kw. | 9319 | Advanced |  |  |  |
| 142 | neurofibroma*.mp,kw. | 34291 | Advanced |  |  |  |
| 143 | neurofibrosarcom*.mp,kw. | 1031 | Advanced |  |  |  |
| 144 | neurilemmom*.mp,kw. | 2667 | Advanced |  |  |  |
| 145 | neuroblastom*.mp,kw. | 64443 | Advanced |  |  |  |
| 146 | neuroma*.mp,kw. | 19438 | Advanced |  |  |  |
| 147 | neurothekeom*.mp,kw. | 389 | Advanced |  |  |  |
| 148 | NSCLC.mp,kw. | 97363 | Advanced |  |  |  |
| 149 | odontoma*.mp,kw. | 1624 | Advanced |  |  |  |
| 150 | oligo*.mp,kw. | 431574 | Advanced |  |  |  |
| 151 | osteoblastom*.mp,kw. | 2218 | Advanced |  |  |  |
| 152 | osteochondrom*.mp,kw. | 5680 | Advanced |  |  |  |
| 153 | osteoclastom*.mp,kw. | 2797 | Advanced |  |  |  |
| 154 | osteoma*.mp,kw. | 21801 | Advanced |  |  |  |
| 155 | osteosarcom*.mp,kw. | 52192 | Advanced |  |  |  |
| 156 | papilloma*.mp,kw. | 102823 | Advanced |  |  |  |
| 157 | papillary*.mp,kw. | 105955 | Advanced |  |  |  |
| 158 | paragangliom*.mp,kw. | 12984 | Advanced |  |  |  |
| 159 | pheochromocytom*.mp,kw. | 35812 | Advanced |  |  |  |
| 160 | phyllo?des*.mp,kw. | 4174 | Advanced |  |  |  |
| 161 | pinealocytoma*.mp,kw. | 27 | Advanced |  |  |  |
| 162 | pinealoma*.mp,kw. | 624 | Advanced |  |  |  |
| 163 | pineoblastoma*.mp,kw. | 871 | Advanced |  |  |  |
| 164 | pineocytoma*.mp,kw. | 373 | Advanced |  |  |  |
| 165 | plasmacytom*.mp,kw. | 18315 | Advanced |  |  |  |
| 166 | (polycythem* adj2 vera?).mp,kw. | 14739 | Advanced |  |  |  |
| 167 | prolactinom*.mp,kw. | 7765 | Advanced |  |  |  |
| 168 | retinoblastom*.mp,kw. | 34143 | Advanced |  |  |  |
| 169 | rhabdoid*.mp,kw. | 5839 | Advanced |  |  |  |
| 170 | rhabdomyom*.mp,kw. | 2898 | Advanced |  |  |  |
| 171 | rhabdomyosarcom*.mp,kw. | 25340 | Advanced |  |  |  |
| 172 | sarcom*.mp,kw. | 209679 | Advanced |  |  |  |
| 173 | seminoma*.mp,kw. | 14513 | Advanced |  |  |  |
| 174 | Sertoli- Leydig.mp,kw. | 979 | Advanced |  |  |  |
| 175 | somatostatinoma*.mp,kw. | 863 | Advanced |  |  |  |
| 176 | somatotrophinom*.mp,kw. | 109 | Advanced |  |  |  |
| 177 | struma ovarii*.mp,kw. | 906 | Advanced |  |  |  |
| 178 | thecoma*.mp,kw. | 1478 | Advanced |  |  |  |
| 179 | teratocarcin*.mp,kw. | 3647 | Advanced |  |  |  |
| 180 | teratoma*.mp,kw. | 41340 | Advanced |  |  |  |
| 181 | thymom*.mp,kw. | 19309 | Advanced |  |  |  |
| 182 | trophoblast*.mp,kw. | 39329 | Advanced |  |  |  |
| 183 | vipoma*.mp,kw. | 1010 | Advanced |  |  |  |
| 184 | wilms*.mp,kw. | 14807 | Advanced |  |  |  |
| 185 | or/1-184 | 7849470 | Advanced |  |  |  |
| 186 | exp wearable computer/ | 6018 | Advanced |  |  |  |
| 187 | exp head-mounted display/ | 812 | Advanced |  |  |  |
| 188 | smart glasses/ | 180 | Advanced |  |  |  |
| 189 | exp smart watch/ | 4435 | Advanced |  |  |  |
| 190 | exp activity tracker/ | 4110 | Advanced |  |  |  |
| 191 | pedometer/ | 2595 | Advanced |  |  |  |
| 192 | accelerometer/ | 14869 | Advanced |  |  |  |
| 193 | accelerometry/ | 8781 | Advanced |  |  |  |
| 194 | actimetry/ | 10671 | Advanced |  |  |  |
| 195 | actigraph/ | 1875 | Advanced |  |  |  |
| 196 | remote sensing/ | 11781 | Advanced |  |  |  |
| 197 | smart material/ | 257 | Advanced |  |  |  |
| 198 | body area network/ | 162 | Advanced |  |  |  |
| 199 | wearable?.mp,kw. | 21962 | Advanced |  |  |  |
| 200 | wear-able?.mp,kw. | 9 | Advanced |  |  |  |
| 201 | acceleromet?r*.mp,kw. | 29087 | Advanced |  |  |  |
| 202 | actigraph*.mp,kw. | 14457 | Advanced |  |  |  |
| 203 | (activit* adj3 mobile?).mp,kw. | 503 | Advanced |  |  |  |
| 204 | (activit* adj3 track*).mp,kw. | 4188 | Advanced |  |  |  |
| 205 | (activit* adj3 monitor*).mp,kw. | 26799 | Advanced |  |  |  |
| 206 | (fitness* adj3 mobile?).mp,kw. | 32 | Advanced |  |  |  |
| 207 | (fitness* adj3 band?).mp,kw. | 29 | Advanced |  |  |  |
| 208 | (fitness* adj3 device?).mp,kw. | 153 | Advanced |  |  |  |
| 209 | (fitness* adj3 track*).mp,kw. | 604 | Advanced |  |  |  |
| 210 | (fitness* adj3 monitor*).mp,kw. | 373 | Advanced |  |  |  |
| 211 | (move* adj3 mobile?).mp,kw. | 459 | Advanced |  |  |  |
| 212 | movemonitor*.mp,kw. | 53 | Advanced |  |  |  |
| 213 | move-monitor*.mp,kw. | 6 | Advanced |  |  |  |
| 214 | (physical* adj3 mobile?).mp,kw. | 225 | Advanced |  |  |  |
| 215 | (physical* adj3 track*).mp,kw. | 787 | Advanced |  |  |  |
| 216 | ((selfmonitor* or self-monitor*) adj3 mobile?).mp,kw. | 84 | Advanced |  |  |  |
| 217 | ((selfmonitor* or self-monitor*) adj3 device?).mp,kw. | 255 | Advanced |  |  |  |
| 218 | selftrack*.mp,kw. | 5 | Advanced |  |  |  |
| 219 | (self adj3 track*).mp,kw. | 740 | Advanced |  |  |  |
| 220 | ((wellness* or well-ness*) adj3 mobile?).mp,kw. | 43 | Advanced |  |  |  |
| 221 | ((wellness* or well-ness*) adj3 device?).mp,kw. | 32 | Advanced |  |  |  |
| 222 | ((wellness* or well-ness*) adj3 track*).mp,kw. | 41 | Advanced |  |  |  |
| 223 | mtrack*.mp,kw. | 17 | Advanced |  |  |  |
| 224 | m-track*.mp,kw. | 172 | Advanced |  |  |  |
| 225 | etrack*.mp,kw. | 50 | Advanced |  |  |  |
| 226 | e-track*.mp,kw. | 107 | Advanced |  |  |  |
| 227 | ((biometric? or bio-metric?) adj3 mobile?).mp,kw. | 26 | Advanced |  |  |  |
| 228 | ((biometric? or bio-metric?) adj3 monitor*).mp,kw. | 105 | Advanced |  |  |  |
| 229 | ((biometric? or bio-metric?) adj3 sensor?).mp,kw. | 95 | Advanced |  |  |  |
| 230 | (remote* adj3 sensing).mp,kw. | 16242 | Advanced |  |  |  |
| 231 | (embedded adj3 sensor?).mp,kw. | 1254 | Advanced |  |  |  |
| 232 | (movement? adj3 sensor?).mp,kw. | 1925 | Advanced |  |  |  |
| 233 | (ehealth* adj3 mobile?).mp,kw. | 102 | Advanced |  |  |  |
| 234 | (phealth* adj3 mobile?).mp,kw. | 0 | Advanced |  |  |  |
| 235 | (uhealth* adj3 mobile?).mp,kw. | 2 | Advanced |  |  |  |
| 236 | (smart* adj3 device?).mp,kw. | 3778 | Advanced |  |  |  |
| 237 | (smart* adj3 technolog*).mp,kw. | 3344 | Advanced |  |  |  |
| 238 | (smart* adj3 wireless*).mp,kw. | 511 | Advanced |  |  |  |
| 239 | (smart* adj3 sensor?).mp,kw. | 1939 | Advanced |  |  |  |
| 240 | (smart* adj3 biosensor?).mp,kw. | 229 | Advanced |  |  |  |
| 241 | (smart* adj3 monitor*).mp,kw. | 1426 | Advanced |  |  |  |
| 242 | (smart* adj3 track*).mp,kw. | 427 | Advanced |  |  |  |
| 243 | (smart* adj3 health*).mp,kw. | 2152 | Advanced |  |  |  |
| 244 | (smart* adj3 ehealth*).mp,kw. | 142 | Advanced |  |  |  |
| 245 | (smart* adj3 mhealth*).mp,kw. | 572 | Advanced |  |  |  |
| 246 | (smart* adj3 phealth*).mp,kw. | 1 | Advanced |  |  |  |
| 247 | (smart* adj3 uhealth*).mp,kw. | 0 | Advanced |  |  |  |
| 248 | (smart* adj3 (count or counts or counter?)).mp,kw. | 59 | Advanced |  |  |  |
| 249 | (smart* adj3 logger?).mp,kw. | 7 | Advanced |  |  |  |
| 250 | (smart* adj3 wear*).mp,kw. | 1375 | Advanced |  |  |  |
| 251 | (smart* adj3 worn*).mp,kw. | 85 | Advanced |  |  |  |
| 252 | smartband?.mp,kw. | 21 | Advanced |  |  |  |
| 253 | (smart* adj3 band?).mp,kw. | 82 | Advanced |  |  |  |
| 254 | (smart* adj3 armband?).mp,kw. | 4 | Advanced |  |  |  |
| 255 | smartglass*.mp,kw. | 40 | Advanced |  |  |  |
| 256 | (smart* adj3 glass*).mp,kw. | 344 | Advanced |  |  |  |
| 257 | (smart* adj3 (eyewear? or eye-wear?)).mp,kw. | 5 | Advanced |  |  |  |
| 258 | smartring?.mp,kw. | 0 | Advanced |  |  |  |
| 259 | (smart* adj3 ring?).mp,kw. | 26 | Advanced |  |  |  |
| 260 | smartwatch*.mp,kw. | 756 | Advanced |  |  |  |
| 261 | (smart* adj3 watch*).mp,kw. | 587 | Advanced |  |  |  |
| 262 | (smart* adj3 cloth*).mp,kw. | 131 | Advanced |  |  |  |
| 263 | (smart* adj3 fabric?).mp,kw. | 93 | Advanced |  |  |  |
| 264 | (smart* adj3 apparel?).mp,kw. | 4 | Advanced |  |  |  |
| 265 | (smart* adj3 garment?).mp,kw. | 103 | Advanced |  |  |  |
| 266 | (electronic* adj3 textile?).mp,kw. | 369 | Advanced |  |  |  |
| 267 | (e-textile? or etextile?).mp,kw. | 179 | Advanced |  |  |  |
| 268 | (smart* adj3 textile?).mp,kw. | 380 | Advanced |  |  |  |
| 269 | (smart* adj3 cap?).mp,kw. | 31 | Advanced |  |  |  |
| 270 | (smart* adj3 dress*).mp,kw. | 87 | Advanced |  |  |  |
| 271 | (smart* adj3 glove?).mp,kw. | 54 | Advanced |  |  |  |
| 272 | (smart* adj3 hat?).mp,kw. | 4 | Advanced |  |  |  |
| 273 | (smart* adj3 helmet?).mp,kw. | 9 | Advanced |  |  |  |
| 274 | (smart* adj3 jacket?).mp,kw. | 8 | Advanced |  |  |  |
| 275 | (smart* adj3 insoles*).mp,kw. | 20 | Advanced |  |  |  |
| 276 | (smart* adj3 pant?).mp,kw. | 4 | Advanced |  |  |  |
| 277 | (smart* adj3 scarf?).mp,kw. | 0 | Advanced |  |  |  |
| 278 | (smart* adj3 shirt?).mp,kw. | 58 | Advanced |  |  |  |
| 279 | (smart* adj3 footwear?).mp,kw. | 5 | Advanced |  |  |  |
| 280 | (smart* adj3 shoe?).mp,kw. | 60 | Advanced |  |  |  |
| 281 | (smart* adj3 sock?).mp,kw. | 35 | Advanced |  |  |  |
| 282 | (smart* adj3 strap?).mp,kw. | 6 | Advanced |  |  |  |
| 283 | (smart* adj3 suit?).mp,kw. | 48 | Advanced |  |  |  |
| 284 | (smart* adj3 vest?).mp,kw. | 8 | Advanced |  |  |  |
| 285 | (smart* adj3 societ*).mp,kw. | 45 | Advanced |  |  |  |
| 286 | (intelligent adj3 sensor?).mp,kw. | 386 | Advanced |  |  |  |
| 287 | (intelligen* adj3 biosensor?).mp,kw. | 44 | Advanced |  |  |  |
| 288 | (intelligen* adj3 wear*).mp,kw. | 181 | Advanced |  |  |  |
| 289 | (intelligen* adj3 worn*).mp,kw. | 2 | Advanced |  |  |  |
| 290 | (intelligen* adj3 band?).mp,kw. | 21 | Advanced |  |  |  |
| 291 | (intelligen* adj3 armband?).mp,kw. | 1 | Advanced |  |  |  |
| 292 | (intelligen* adj3 glass*).mp,kw. | 8 | Advanced |  |  |  |
| 293 | (intelligen* adj3 (eyewear? or eye-wear?)).mp,kw. | 0 | Advanced |  |  |  |
| 294 | (intelligen* adj3 ring?).mp,kw. | 3 | Advanced |  |  |  |
| 295 | (intelligen* adj3 watch*).mp,kw. | 13 | Advanced |  |  |  |
| 296 | (intelligen* adj3 cloth*).mp,kw. | 28 | Advanced |  |  |  |
| 297 | (intelligen* adj3 fabric?).mp,kw. | 15 | Advanced |  |  |  |
| 298 | (intelligen* adj3 apparel?).mp,kw. | 2 | Advanced |  |  |  |
| 299 | (intelligen* adj3 garment?).mp,kw. | 9 | Advanced |  |  |  |
| 300 | (intelligen* adj3 textile?).mp,kw. | 39 | Advanced |  |  |  |
| 301 | (intelligen* adj3 belt?).mp,kw. | 6 | Advanced |  |  |  |
| 302 | (intelligen* adj3 cap?).mp,kw. | 2 | Advanced |  |  |  |
| 303 | (intelligen* adj3 dress*).mp,kw. | 19 | Advanced |  |  |  |
| 304 | (intelligen* adj3 glove?).mp,kw. | 5 | Advanced |  |  |  |
| 305 | (intelligen* adj3 hat?).mp,kw. | 1 | Advanced |  |  |  |
| 306 | (intelligen* adj3 helmet?).mp,kw. | 1 | Advanced |  |  |  |
| 307 | (intelligen* adj3 jacket?).mp,kw. | 0 | Advanced |  |  |  |
| 308 | (intelligen* adj3 insoles*).mp,kw. | 4 | Advanced |  |  |  |
| 309 | (intelligen* adj3 pant?).mp,kw. | 0 | Advanced |  |  |  |
| 310 | (intelligen* adj3 scarf?).mp,kw. | 0 | Advanced |  |  |  |
| 311 | (intelligen* adj3 shirt?).mp,kw. | 0 | Advanced |  |  |  |
| 312 | (intelligen* adj3 footwear?).mp,kw. | 5 | Advanced |  |  |  |
| 313 | (intelligen* adj3 shoe?).mp,kw. | 6 | Advanced |  |  |  |
| 314 | (intelligen* adj3 sock?).mp,kw. | 0 | Advanced |  |  |  |
| 315 | (intelligen* adj3 strap?).mp,kw. | 0 | Advanced |  |  |  |
| 316 | (intelligen* adj3 suit?).mp,kw. | 6 | Advanced |  |  |  |
| 317 | (intelligen* adj3 vest?).mp,kw. | 1 | Advanced |  |  |  |
| 318 | I-wear*.mp,kw. | 63 | Advanced |  |  |  |
| 319 | (ambient* adj3 intelligen*).mp,kw. | 209 | Advanced |  |  |  |
| 320 | (ambient* adj3 wear*).mp,kw. | 79 | Advanced |  |  |  |
| 321 | (ambient* adj3 worn*).mp,kw. | 7 | Advanced |  |  |  |
| 322 | sensor?-wear*.mp,kw. | 198 | Advanced |  |  |  |
| 323 | sens*wear*.mp,kw. | 1184 | Advanced |  |  |  |
| 324 | (wireless* adj3 wear*).mp,kw. | 767 | Advanced |  |  |  |
| 325 | (wireless* adj3 worn*).mp,kw. | 72 | Advanced |  |  |  |
| 326 | (wireless* adj3 biosensor*).mp,kw. | 112 | Advanced |  |  |  |
| 327 | (wireless* adj3 textile?).mp,kw. | 15 | Advanced |  |  |  |
| 328 | (wireless body adj3 area network?).mp,kw. | 387 | Advanced |  |  |  |
| 329 | (wireless sensor? adj3 network-based).mp,kw. | 34 | Advanced |  |  |  |
| 330 | WBAN?.mp,kw. | 334 | Advanced |  |  |  |
| 331 | ankle?-worn*.mp,kw. | 77 | Advanced |  |  |  |
| 332 | ankle?-mount*.mp,kw. | 43 | Advanced |  |  |  |
| 333 | finger?-worn*.mp,kw. | 22 | Advanced |  |  |  |
| 334 | waist?-worn*.mp,kw. | 229 | Advanced |  |  |  |
| 335 | waist?-mount*.mp,kw. | 123 | Advanced |  |  |  |
| 336 | wrist?-worn*.mp,kw. | 1595 | Advanced |  |  |  |
| 337 | wrist?-mount*.mp,kw. | 119 | Advanced |  |  |  |
| 338 | pedometer?.mp,kw. | 4518 | Advanced |  |  |  |
| 339 | step?count*.mp,kw. | 257 | Advanced |  |  |  |
| 340 | (step? adj3 count*).mp,kw. | 4655 | Advanced |  |  |  |
| 341 | (adidas adj3 watch*).mp,kw. | 0 | Advanced |  |  |  |
| 342 | (apple adj3 watch*).mp,kw. | 329 | Advanced |  |  |  |
| 343 | (boat adj3 watch*).mp,kw. | 4 | Advanced |  |  |  |
| 344 | (casio adj3 watch*).mp,kw. | 3 | Advanced |  |  |  |
| 345 | amazfit?.mp,kw. | 8 | Advanced |  |  |  |
| 346 | fitbit?.mp,kw. | 1565 | Advanced |  |  |  |
| 347 | (fossil adj3 watch*).mp,kw. | 0 | Advanced |  |  |  |
| 348 | garmin?.mp,kw. | 325 | Advanced |  |  |  |
| 349 | (hexoskin adj3 watch*).mp,kw. | 0 | Advanced |  |  |  |
| 350 | (huawei adj3 watch*).mp,kw. | 9 | Advanced |  |  |  |
| 351 | (jawbone adj3 watch*).mp,kw. | 1 | Advanced |  |  |  |
| 352 | (lg adj3 watch*).mp,kw. | 4 | Advanced |  |  |  |
| 353 | misfit?.mp,kw. | 1688 | Advanced |  |  |  |
| 354 | (mi adj3 watch*).mp,kw. | 5 | Advanced |  |  |  |
| 355 | (nike adj3 watch*).mp,kw. | 1 | Advanced |  |  |  |
| 356 | (puma adj3 watch*).mp,kw. | 0 | Advanced |  |  |  |
| 357 | (samsung adj3 watch*).mp,kw. | 7 | Advanced |  |  |  |
| 358 | (xiaomi adj3 watch*).mp,kw. | 0 | Advanced |  |  |  |
| 359 | whoop.mp,kw. | 142 | Advanced |  |  |  |
| 360 | (consumer? adj5 (base? or level? or grade?) adj5 device?).mp,kw. | 129 | Advanced |  |  |  |
| 361 | (consumer? adj5 (base? or level? or grade?) adj5 measur*).mp,kw. | 97 | Advanced |  |  |  |
| 362 | (consumer? adj5 (base? or level? or grade?) adj5 monitor*).mp,kw. | 92 | Advanced |  |  |  |
| 363 | (consumer? adj5 (base? or level? or grade?) adj5 tracker?).mp,kw. | 54 | Advanced |  |  |  |
| 364 | (consumer? adj5 activit* adj5 device?).mp,kw. | 22 | Advanced |  |  |  |
| 365 | (consumer? adj5 activit* adj5 measur*).mp,kw. | 24 | Advanced |  |  |  |
| 366 | (consumer? adj5 activit* adj5 monitor*).mp,kw. | 71 | Advanced |  |  |  |
| 367 | (consumer? adj5 activit* adj5 tracker?).mp,kw. | 55 | Advanced |  |  |  |
| 368 | (consumer? adj5 PAM?).mp,kw. | 7 | Advanced |  |  |  |
| 369 | (consumer? adj5 IMU?).mp,kw. | 6 | Advanced |  |  |  |
| 370 | or/186-369 | 129409 | Advanced |  |  |  |
| 371 | 185 and 370 | 8366 | Advanced |  |  |  |
| 372 | (exp animals/ or exp animal experimentation/ or nonhuman/) not ((exp animals/ or exp animal experimentation/ or nonhuman/) and exp human/) | 7643766 | Advanced |  |  |  |
| 373 | 371 not 372 | 7472 | Advanced |  |  |  |
| 374 | limit 373 to (books or chapter or conference abstract or conference paper or "conference review") | 2455 | Advanced |  |  |  |
| 375 | 373 not 374 | 5017 | Advanced |  |  |  |
| 376 | clinical study/ | 171628 | Advanced |  |  |  |
| 377 | clinical protocol/ | 107906 | Advanced |  |  |  |
| 378 | exp "clinical trial (topic)"/ | 381972 | Advanced |  |  |  |
| 379 | exp clinical trial/ | 1693275 | Advanced |  |  |  |
| 380 | controlled study/ | 8663658 | Advanced |  |  |  |
| 381 | exp "controlled clinical trial (topic)"/ | 229160 | Advanced |  |  |  |
| 382 | exp controlled clinical trial/ | 889546 | Advanced |  |  |  |
| 383 | exp "randomized controlled trial (topic)"/ | 220666 | Advanced |  |  |  |
| 384 | exp randomized controlled trial/ | 700059 | Advanced |  |  |  |
| 385 | exp randomization/ | 93605 | Advanced |  |  |  |
| 386 | pragmatic trial/ | 1500 | Advanced |  |  |  |
| 387 | superiority trial/ | 620 | Advanced |  |  |  |
| 388 | non-inferiority trial/ | 1421 | Advanced |  |  |  |
| 389 | "adaptive clinical trial (topic)"/ | 86 | Advanced |  |  |  |
| 390 | adaptive clinical trial/ | 258 | Advanced |  |  |  |
| 391 | "equivalence trial (topic)"/ | 142 | Advanced |  |  |  |
| 392 | equivalence trial/ | 127 | Advanced |  |  |  |
| 393 | "multicenter study (topic)"/ | 35710 | Advanced |  |  |  |
| 394 | multicenter study/ | 315178 | Advanced |  |  |  |
| 395 | placebo/ | 387581 | Advanced |  |  |  |
| 396 | double blind procedure/ | 194935 | Advanced |  |  |  |
| 397 | single blind procedure/ | 45231 | Advanced |  |  |  |
| 398 | exp comparative study/ | 1576779 | Advanced |  |  |  |
| 399 | feasibility study/ | 160343 | Advanced |  |  |  |
| 400 | quasi experimental study/ | 9115 | Advanced |  |  |  |
| 401 | control group/ | 122506 | Advanced |  |  |  |
| 402 | pretest posttest control group design/ | 579 | Advanced |  |  |  |
| 403 | static group comparison/ | 29 | Advanced |  |  |  |
| 404 | random sample/ | 14557 | Advanced |  |  |  |
| 405 | exp randomization/ | 93605 | Advanced |  |  |  |
| 406 | random*.mp,kw. | 2013948 | Advanced |  |  |  |
| 407 | nonrandom*.mp,kw. | 24429 | Advanced |  |  |  |
| 408 | quasirandom*.mp,kw. | 246 | Advanced |  |  |  |
| 409 | ((quasiexperimental or quasi-experimental) adj4 (study or studies)).mp,kw. | 16047 | Advanced |  |  |  |
| 410 | (control* adj4 (study or studies or group*)).mp,kw. | 9221238 | Advanced |  |  |  |
| 411 | (allocate* adj4 (study or studies or group*)).mp,kw. | 30458 | Advanced |  |  |  |
| 412 | (pragmatic* adj4 (study or studies)).mp,kw. | 2833 | Advanced |  |  |  |
| 413 | (equivalence adj4 (study or studies)).mp,kw. | 1611 | Advanced |  |  |  |
| 414 | (superiority adj4 (study or studies)).mp,kw. | 3084 | Advanced |  |  |  |
| 415 | ((noninferiority or non-inferiority) adj4 (study or studies)).mp,kw. | 3324 | Advanced |  |  |  |
| 416 | (pivotal* adj4 (study or studies)).mp,kw. | 6197 | Advanced |  |  |  |
| 417 | (comparative adj4 (study or studies)).mp,kw. | 1085352 | Advanced |  |  |  |
| 418 | (comparative adj2 effective*).mp,kw. | 121040 | Advanced |  |  |  |
| 419 | ((open label or open-label) adj4 (study or studies)).mp,kw. | 46469 | Advanced |  |  |  |
| 420 | (closed label or closed-label).mp,kw. | 21 | Advanced |  |  |  |
| 421 | (conceal* adj2 allocat*).mp,kw. | 4127 | Advanced |  |  |  |
| 422 | (intention* adj2 treat* adj2 analys?s*).mp,kw. | 32852 | Advanced |  |  |  |
| 423 | feasibility.mp,kw. | 332323 | Advanced |  |  |  |
| 424 | trial?.mp,kw. | 2785978 | Advanced |  |  |  |
| 425 | phase?.mp,kw. | 1726816 | Advanced |  |  |  |
| 426 | multicent?r*.mp,kw. | 479672 | Advanced |  |  |  |
| 427 | multi-cent?r*.mp,kw. | 53414 | Advanced |  |  |  |
| 428 | "single blind*".mp,kw. | 57226 | Advanced |  |  |  |
| 429 | "single mask*".mp,kw. | 637 | Advanced |  |  |  |
| 430 | "single dumm*".mp,kw. | 24 | Advanced |  |  |  |
| 431 | "double blind*".mp,kw. | 284951 | Advanced |  |  |  |
| 432 | "double mask*".mp,kw. | 4315 | Advanced |  |  |  |
| 433 | "double dumm*".mp,kw. | 3712 | Advanced |  |  |  |
| 434 | "triple-blind*".mp,kw. | 1599 | Advanced |  |  |  |
| 435 | "triple mask*".mp,kw. | 94 | Advanced |  |  |  |
| 436 | "triple dumm*".mp,kw. | 68 | Advanced |  |  |  |
| 437 | "treble blind*".mp,kw. | 0 | Advanced |  |  |  |
| 438 | "treble mask*".mp,kw. | 0 | Advanced |  |  |  |
| 439 | "treble dumm*".mp,kw. | 0 | Advanced |  |  |  |
| 440 | placebo*.mp,kw. | 500271 | Advanced |  |  |  |
| 441 | or/376-440 | 13438402 | Advanced |  |  |  |
| 442 | 375 and 441 | 2760 | Advanced |  |  |  |

CINAHL from EBSCOhost 1987 to February 17, 2022

| **#** | **Query** | **Limiters/Expanders** | **Last Run Via** | **Results** |
| --- | --- | --- | --- | --- |
| S294 | S248 AND S293 | Expanders - Apply equivalent subjects Search modes - Boolean/Phrase; Academic Journals | Interface - EBSCOhost Research Databases Search Screen - Advanced Search Database - CINAHL Complete | 419 |
| S293 | S249 OR S250 OR S251 OR S252 OR S253 OR S254 OR S255 OR S256 OR S257 OR S258 OR S259 OR S260 OR S261 OR S262 OR S263 OR S264 OR S265 OR S266 OR S267 OR S268 OR S269 OR S270 OR S271 OR S272 OR S273 OR S274 OR S275 OR S276 OR S277 OR S278 OR S279 OR S280 OR S281 OR S282 OR S283 OR S284 OR S285 OR S286 OR S287 OR S288 OR S289 OR S290 OR S291 OR S292 | Expanders - Apply equivalent subjects Search modes - Boolean/Phrase | Interface - EBSCOhost Research Databases Search Screen - Advanced Search Database - CINAHL Complete | 884,922 |
| S292 | TI (allocate* N4 (study or studies or group*)) OR AB (allocate* N4 (study or studies or group*)) | Expanders - Apply equivalent subjects Search modes - Boolean/Phrase | Interface - EBSCOhost Research Databases Search Screen - Advanced Search Database - CINAHL Complete | 8,034 |
| S291 | TI (control* N4 (study or studies or group*)) OR AB (control* N4 (study or studies or group*)) | Expanders - Apply equivalent subjects Search modes - Boolean/Phrase | Interface - EBSCOhost Research Databases Search Screen - Advanced Search Database - CINAHL Complete | 237,798 |
| S290 | TI ((quasiexperimental or quasi-experimental) N4 (study or studies)) OR AB ((quasiexperimental or quasi-experimental) N4 (study or studies)) | Expanders - Apply equivalent subjects Search modes - Boolean/Phrase | Interface - EBSCOhost Research Databases Search Screen - Advanced Search Database - CINAHL Complete | 7,691 |
| S289 | (MH "Quasi-Experimental Studies") | Expanders - Apply equivalent subjects Search modes - Boolean/Phrase | Interface - EBSCOhost Research Databases Search Screen - Advanced Search Database - CINAHL Complete | 14,609 |
| S288 | (MH "Nonequivalent Control Group") | Expanders - Apply equivalent subjects Search modes - Boolean/Phrase | Interface - EBSCOhost Research Databases Search Screen - Advanced Search Database - CINAHL Complete | 349 |
| S287 | (MH "Pretest-Posttest Control Group Design") | Expanders - Apply equivalent subjects Search modes - Boolean/Phrase | Interface - EBSCOhost Research Databases Search Screen - Advanced Search Database - CINAHL Complete | 1,145 |
| S286 | (MH "Control Group") | Expanders - Apply equivalent subjects Search modes - Boolean/Phrase | Interface - EBSCOhost Research Databases Search Screen - Advanced Search Database - CINAHL Complete | 12,869 |
| S285 | TI "treble mask*" OR AB "treble mask*" | Expanders - Apply equivalent subjects Search modes - Boolean/Phrase | Interface - EBSCOhost Research Databases Search Screen - Advanced Search Database - CINAHL Complete | 0 |
| S284 | TI "double mask*" OR AB "double mask*" | Expanders - Apply equivalent subjects Search modes - Boolean/Phrase | Interface - EBSCOhost Research Databases Search Screen - Advanced Search Database - CINAHL Complete | 686 |
| S283 | TI "single mask*" OR AB "single mask*" | Expanders - Apply equivalent subjects Search modes - Boolean/Phrase | Interface - EBSCOhost Research Databases Search Screen - Advanced Search Database - CINAHL Complete | 125 |
| S282 | TI "treble blind*" OR AB "treble blind*" | Limiters - Publication Type: Meta Analysis Expanders - Apply equivalent subjects Search modes - Boolean/Phrase | Interface - EBSCOhost Research Databases Search Screen - Advanced Search Database - CINAHL Complete | 0 |
| S281 | TI "triple-blind*" OR AB "triple-blind*" | Limiters - Publication Type: Meta Analysis Expanders - Apply equivalent subjects Search modes - Boolean/Phrase | Interface - EBSCOhost Research Databases Search Screen - Advanced Search Database - CINAHL Complete | 1 |
| S280 | TI "double blind*" OR AB "double blind*" | Limiters - Publication Type: Meta Analysis Expanders - Apply equivalent subjects Search modes - Boolean/Phrase | Interface - EBSCOhost Research Databases Search Screen - Advanced Search Database - CINAHL Complete | 467 |
| S279 | TI "single blind*" OR AB "single blind*" | Limiters - Publication Type: Meta Analysis Expanders - Apply equivalent subjects Search modes - Boolean/Phrase | Interface - EBSCOhost Research Databases Search Screen - Advanced Search Database - CINAHL Complete | 15 |
| S278 | TI multi-cent#r* OR AB multi-cent#r* | Limiters - Publication Type: Meta Analysis Expanders - Apply equivalent subjects Search modes - Boolean/Phrase | Interface - EBSCOhost Research Databases Search Screen - Advanced Search Database - CINAHL Complete | 105 |
| S277 | TI multicent#r* OR AB multicent#r* | Limiters - Publication Type: Meta Analysis Expanders - Apply equivalent subjects Search modes - Boolean/Phrase | Interface - EBSCOhost Research Databases Search Screen - Advanced Search Database - CINAHL Complete | 409 |
| S276 | TI phase OR AB phase | Limiters - Publication Type: Meta Analysis Expanders - Apply equivalent subjects Search modes - Boolean/Phrase | Interface - EBSCOhost Research Databases Search Screen - Advanced Search Database - CINAHL Complete | 960 |
| S275 | TI trial OR AB trial | Limiters - Publication Type: Meta Analysis Expanders - Apply equivalent subjects Search modes - Boolean/Phrase | Interface - EBSCOhost Research Databases Search Screen - Advanced Search Database - CINAHL Complete | 18,396 |
| S274 | TI (intention* N2 treat* N2 analys#s*) OR AB (intention* N2 treat* N2 analys#s*) | Limiters - Publication Type: Meta Analysis Expanders - Apply equivalent subjects Search modes - Boolean/Phrase | Interface - EBSCOhost Research Databases Search Screen - Advanced Search Database - CINAHL Complete | 108 |
| S273 | TI (conceal* N2 allocat*) OR AB (conceal* N2 allocat*) | Limiters - Publication Type: Meta Analysis Expanders - Apply equivalent subjects Search modes - Boolean/Phrase | Interface - EBSCOhost Research Databases Search Screen - Advanced Search Database - CINAHL Complete | 159 |
| S272 | TI feasibility OR AB feasibility | Limiters - Publication Type: Meta Analysis Expanders - Apply equivalent subjects Search modes - Boolean/Phrase | Interface - EBSCOhost Research Databases Search Screen - Advanced Search Database - CINAHL Complete | 217 |
| S271 | TI (comparative* N2 effective*) OR AB (comparative* N2 effective*) | Limiters - Publication Type: Meta Analysis Expanders - Apply equivalent subjects Search modes - Boolean/Phrase | Interface - EBSCOhost Research Databases Search Screen - Advanced Search Database - CINAHL Complete | 245 |
| S270 | TI (comparative* N4 (study or studies)) OR AB (comparative* N4 (study or studies)) | Limiters - Publication Type: Meta Analysis Expanders - Apply equivalent subjects Search modes - Boolean/Phrase | Interface - EBSCOhost Research Databases Search Screen - Advanced Search Database - CINAHL Complete | 676 |
| S269 | TI (pivotal* N4 (study or studies)) OR AB (pivotal* N4 (study or studies)) | Limiters - Publication Type: Meta Analysis Expanders - Apply equivalent subjects Search modes - Boolean/Phrase | Interface - EBSCOhost Research Databases Search Screen - Advanced Search Database - CINAHL Complete | 14 |
| S268 | ( TI (equivalence* N4 (study or studies)) ) OR ( AB (equivalence* N4 (study or studies)) ) | Limiters - Publication Type: Meta Analysis Expanders - Apply equivalent subjects Search modes - Boolean/Phrase | Interface - EBSCOhost Research Databases Search Screen - Advanced Search Database - CINAHL Complete | 8 |
| S267 | ( TI ((noninferiority or non-inferiority) N4 (study or studies)) ) OR ( AB ((noninferiority or non-inferiority) N4 (study or studies)) ) | Limiters - Publication Type: Meta Analysis Expanders - Apply equivalent subjects Search modes - Boolean/Phrase | Interface - EBSCOhost Research Databases Search Screen - Advanced Search Database - CINAHL Complete | 9 |
| S266 | TI (superiority N4 (study or studies)) OR AB (superiority N4 (study or studies)) | Limiters - Publication Type: Meta Analysis Expanders - Apply equivalent subjects Search modes - Boolean/Phrase | Interface - EBSCOhost Research Databases Search Screen - Advanced Search Database - CINAHL Complete | 26 |
| S265 | TI (pragmatic* N4 (study or studies)) OR AB (pragmatic* N4 (study or studies)) | Limiters - Publication Type: Meta Analysis Expanders - Apply equivalent subjects Search modes - Boolean/Phrase | Interface - EBSCOhost Research Databases Search Screen - Advanced Search Database - CINAHL Complete | 12 |
| S264 | TI quasirandom* OR AB quasirandom* | Limiters - Publication Type: Meta Analysis Expanders - Apply equivalent subjects Search modes - Boolean/Phrase | Interface - EBSCOhost Research Databases Search Screen - Advanced Search Database - CINAHL Complete | 7 |
| S263 | TI random* OR AB random* | Limiters - Publication Type: Meta Analysis Expanders - Apply equivalent subjects Search modes - Boolean/Phrase | Interface - EBSCOhost Research Databases Search Screen - Advanced Search Database - CINAHL Complete | 22,763 |
| S262 | PT randomized controlled trial | Limiters - Publication Type: Meta Analysis Expanders - Apply equivalent subjects Search modes - Boolean/Phrase | Interface - EBSCOhost Research Databases Search Screen - Advanced Search Database - CINAHL Complete | 108 |
| S261 | PT clinical trial | Expanders - Apply equivalent subjects Search modes - Boolean/Phrase | Interface - EBSCOhost Research Databases Search Screen - Advanced Search Database - CINAHL Complete | 112,011 |
| S260 | (MH "Placebos") | Expanders - Apply equivalent subjects Search modes - Boolean/Phrase | Interface - EBSCOhost Research Databases Search Screen - Advanced Search Database - CINAHL Complete | 13,613 |
| S259 | (MH "Equivalence Trials") | Expanders - Apply equivalent subjects Search modes - Boolean/Phrase | Interface - EBSCOhost Research Databases Search Screen - Advanced Search Database - CINAHL Complete | 57 |
| S258 | (MH "Preventive Trials") | Expanders - Apply equivalent subjects Search modes - Boolean/Phrase | Interface - EBSCOhost Research Databases Search Screen - Advanced Search Database - CINAHL Complete | 216 |
| S257 | (MH "Therapeutic Trials") | Expanders - Apply equivalent subjects Search modes - Boolean/Phrase | Interface - EBSCOhost Research Databases Search Screen - Advanced Search Database - CINAHL Complete | 190 |
| S256 | (MH "Intervention Trials") | Expanders - Apply equivalent subjects Search modes - Boolean/Phrase | Interface - EBSCOhost Research Databases Search Screen - Advanced Search Database - CINAHL Complete | 7,752 |
| S255 | (MH "Triple-Blind Studies") | Expanders - Apply equivalent subjects Search modes - Boolean/Phrase | Interface - EBSCOhost Research Databases Search Screen - Advanced Search Database - CINAHL Complete | 205 |
| S254 | (MH "Double-Blind Studies") | Expanders - Apply equivalent subjects Search modes - Boolean/Phrase | Interface - EBSCOhost Research Databases Search Screen - Advanced Search Database - CINAHL Complete | 52,642 |
| S253 | (MH "Single-Blind Studies") | Expanders - Apply equivalent subjects Search modes - Boolean/Phrase | Interface - EBSCOhost Research Databases Search Screen - Advanced Search Database - CINAHL Complete | 15,455 |
| S252 | (MH "Comparative Studies") | Expanders - Apply equivalent subjects Search modes - Boolean/Phrase | Interface - EBSCOhost Research Databases Search Screen - Advanced Search Database - CINAHL Complete | 431,684 |
| S251 | (MH "Multicenter Studies") | Expanders - Apply equivalent subjects Search modes - Boolean/Phrase | Interface - EBSCOhost Research Databases Search Screen - Advanced Search Database - CINAHL Complete | 338,198 |
| S250 | (MH "Randomized Controlled Trials+") | Expanders - Apply equivalent subjects Search modes - Boolean/Phrase | Interface - EBSCOhost Research Databases Search Screen - Advanced Search Database - CINAHL Complete | 126,791 |
| S249 | (MH "Clinical Trials+") | Expanders - Apply equivalent subjects Search modes - Boolean/Phrase | Interface - EBSCOhost Research Databases Search Screen - Advanced Search Database - CINAHL Complete | 334,605 |
| S248 | S165 AND S247 | Expanders - Apply equivalent subjects Search modes - Boolean/Phrase | Interface - EBSCOhost Research Databases Search Screen - Advanced Search Database - CINAHL Complete | 1,386 |
| S247 | S166 OR S167 OR S168 OR S169 OR S170 OR S171 OR S172 OR S173 OR S174 OR S175 OR S176 OR S177 OR S178 OR S179 OR S180 OR S181 OR S182 OR S183 OR S184 OR S185 OR S186 OR S187 OR S188 OR S189 OR S190 OR S191 OR S192 OR S193 OR S194 OR S195 OR S196 OR S197 OR S198 OR S199 OR S200 OR S201 OR S202 OR S203 OR S204 OR S205 OR S206 OR S207 OR S208 OR S209 OR S210 OR S211 OR S212 OR S213 OR S214 OR S215 OR S216 OR S217 OR S218 OR S219 OR S220 OR S221 OR S222 OR S223 OR S224 OR S225 OR S226 OR S227 OR S228 OR S229 OR S230 OR S231 OR S232 OR S233 OR S234 OR S235 OR S236 OR S237 OR S238 OR S239 OR S240 OR S241 OR S242 OR S243 OR S244 OR S245 OR S246 | Expanders - Apply equivalent subjects Search modes - Boolean/Phrase | Interface - EBSCOhost Research Databases Search Screen - Advanced Search Database - CINAHL Complete | 29,256 |
| S246 | TI (consumer N5 (PAM or IMU)) OR AB (consumer N5 (PAM or IMU)) | Expanders - Apply equivalent subjects Search modes - Boolean/Phrase | Interface - EBSCOhost Research Databases Search Screen - Advanced Search Database - CINAHL Complete | 3 |
| S245 | TI (consumer N5 activit* N5 (device or measur* or monitor* or tracker)) OR AB (consumer N5 activit* N5 (device or measur* or monitor* or tracker)) | Expanders - Apply equivalent subjects Search modes - Boolean/Phrase | Interface - EBSCOhost Research Databases Search Screen - Advanced Search Database - CINAHL Complete | 71 |
| S244 | TI (consumer N5 (base or level or grade) N5 (device or measur* or monitor* or tracker)) OR AB (consumer N5 (base or level or grade) N5 (device or measur* or monitor* or tracker)) | Expanders - Apply equivalent subjects Search modes - Boolean/Phrase | Interface - EBSCOhost Research Databases Search Screen - Advanced Search Database - CINAHL Complete | 65 |
| S243 | IB TI ((hexoskin or huawei or jawbone or lg or mi or nike or puma or samsug or xiaomi) N3 watch*) OR AB ((hexoskin or huawei or jawbone or lg or mi or nike or puma or samsug or xiaomi) N3 watch*) | Expanders - Apply equivalent subjects Search modes - Boolean/Phrase | Interface - EBSCOhost Research Databases Search Screen - Advanced Search Database - CINAHL Complete | 8 |
| S242 | TI (amazfit or fitbit or garmin or misfit or whoop) OR AB (amazfit or fitbit or garmin or misfit or whoop) | Expanders - Apply equivalent subjects Search modes - Boolean/Phrase | Interface - EBSCOhost Research Databases Search Screen - Advanced Search Database - CINAHL Complete | 1,127 |
| S241 | TI ((adidas or apple or boat or casio or fossil) N3 watch*) OR AB ((adidas or apple or boat or casio or fossil) N3 watch*) | Expanders - Apply equivalent subjects Search modes - Boolean/Phrase | Interface - EBSCOhost Research Databases Search Screen - Advanced Search Database - CINAHL Complete | 157 |
| S240 | TI (step N3 count*) OR AB (step N3 count*) | Expanders - Apply equivalent subjects Search modes - Boolean/Phrase | Interface - EBSCOhost Research Databases Search Screen - Advanced Search Database - CINAHL Complete | 1,610 |
| S239 | TI (pedometer or stepcount*) OR AB (pedometer or stepcount*) | Expanders - Apply equivalent subjects Search modes - Boolean/Phrase | Interface - EBSCOhost Research Databases Search Screen - Advanced Search Database - CINAHL Complete | 1,781 |
| S238 | TI (ankle-worn* or ankle-mount* or finger-worn* or waist-worn* or waist-mount*) OR AB (ankle-worn* or ankle-mount* or finger-worn* or waist-worn* or waist-mount*) | Expanders - Apply equivalent subjects Search modes - Boolean/Phrase | Interface - EBSCOhost Research Databases Search Screen - Advanced Search Database - CINAHL Complete | 173 |
| S237 | TI WBAN OR AB WBAN | Expanders - Apply equivalent subjects Search modes - Boolean/Phrase | Interface - EBSCOhost Research Databases Search Screen - Advanced Search Database - CINAHL Complete | 49 |
| S236 | TI (wireless sensor N3 network-based) OR AB (wireless sensor N3 network-based) | Expanders - Apply equivalent subjects Search modes - Boolean/Phrase | Interface - EBSCOhost Research Databases Search Screen - Advanced Search Database - CINAHL Complete | 1 |
| S235 | TI (wireless body N3 area network) OR AB (wireless body N3 area network) | Expanders - Apply equivalent subjects Search modes - Boolean/Phrase | Interface - EBSCOhost Research Databases Search Screen - Advanced Search Database - CINAHL Complete | 58 |
| S234 | TI (wireless* N3 (wear* or worn* or biosensor* or textile)) OR AB (wireless* N3 (wear* or worn* or biosensor* or textile)) | Expanders - Apply equivalent subjects Search modes - Boolean/Phrase | Interface - EBSCOhost Research Databases Search Screen - Advanced Search Database - CINAHL Complete | 124 |
| S233 | TI (sensor-wear* or sens*wear*) OR AB (sensor-wear* or sens*wear*) | Expanders - Apply equivalent subjects Search modes - Boolean/Phrase | Interface - EBSCOhost Research Databases Search Screen - Advanced Search Database - CINAHL Complete | 285 |
| S232 | TI (ambient* N3 (intelligen* or wear* or worn*)) OR AB (ambient* N3 (intelligen* or wear* or worn*)) | Expanders - Apply equivalent subjects Search modes - Boolean/Phrase | Interface - EBSCOhost Research Databases Search Screen - Advanced Search Database - CINAHL Complete | 50 |
| S231 | TI I-wear* OR AB I-wear* | Expanders - Apply equivalent subjects Search modes - Boolean/Phrase | Interface - EBSCOhost Research Databases Search Screen - Advanced Search Database - CINAHL Complete | 40 |
| S230 | IB TI (intelligen* N3 (footwear or shoe or sock or strap or suit or vest)) OR AB (intelligen* N3 (footwear or shoe or sock or strap or suit or vest)) | Expanders - Apply equivalent subjects Search modes - Boolean/Phrase | Interface - EBSCOhost Research Databases Search Screen - Advanced Search Database - CINAHL Complete | 3 |
| S229 | TI (intelligen* N3 (jacket or insoles* or pant or scarf or shirt)) OR AB (intelligen* N3 (jacket or insoles* or pant or scarf or shirt)) | Expanders - Apply equivalent subjects Search modes - Boolean/Phrase | Interface - EBSCOhost Research Databases Search Screen - Advanced Search Database - CINAHL Complete | 1 |
| S228 | TI (intelligen* N3 (belt or cap or dress* or glove or hat or helmet)) OR AB (intelligen* N3 (belt or cap or dress* or glove or hat or helmet)) | Expanders - Apply equivalent subjects Search modes - Boolean/Phrase | Interface - EBSCOhost Research Databases Search Screen - Advanced Search Database - CINAHL Complete | 8 |
| S227 | TI (intelligen* N3 (cloth* or fabric or apparel or garment or textile)) OR AB (intelligen* N3 (cloth* or fabric or apparel or garment or textile)) | Expanders - Apply equivalent subjects Search modes - Boolean/Phrase | Interface - EBSCOhost Research Databases Search Screen - Advanced Search Database - CINAHL Complete | 5 |
| S226 | TI (intelligen* N3 (glass* or eyewear or eye-wear)) OR AB (intelligen* N3 (glass* or eyewear or eye-wear)) | Expanders - Apply equivalent subjects Search modes - Boolean/Phrase | Interface - EBSCOhost Research Databases Search Screen - Advanced Search Database - CINAHL Complete | 2 |
| S225 | TI (intelligen* N3 (band or armband or ring or watch*)) OR AB (intelligen* N3 (band or armband or ring or watch*)) | Expanders - Apply equivalent subjects Search modes - Boolean/Phrase | Interface - EBSCOhost Research Databases Search Screen - Advanced Search Database - CINAHL Complete | 9 |
| S224 | TI (intelligen* N3 (wear* or worn*)) OR AB (intelligen* N3 (wear* or worn*)) | Expanders - Apply equivalent subjects Search modes - Boolean/Phrase | Interface - EBSCOhost Research Databases Search Screen - Advanced Search Database - CINAHL Complete | 21 |
| S223 | TI (intelligent N3 (sensor or biosensor)) OR AB (intelligent N3 (sensor or biosensor)) | Expanders - Apply equivalent subjects Search modes - Boolean/Phrase | Interface - EBSCOhost Research Databases Search Screen - Advanced Search Database - CINAHL Complete | 24 |
| S222 | TI (smart* N3 societ*) OR AB (smart* N3 societ*) | Expanders - Apply equivalent subjects Search modes - Boolean/Phrase | Interface - EBSCOhost Research Databases Search Screen - Advanced Search Database - CINAHL Complete | 18 |
| S221 | TI (smart* N3 (footwear or shoe or sock or strap or suit or vest)) OR AB (smart* N3 (footwear or shoe or sock or strap or suit or vest)) | Expanders - Apply equivalent subjects Search modes - Boolean/Phrase | Interface - EBSCOhost Research Databases Search Screen - Advanced Search Database - CINAHL Complete | 47 |
| S220 | TI (smart* N3 (jacket or insoles* or pant or scarf or shirt)) OR AB (smart* N3 (jacket or insoles* or pant or scarf or shirt)) | Expanders - Apply equivalent subjects Search modes - Boolean/Phrase | Interface - EBSCOhost Research Databases Search Screen - Advanced Search Database - CINAHL Complete | 26 |
| S219 | TI (smart* N3 (cap or dress* or glove or hat or helmet)) OR AB (smart* N3 (cap or dress* or glove or hat or helmet)) | Expanders - Apply equivalent subjects Search modes - Boolean/Phrase | Interface - EBSCOhost Research Databases Search Screen - Advanced Search Database - CINAHL Complete | 34 |
| S218 | TI (e-textile or etextile) OR AB (e-textile or etextile) | Expanders - Apply equivalent subjects Search modes - Boolean/Phrase | Interface - EBSCOhost Research Databases Search Screen - Advanced Search Database - CINAHL Complete | 10 |
| S217 | TI (smart* N3 (cloth* or fabric or apparel or garment or textile)) OR AB (smart* N3 (cloth* or fabric or apparel or garment or textile)) | Expanders - Apply equivalent subjects Search modes - Boolean/Phrase | Interface - EBSCOhost Research Databases Search Screen - Advanced Search Database - CINAHL Complete | 61 |
| S216 | TI (smart* N3 watch*) OR AB (smart* N3 watch*) | Expanders - Apply equivalent subjects Search modes - Boolean/Phrase | Interface - EBSCOhost Research Databases Search Screen - Advanced Search Database - CINAHL Complete | 122 |
| S215 | TI smartwatch* OR AB smartwatch* | Expanders - Apply equivalent subjects Search modes - Boolean/Phrase | Interface - EBSCOhost Research Databases Search Screen - Advanced Search Database - CINAHL Complete | 235 |
| S214 | TI (smart* N3 ring) OR AB (smart* N3 ring) | Expanders - Apply equivalent subjects Search modes - Boolean/Phrase | Interface - EBSCOhost Research Databases Search Screen - Advanced Search Database - CINAHL Complete | 4 |
| S213 | TI (smart* N3 (eyewear or eye-wear)) OR AB (smart* N3 (eyewear or eye-wear)) | Expanders - Apply equivalent subjects Search modes - Boolean/Phrase | Interface - EBSCOhost Research Databases Search Screen - Advanced Search Database - CINAHL Complete | 1 |
| S212 | TI (smart* N3 glass*) OR AB (smart* N3 glass*) | Expanders - Apply equivalent subjects Search modes - Boolean/Phrase | Interface - EBSCOhost Research Databases Search Screen - Advanced Search Database - CINAHL Complete | 57 |
| S211 | TI smartglass* OR AB smartglass* | Expanders - Apply equivalent subjects Search modes - Boolean/Phrase | Interface - EBSCOhost Research Databases Search Screen - Advanced Search Database - CINAHL Complete | 9 |
| S210 | IB TI (smart* N3 (band or armband)) OR AB (smart* N3 (band or armband)) | Expanders - Apply equivalent subjects Search modes - Boolean/Phrase | Interface - EBSCOhost Research Databases Search Screen - Advanced Search Database - CINAHL Complete | 12 |
| S209 | TI smartband OR AB smartband | Expanders - Apply equivalent subjects Search modes - Boolean/Phrase | Interface - EBSCOhost Research Databases Search Screen - Advanced Search Database - CINAHL Complete | 9 |
| S208 | TI (smart* N3 (wear* or worn*)) OR AB (smart* N3 (wear* or worn*)) | Expanders - Apply equivalent subjects Search modes - Boolean/Phrase | Interface - EBSCOhost Research Databases Search Screen - Advanced Search Database - CINAHL Complete | 259 |
| S207 | TI V(smart* N3 logger) OR AB (smart* N3 logger) | Expanders - Apply equivalent subjects Search modes - Boolean/Phrase | Interface - EBSCOhost Research Databases Search Screen - Advanced Search Database - CINAHL Complete | 1 |
| S206 | TI (smart* N3 (count or counts or counter)) OR AB (smart* N3 (count or counts or counter)) | Expanders - Apply equivalent subjects Search modes - Boolean/Phrase | Interface - EBSCOhost Research Databases Search Screen - Advanced Search Database - CINAHL Complete | 24 |
| S205 | TI (smart* N3 (health* or ehealth* or mhealth* or phealth* or uhealth*)) OR AB (smart* N3 (health* or ehealth* or mhealth* or phealth* or uhealth*)) | Expanders - Apply equivalent subjects Search modes - Boolean/Phrase | Interface - EBSCOhost Research Databases Search Screen - Advanced Search Database - CINAHL Complete | 1,143 |
| S204 | TI (smart* N3 track*) OR AB (smart* N3 track*) | Expanders - Apply equivalent subjects Search modes - Boolean/Phrase | Interface - EBSCOhost Research Databases Search Screen - Advanced Search Database - CINAHL Complete | 160 |
| S203 | TI (smart* N3 (sensor or biosensor or monitor*)) OR AB (smart* N3 (sensor or biosensor or monitor*)) | Expanders - Apply equivalent subjects Search modes - Boolean/Phrase | Interface - EBSCOhost Research Databases Search Screen - Advanced Search Database - CINAHL Complete | 659 |
| S202 | TI (smart* N3 (device or technolog* or wireless*)) OR AB (smart* N3 (device or technolog* or wireless*)) | Expanders - Apply equivalent subjects Search modes - Boolean/Phrase | Interface - EBSCOhost Research Databases Search Screen - Advanced Search Database - CINAHL Complete | 2,112 |
| S201 | TI ((ehealth* or phealth* or uhealth*) N3 mobile) OR AB ((ehealth* or phealth* or uhealth*) N3 mobile) | Expanders - Apply equivalent subjects Search modes - Boolean/Phrase | Interface - EBSCOhost Research Databases Search Screen - Advanced Search Database - CINAHL Complete | 57 |
| S200 | TI (movement N3 sensor) OR AB (movement N3 sensor) | Expanders - Apply equivalent subjects Search modes - Boolean/Phrase | Interface - EBSCOhost Research Databases Search Screen - Advanced Search Database - CINAHL Complete | 165 |
| S199 | TI (movement N3 sensor) OR AB (movement N3 sensor) | Expanders - Apply equivalent subjects Search modes - Boolean/Phrase | Interface - EBSCOhost Research Databases Search Screen - Advanced Search Database - CINAHL Complete | 165 |
| S198 | TI (embedded N3 sensor) OR AB (embedded N3 sensor) | Expanders - Apply equivalent subjects Search modes - Boolean/Phrase | Interface - EBSCOhost Research Databases Search Screen - Advanced Search Database - CINAHL Complete | 134 |
| S197 | TI (remote* N3 sensing) OR AB (remote* N3 sensing) | Expanders - Apply equivalent subjects Search modes - Boolean/Phrase | Interface - EBSCOhost Research Databases Search Screen - Advanced Search Database - CINAHL Complete | 221 |
| S196 | TI ((biometric or bio-metric) N3 (mobile? or monitor* or sensor*)) OR AB ((biometric or bio-metric) N3 (mobile? or monitor* or sensor*)) | Expanders - Apply equivalent subjects Search modes - Boolean/Phrase | Interface - EBSCOhost Research Databases Search Screen - Advanced Search Database - CINAHL Complete | 46 |
| S195 | TI (etrack* or e-track*) OR AB (etrack* or e-track*) | Expanders - Apply equivalent subjects Search modes - Boolean/Phrase | Interface - EBSCOhost Research Databases Search Screen - Advanced Search Database - CINAHL Complete | 9 |
| S194 | TI (mtrack* or m-track*) OR AB (mtrack* or m-track*) | Expanders - Apply equivalent subjects Search modes - Boolean/Phrase | Interface - EBSCOhost Research Databases Search Screen - Advanced Search Database - CINAHL Complete | 20 |
| S193 | TI ((wellness* or well-ness*) N3 track*) OR AB ((wellness* or well-ness*) N3 track*) | Expanders - Apply equivalent subjects Search modes - Boolean/Phrase | Interface - EBSCOhost Research Databases Search Screen - Advanced Search Database - CINAHL Complete | 23 |
| S192 | TI ((wellness* or well-ness*) N3 device) OR AB ((wellness* or well-ness*) N3 device) | Expanders - Apply equivalent subjects Search modes - Boolean/Phrase | Interface - EBSCOhost Research Databases Search Screen - Advanced Search Database - CINAHL Complete | 17 |
| S191 | TI ((wellness* or well-ness*) N3 mobile) OR AB ((wellness* or well-ness*) N3 mobile) | Expanders - Apply equivalent subjects Search modes - Boolean/Phrase | Interface - EBSCOhost Research Databases Search Screen - Advanced Search Database - CINAHL Complete | 37 |
| S190 | TI (self N3 track*) OR AB (self N3 track*) | Expanders - Apply equivalent subjects Search modes - Boolean/Phrase | Interface - EBSCOhost Research Databases Search Screen - Advanced Search Database - CINAHL Complete | 315 |
| S189 | TI selftrack* OR AB selftrack* | Expanders - Apply equivalent subjects Search modes - Boolean/Phrase | Interface - EBSCOhost Research Databases Search Screen - Advanced Search Database - CINAHL Complete | 1 |
| S188 | TI ((selfmonitor* or self-monitor*) N3 device) OR AB ((selfmonitor* or self-monitor*) N3 device) | Expanders - Apply equivalent subjects Search modes - Boolean/Phrase | Interface - EBSCOhost Research Databases Search Screen - Advanced Search Database - CINAHL Complete | 69 |
| S187 | TI ((selfmonitor* or self-monitor*) N3 mobile) OR AB ((selfmonitor* or self-monitor*) N3 mobile) | Expanders - Apply equivalent subjects Search modes - Boolean/Phrase | Interface - EBSCOhost Research Databases Search Screen - Advanced Search Database - CINAHL Complete | 56 |
| S186 | TI (physical* N3 track*) OR AB (physical* N3 track*) | Expanders - Apply equivalent subjects Search modes - Boolean/Phrase | Interface - EBSCOhost Research Databases Search Screen - Advanced Search Database - CINAHL Complete | 361 |
| S185 | TI (physical* N3 mobile) OR AB (physical* N3 mobile) | Expanders - Apply equivalent subjects Search modes - Boolean/Phrase | Interface - EBSCOhost Research Databases Search Screen - Advanced Search Database - CINAHL Complete | 140 |
| S184 | TI (movemonitor* or move-monitor*) OR AB (movemonitor* or move-monitor*) | Expanders - Apply equivalent subjects Search modes - Boolean/Phrase | Interface - EBSCOhost Research Databases Search Screen - Advanced Search Database - CINAHL Complete | 9 |
| S183 | TI (move* N3 mobile) OR AB (move* N3 mobile) | Expanders - Apply equivalent subjects Search modes - Boolean/Phrase | Interface - EBSCOhost Research Databases Search Screen - Advanced Search Database - CINAHL Complete | 111 |
| S182 | TI (fitness* N3 monitor*) OR AB (fitness* N3 monitor*) | Expanders - Apply equivalent subjects Search modes - Boolean/Phrase | Interface - EBSCOhost Research Databases Search Screen - Advanced Search Database - CINAHL Complete | 122 |
| S181 | TI (fitness* N3 track*) OR AB (fitness* N3 track*) | Expanders - Apply equivalent subjects Search modes - Boolean/Phrase | Interface - EBSCOhost Research Databases Search Screen - Advanced Search Database - CINAHL Complete | 283 |
| S180 | TI (fitness* N3 device) OR AB (fitness* N3 device) | Expanders - Apply equivalent subjects Search modes - Boolean/Phrase | Interface - EBSCOhost Research Databases Search Screen - Advanced Search Database - CINAHL Complete | 76 |
| S179 | TI (fitness* N3 band) OR AB (fitness* N3 band) | Expanders - Apply equivalent subjects Search modes - Boolean/Phrase | Interface - EBSCOhost Research Databases Search Screen - Advanced Search Database - CINAHL Complete | 22 |
| S178 | TI (fitness* N3 mobile) OR AB (fitness* N3 mobile) | Expanders - Apply equivalent subjects Search modes - Boolean/Phrase | Interface - EBSCOhost Research Databases Search Screen - Advanced Search Database - CINAHL Complete | 54 |
| S177 | TI (activit* N3 monitor*) OR AB (activit* N3 monitor*) | Expanders - Apply equivalent subjects Search modes - Boolean/Phrase | Interface - EBSCOhost Research Databases Search Screen - Advanced Search Database - CINAHL Complete | 4,343 |
| S176 | TI (activit* N3 track*) OR AB (activit* N3 track*) | Expanders - Apply equivalent subjects Search modes - Boolean/Phrase | Interface - EBSCOhost Research Databases Search Screen - Advanced Search Database - CINAHL Complete | 1,010 |
| S175 | TI (activit* N3 mobile) OR AB (activit* N3 mobile) | Expanders - Apply equivalent subjects Search modes - Boolean/Phrase | Interface - EBSCOhost Research Databases Search Screen - Advanced Search Database - CINAHL Complete | 173 |
| S174 | TI actigraph* OR AB actigraph* | Expanders - Apply equivalent subjects Search modes - Boolean/Phrase | Interface - EBSCOhost Research Databases Search Screen - Advanced Search Database - CINAHL Complete | 3,575 |
| S173 | TI acceleromet* OR AB acceleromet* | Expanders - Apply equivalent subjects Search modes - Boolean/Phrase | Interface - EBSCOhost Research Databases Search Screen - Advanced Search Database - CINAHL Complete | 8,156 |
| S172 | TI wear-able OR AB wear-able | Expanders - Apply equivalent subjects Search modes - Boolean/Phrase | Interface - EBSCOhost Research Databases Search Screen - Advanced Search Database - CINAHL Complete | 1 |
| S171 | TI wearable OR AB wearable | Expanders - Apply equivalent subjects Search modes - Boolean/Phrase | Interface - EBSCOhost Research Databases Search Screen - Advanced Search Database - CINAHL Complete | 3,793 |
| S170 | (MH "Smart Glasses") | Expanders - Apply equivalent subjects Search modes - Boolean/Phrase | Interface - EBSCOhost Research Databases Search Screen - Advanced Search Database - CINAHL Complete | 18 |
| S169 | (MH "Fitness Trackers") | Expanders - Apply equivalent subjects Search modes - Boolean/Phrase | Interface - EBSCOhost Research Databases Search Screen - Advanced Search Database - CINAHL Complete | 278 |
| S168 | (MH "Actigraphy") | Expanders - Apply equivalent subjects Search modes - Boolean/Phrase | Interface - EBSCOhost Research Databases Search Screen - Advanced Search Database - CINAHL Complete | 1,531 |
| S167 | (MH "Accelerometers") | Expanders - Apply equivalent subjects Search modes - Boolean/Phrase | Interface - EBSCOhost Research Databases Search Screen - Advanced Search Database - CINAHL Complete | 2,705 |
| S166 | (MH "Wearable Sensors+") | Expanders - Apply equivalent subjects Search modes - Boolean/Phrase | Interface - EBSCOhost Research Databases Search Screen - Advanced Search Database - CINAHL Complete | 6,317 |
| S165 | S1 OR S2 OR S3 OR S4 OR S5 OR S6 OR S7 OR S8 OR S9 OR S10 OR S11 OR S12 OR S13 OR S14 OR S15 OR S16 OR S17 OR S18 OR S19 OR S20 OR S21 OR S22 OR S23 OR S24 OR S25 OR S26 OR S27 OR S28 OR S29 OR S30 OR S31 OR S32 OR S33 OR S34 OR S35 OR S36 OR S37 OR S38 OR S39 OR S40 OR S41 OR S42 OR S43 OR S44 OR S45 OR S46 OR S47 OR S48 OR S49 OR S50 OR S51 OR S52 OR S53 OR S54 OR S55 OR S56 OR S57 OR S58 OR S59 OR S60 OR S61 OR S62 OR S63 OR S64 OR S65 OR S66 OR S67 OR S68 OR S69 OR S70 OR S71 OR S72 OR S73 OR S74 OR S75 OR S76 OR S77 OR S78 OR S79 OR S80 OR S81 OR S82 OR S83 OR S84 OR S85 OR S86 OR S87 OR S88 OR S89 OR S90 OR S91 OR S92 OR S93 OR S94 OR S95 OR S96 OR S97 OR S98 OR S99 OR S100 OR S101 OR S102 OR S103 OR S104 OR S105 OR S106 OR S107 OR S108 OR S109 OR S110 OR S111 OR S112 OR S113 OR S114 OR S115 OR S116 OR S117 OR S118 OR S119 OR S120 OR S121 OR S122 OR S123 OR S124 OR S125 OR S126 OR S127 OR S128 OR S129 OR S130 OR S131 OR S132 OR S133 OR S134 OR S135 OR S136 OR S137 OR S138 OR S139 OR S140 OR S141 OR S142 OR S143 OR S144 OR S145 OR S146 OR S147 OR S148 OR S149 OR S150 OR S151 OR S152 OR S153 OR S154 OR S155 OR S156 OR S157 OR S158 OR S159 OR S160 OR S161 OR S162 OR S163 OR S164 | Expanders - Apply equivalent subjects Search modes - Boolean/Phrase | Interface - EBSCOhost Research Databases Search Screen - Advanced Search Database - CINAHL Complete | 939,382 |
| S164 | TI wilms* OR AB wilms* | Expanders - Apply equivalent subjects Search modes - Boolean/Phrase | Interface - EBSCOhost Research Databases Search Screen - Advanced Search Database - CINAHL Complete | 1,187 |
| S163 | TI vipoma* OR AB vipoma* | Expanders - Apply equivalent subjects Search modes - Boolean/Phrase | Interface - EBSCOhost Research Databases Search Screen - Advanced Search Database - CINAHL Complete | 24 |
| S162 | TI vipoma* OR AB vipoma* | Expanders - Apply equivalent subjects Search modes - Boolean/Phrase | Interface - EBSCOhost Research Databases Search Screen - Advanced Search Database - CINAHL Complete | 24 |
| S161 | TI trophoblast* OR AB trophoblast* | Expanders - Apply equivalent subjects Search modes - Boolean/Phrase | Interface - EBSCOhost Research Databases Search Screen - Advanced Search Database - CINAHL Complete | 2,957 |
| S160 | TI thymom* OR AB thymom* | Expanders - Apply equivalent subjects Search modes - Boolean/Phrase | Interface - EBSCOhost Research Databases Search Screen - Advanced Search Database - CINAHL Complete | 1,040 |
| S159 | TI teratoma* OR AB teratoma* | Expanders - Apply equivalent subjects Search modes - Boolean/Phrase | Interface - EBSCOhost Research Databases Search Screen - Advanced Search Database - CINAHL Complete | 1,720 |
| S158 | TI teratocarcin* OR AB teratocarcin* | Expanders - Apply equivalent subjects Search modes - Boolean/Phrase | Interface - EBSCOhost Research Databases Search Screen - Advanced Search Database - CINAHL Complete | 44 |
| S157 | TI thecoma* OR AB thecoma* | Expanders - Apply equivalent subjects Search modes - Boolean/Phrase | Interface - EBSCOhost Research Databases Search Screen - Advanced Search Database - CINAHL Complete | 26 |
| S156 | TI struma ovarii* OR AB struma ovarii* | Expanders - Apply equivalent subjects Search modes - Boolean/Phrase | Interface - EBSCOhost Research Databases Search Screen - Advanced Search Database - CINAHL Complete | 69 |
| S155 | TI somato* OR AB somato* | Expanders - Apply equivalent subjects Search modes - Boolean/Phrase | Interface - EBSCOhost Research Databases Search Screen - Advanced Search Database - CINAHL Complete | 8,054 |
| S154 | TI Sertoli- Leydig OR AB Sertoli- Leydig | Expanders - Apply equivalent subjects Search modes - Boolean/Phrase | Interface - EBSCOhost Research Databases Search Screen - Advanced Search Database - CINAHL Complete | 89 |
| S153 | TI seminoma* OR AB seminoma* | Expanders - Apply equivalent subjects Search modes - Boolean/Phrase | Interface - EBSCOhost Research Databases Search Screen - Advanced Search Database - CINAHL Complete | 777 |
| S152 | TI sarcom* OR AB sarcom* | Expanders - Apply equivalent subjects Search modes - Boolean/Phrase | Interface - EBSCOhost Research Databases Search Screen - Advanced Search Database - CINAHL Complete | 13,731 |
| S151 | TI rhabdo* OR AB rhabdo* | Expanders - Apply equivalent subjects Search modes - Boolean/Phrase | Interface - EBSCOhost Research Databases Search Screen - Advanced Search Database - CINAHL Complete | 4,510 |
| S150 | TI retinoblastom* OR AB retinoblastom* | Expanders - Apply equivalent subjects Search modes - Boolean/Phrase | Interface - EBSCOhost Research Databases Search Screen - Advanced Search Database - CINAHL Complete | 1,454 |
| S149 | TI prolactinom* OR AB prolactinom* | Expanders - Apply equivalent subjects Search modes - Boolean/Phrase | Interface - EBSCOhost Research Databases Search Screen - Advanced Search Database - CINAHL Complete | 297 |
| S148 | TI polycythem* vera OR AB polycythem* vera | Expanders - Apply equivalent subjects Search modes - Boolean/Phrase | Interface - EBSCOhost Research Databases Search Screen - Advanced Search Database - CINAHL Complete | 622 |
| S147 | TI plasmacytom* OR AB plasmacytom* | Expanders - Apply equivalent subjects Search modes - Boolean/Phrase | Interface - EBSCOhost Research Databases Search Screen - Advanced Search Database - CINAHL Complete | 604 |
| S146 | TI pineocytoma* OR AB pineocytoma* | Expanders - Apply equivalent subjects Search modes - Boolean/Phrase | Interface - EBSCOhost Research Databases Search Screen - Advanced Search Database - CINAHL Complete | 7 |
| S145 | TI pineoblastoma* OR AB pineoblastoma* | Expanders - Apply equivalent subjects Search modes - Boolean/Phrase | Interface - EBSCOhost Research Databases Search Screen - Advanced Search Database - CINAHL Complete | 45 |
| S144 | TI pinealoma* OR AB pinealoma* | Expanders - Apply equivalent subjects Search modes - Boolean/Phrase | Interface - EBSCOhost Research Databases Search Screen - Advanced Search Database - CINAHL Complete | 3 |
| S143 | TI pinealocytoma* OR AB pinealocytoma* | Expanders - Apply equivalent subjects Search modes - Boolean/Phrase | Interface - EBSCOhost Research Databases Search Screen - Advanced Search Database - CINAHL Complete | 2 |
| S142 | TI phyllo#des* OR AB phyllo#des* | Expanders - Apply equivalent subjects Search modes - Boolean/Phrase | Interface - EBSCOhost Research Databases Search Screen - Advanced Search Database - CINAHL Complete | 371 |
| S141 | TI pheochromocytom* OR AB pheochromocytom* | Expanders - Apply equivalent subjects Search modes - Boolean/Phrase | Interface - EBSCOhost Research Databases Search Screen - Advanced Search Database - CINAHL Complete | 1,592 |
| S140 | TI paragangliom* OR AB paragangliom* | Expanders - Apply equivalent subjects Search modes - Boolean/Phrase | Interface - EBSCOhost Research Databases Search Screen - Advanced Search Database - CINAHL Complete | 1,197 |
| S139 | TI papillary* OR AB papillary* | Expanders - Apply equivalent subjects Search modes - Boolean/Phrase | Interface - EBSCOhost Research Databases Search Screen - Advanced Search Database - CINAHL Complete | 7,101 |
| S138 | TI papilloma* OR AB papilloma* | Expanders - Apply equivalent subjects Search modes - Boolean/Phrase | Interface - EBSCOhost Research Databases Search Screen - Advanced Search Database - CINAHL Complete | 13,432 |
| S137 | TI osteosarcom* OR AB osteosarcom* | Expanders - Apply equivalent subjects Search modes - Boolean/Phrase | Interface - EBSCOhost Research Databases Search Screen - Advanced Search Database - CINAHL Complete | 3,339 |
| S136 | TI osteoma* OR AB osteoma* | Expanders - Apply equivalent subjects Search modes - Boolean/Phrase | Interface - EBSCOhost Research Databases Search Screen - Advanced Search Database - CINAHL Complete | 1,507 |
| S135 | TI osteoclastom* OR AB osteoclastom* | Expanders - Apply equivalent subjects Search modes - Boolean/Phrase | Interface - EBSCOhost Research Databases Search Screen - Advanced Search Database - CINAHL Complete | 16 |
| S134 | TI osteochondrom* OR AB osteochondrom* | Expanders - Apply equivalent subjects Search modes - Boolean/Phrase | Interface - EBSCOhost Research Databases Search Screen - Advanced Search Database - CINAHL Complete | 669 |
| S133 | TI osteoblastom* OR AB osteoblastom* | Expanders - Apply equivalent subjects Search modes - Boolean/Phrase | Interface - EBSCOhost Research Databases Search Screen - Advanced Search Database - CINAHL Complete | 192 |
| S132 | TI oligo* OR AB oligo* | Expanders - Apply equivalent subjects Search modes - Boolean/Phrase | Interface - EBSCOhost Research Databases Search Screen - Advanced Search Database - CINAHL Complete | 11,621 |
| S131 | TI odontoma* OR AB odontoma* | Expanders - Apply equivalent subjects Search modes - Boolean/Phrase | Interface - EBSCOhost Research Databases Search Screen - Advanced Search Database - CINAHL Complete | 254 |
| S130 | TI NSCLC OR AB NSCLC | Expanders - Apply equivalent subjects Search modes - Boolean/Phrase | Interface - EBSCOhost Research Databases Search Screen - Advanced Search Database - CINAHL Complete | 14,310 |
| S129 | TI neurothekeom* OR AB neurothekeom* | Expanders - Apply equivalent subjects Search modes - Boolean/Phrase | Interface - EBSCOhost Research Databases Search Screen - Advanced Search Database - CINAHL Complete | 21 |
| S128 | TI neuroma* OR AB neuroma* | Expanders - Apply equivalent subjects Search modes - Boolean/Phrase | Interface - EBSCOhost Research Databases Search Screen - Advanced Search Database - CINAHL Complete | 1,534 |
| S127 | TI neuroblastom* OR AB neuroblastom* | Expanders - Apply equivalent subjects Search modes - Boolean/Phrase | Interface - EBSCOhost Research Databases Search Screen - Advanced Search Database - CINAHL Complete | 3,242 |
| S126 | TI neurilemmom* OR AB neurilemmom* | Expanders - Apply equivalent subjects Search modes - Boolean/Phrase | Interface - EBSCOhost Research Databases Search Screen - Advanced Search Database - CINAHL Complete | 77 |
| S125 | TI neurofibro* OR AB neurofibro* | Expanders - Apply equivalent subjects Search modes - Boolean/Phrase | Interface - EBSCOhost Research Databases Search Screen - Advanced Search Database - CINAHL Complete | 2,652 |
| S124 | TI neuroectodermal* OR AB neuroectodermal* | Expanders - Apply equivalent subjects Search modes - Boolean/Phrase | Interface - EBSCOhost Research Databases Search Screen - Advanced Search Database - CINAHL Complete | 649 |
| S123 | TI neurocytom* OR neurocytom* | Expanders - Apply equivalent subjects Search modes - Boolean/Phrase | Interface - EBSCOhost Research Databases Search Screen - Advanced Search Database - CINAHL Complete | 92 |
| S122 | TI neurilemmom* OR AB neurilemmom* | Expanders - Apply equivalent subjects Search modes - Boolean/Phrase | Interface - EBSCOhost Research Databases Search Screen - Advanced Search Database - CINAHL Complete | 77 |
| S121 | TI n#evocarcin* OR AB n#evocarcin* | Expanders - Apply equivalent subjects Search modes - Boolean/Phrase | Interface - EBSCOhost Research Databases Search Screen - Advanced Search Database - CINAHL Complete | 0 |
| S120 | TI myxo* OR AB myxo* | Expanders - Apply equivalent subjects Search modes - Boolean/Phrase | Interface - EBSCOhost Research Databases Search Screen - Advanced Search Database - CINAHL Complete | 2,269 |
| S119 | TI myosarcom* OR AB myosarcom* | Expanders - Apply equivalent subjects Search modes - Boolean/Phrase | Interface - EBSCOhost Research Databases Search Screen - Advanced Search Database - CINAHL Complete | 3 |
| S118 | TI myoma* OR AB myoma* | Expanders - Apply equivalent subjects Search modes - Boolean/Phrase | Interface - EBSCOhost Research Databases Search Screen - Advanced Search Database - CINAHL Complete | 1,074 |
| S117 | TI myeloma* OR AB myeloma* | Expanders - Apply equivalent subjects Search modes - Boolean/Phrase | Interface - EBSCOhost Research Databases Search Screen - Advanced Search Database - CINAHL Complete | 9,614 |
| S116 | TI myofibrom* OR AB myofibrom* | Expanders - Apply equivalent subjects Search modes - Boolean/Phrase | Interface - EBSCOhost Research Databases Search Screen - Advanced Search Database - CINAHL Complete | 131 |
| S115 | TI myoepitheliom* OR AB myoepitheliom* | Expanders - Apply equivalent subjects Search modes - Boolean/Phrase | Interface - EBSCOhost Research Databases Search Screen - Advanced Search Database - CINAHL Complete | 116 |
| S114 | TI myelolipom* OR AB myelolipom* | Expanders - Apply equivalent subjects Search modes - Boolean/Phrase | Interface - EBSCOhost Research Databases Search Screen - Advanced Search Database - CINAHL Complete | 145 |
| S113 | TI muir-torre* OR AB muir-torre* | Expanders - Apply equivalent subjects Search modes - Boolean/Phrase | Interface - EBSCOhost Research Databases Search Screen - Advanced Search Database - CINAHL Complete | 57 |
| S112 | TI micrometast* OR AB micrometast* | Expanders - Apply equivalent subjects Search modes - Boolean/Phrase | Interface - EBSCOhost Research Databases Search Screen - Advanced Search Database - CINAHL Complete | 824 |
| S111 | TI metaplas* OR AB metaplas* | Expanders - Apply equivalent subjects Search modes - Boolean/Phrase | Interface - EBSCOhost Research Databases Search Screen - Advanced Search Database - CINAHL Complete | 2,002 |
| S110 | TI mesotheliom* OR AB mesotheliom* | Expanders - Apply equivalent subjects Search modes - Boolean/Phrase | Interface - EBSCOhost Research Databases Search Screen - Advanced Search Database - CINAHL Complete | 3,041 |
| S109 | TI mesonephrom* OR AB mesonephrom* | Expanders - Apply equivalent subjects Search modes - Boolean/Phrase | Interface - EBSCOhost Research Databases Search Screen - Advanced Search Database - CINAHL Complete | 1 |
| S108 | TI mesoblast* OR AB mesoblast* | Expanders - Apply equivalent subjects Search modes - Boolean/Phrase | Interface - EBSCOhost Research Databases Search Screen - Advanced Search Database - CINAHL Complete | 72 |
| S107 | TI mesenchymom* OR AB mesenchymom* | Expanders - Apply equivalent subjects Search modes - Boolean/Phrase | Interface - EBSCOhost Research Databases Search Screen - Advanced Search Database - CINAHL Complete | 18 |
| S106 | TI meningiom* OR AB meningiom* | Expanders - Apply equivalent subjects Search modes - Boolean/Phrase | Interface - EBSCOhost Research Databases Search Screen - Advanced Search Database - CINAHL Complete | 2,036 |
| S105 | TI melano* OR AB melano* | Expanders - Apply equivalent subjects Search modes - Boolean/Phrase | Interface - EBSCOhost Research Databases Search Screen - Advanced Search Database - CINAHL Complete | 20,116 |
| S104 | TI meigs* OR AB meigs* | Expanders - Apply equivalent subjects Search modes - Boolean/Phrase | Interface - EBSCOhost Research Databases Search Screen - Advanced Search Database - CINAHL Complete | 85 |
| S103 | TI medulloblastom* OR AB medulloblastom* | Expanders - Apply equivalent subjects Search modes - Boolean/Phrase | Interface - EBSCOhost Research Databases Search Screen - Advanced Search Database - CINAHL Complete | 1,168 |
| S102 | TI mastocytO* OR AB mastocyto* | Expanders - Apply equivalent subjects Search modes - Boolean/Phrase | Interface - EBSCOhost Research Databases Search Screen - Advanced Search Database - CINAHL Complete | 559 |
| S101 | TI m#croprolactinom* OR AB m#croprolactinom* | Expanders - Apply equivalent subjects Search modes - Boolean/Phrase | Interface - EBSCOhost Research Databases Search Screen - Advanced Search Database - CINAHL Complete | 60 |
| S100 | TI macroglobulinem* OR AB macroglobulinem* | Expanders - Apply equivalent subjects Search modes - Boolean/Phrase | Interface - EBSCOhost Research Databases Search Screen - Advanced Search Database - CINAHL Complete | 474 |
| S99 | TI lynch* OR AB lynch* | Expanders - Apply equivalent subjects Search modes - Boolean/Phrase | Interface - EBSCOhost Research Databases Search Screen - Advanced Search Database - CINAHL Complete | 1,458 |
| S98 | TI lympho* OR AB lympho* | Expanders - Apply equivalent subjects Search modes - Boolean/Phrase | Interface - EBSCOhost Research Databases Search Screen - Advanced Search Database - CINAHL Complete | 68,806 |
| S97 | TI lymphangio* OR AB lymphangio* | Expanders - Apply equivalent subjects Search modes - Boolean/Phrase | Interface - EBSCOhost Research Databases Search Screen - Advanced Search Database - CINAHL Complete | 1,746 |
| S96 | TI luteoma* OR AB luteoma* | Expanders - Apply equivalent subjects Search modes - Boolean/Phrase | Interface - EBSCOhost Research Databases Search Screen - Advanced Search Database - CINAHL Complete | 24 |
| S95 | TI liposarcom* OR AB liposarcom* | Expanders - Apply equivalent subjects Search modes - Boolean/Phrase | Interface - EBSCOhost Research Databases Search Screen - Advanced Search Database - CINAHL Complete | 1,013 |
| S94 | TI lipoma* OR AB lipoma* | Expanders - Apply equivalent subjects Search modes - Boolean/Phrase | Interface - EBSCOhost Research Databases Search Screen - Advanced Search Database - CINAHL Complete | 2,065 |
| S93 | TI lipoblastom* OR AB lipoblastom* | Expanders - Apply equivalent subjects Search modes - Boolean/Phrase | Interface - EBSCOhost Research Databases Search Screen - Advanced Search Database - CINAHL Complete | 83 |
| S92 | TI li-fraumeni* OR AB li-fraumeni* | Expanders - Apply equivalent subjects Search modes - Boolean/Phrase | Interface - EBSCOhost Research Databases Search Screen - Advanced Search Database - CINAHL Complete | 247 |
| S91 | TI leukoplak* OR AB leukoplak* | Expanders - Apply equivalent subjects Search modes - Boolean/Phrase | Interface - EBSCOhost Research Databases Search Screen - Advanced Search Database - CINAHL Complete | 897 |
| S90 | TI preleukem* OR AB preleukem* | Expanders - Apply equivalent subjects Search modes - Boolean/Phrase | Interface - EBSCOhost Research Databases Search Screen - Advanced Search Database - CINAHL Complete | 56 |
| S89 | TI leukem* OR AB leukem* | Expanders - Apply equivalent subjects Search modes - Boolean/Phrase | Interface - EBSCOhost Research Databases Search Screen - Advanced Search Database - CINAHL Complete | 24,179 |
| S88 | TI leiomyosarcom* OR AB leiomyosarcom* | Expanders - Apply equivalent subjects Search modes - Boolean/Phrase | Interface - EBSCOhost Research Databases Search Screen - Advanced Search Database - CINAHL Complete | 1,359 |
| S87 | TI leiomyom* OR AB leiomyom* | Expanders - Apply equivalent subjects Search modes - Boolean/Phrase | Interface - EBSCOhost Research Databases Search Screen - Advanced Search Database - CINAHL Complete | 2,486 |
| S86 | TI leiomyoblastom* OR AB leiomyoblastom* | Expanders - Apply equivalent subjects Search modes - Boolean/Phrase | Interface - EBSCOhost Research Databases Search Screen - Advanced Search Database - CINAHL Complete | 4 |
| S85 | TI kasabach merrit* OR AB kasabach merrit* | Expanders - Apply equivalent subjects Search modes - Boolean/Phrase | Interface - EBSCOhost Research Databases Search Screen - Advanced Search Database - CINAHL Complete | 130 |
| S84 | TI insulinoma* OR AB insulinoma* | Expanders - Apply equivalent subjects Search modes - Boolean/Phrase | Interface - EBSCOhost Research Databases Search Screen - Advanced Search Database - CINAHL Complete | 666 |
| S83 | TI incidentaloma* OR AB incidentaloma* | Expanders - Apply equivalent subjects Search modes - Boolean/Phrase | Interface - EBSCOhost Research Databases Search Screen - Advanced Search Database - CINAHL Complete | 393 |
| S82 | TI immunocytom* OR AB immunocytom* | Expanders - Apply equivalent subjects Search modes - Boolean/Phrase | Interface - EBSCOhost Research Databases Search Screen - Advanced Search Database - CINAHL Complete | 6 |
| S81 | TI HNPCC OR AB HNPCC | Expanders - Apply equivalent subjects Search modes - Boolean/Phrase | Interface - EBSCOhost Research Databases Search Screen - Advanced Search Database - CINAHL Complete | 174 |
| S80 | TI hutchinson* OR AB hutchinson* | Expanders - Apply equivalent subjects Search modes - Boolean/Phrase | Interface - EBSCOhost Research Databases Search Screen - Advanced Search Database - CINAHL Complete | 372 |
| S79 | TI nonhodgkin* OR AB nonhodgkin* | Expanders - Apply equivalent subjects Search modes - Boolean/Phrase | Interface - EBSCOhost Research Databases Search Screen - Advanced Search Database - CINAHL Complete | 15 |
| S78 | TI hodgkin* OR AB hodgkin* | Expanders - Apply equivalent subjects Search modes - Boolean/Phrase | Interface - EBSCOhost Research Databases Search Screen - Advanced Search Database - CINAHL Complete | 7,643 |
| S77 | TI histiocytom* OR AB histiocytom* | Expanders - Apply equivalent subjects Search modes - Boolean/Phrase | Interface - EBSCOhost Research Databases Search Screen - Advanced Search Database - CINAHL Complete | 431 |
| S76 | TI hepatoma* OR AB hepatoma* | Expanders - Apply equivalent subjects Search modes - Boolean/Phrase | Interface - EBSCOhost Research Databases Search Screen - Advanced Search Database - CINAHL Complete | 936 |
| S75 | TI hepatoblastom* OR AB hepatoblastom* | Expanders - Apply equivalent subjects Search modes - Boolean/Phrase | Interface - EBSCOhost Research Databases Search Screen - Advanced Search Database - CINAHL Complete | 494 |
| S74 | TI hamartoblastom* OR AB hamartoblastom* | Expanders - Apply equivalent subjects Search modes - Boolean/Phrase | Interface - EBSCOhost Research Databases Search Screen - Advanced Search Database - CINAHL Complete | 2 |
| S73 | TI hemangiosarcom* OR AB hemangiosarcom* | Expanders - Apply equivalent subjects Search modes - Boolean/Phrase | Interface - EBSCOhost Research Databases Search Screen - Advanced Search Database - CINAHL Complete | 18 |
| S72 | TI hemangiopericytom* OR AB hemangiopericytom* | Expanders - Apply equivalent subjects Search modes - Boolean/Phrase | Interface - EBSCOhost Research Databases Search Screen - Advanced Search Database - CINAHL Complete | 266 |
| S71 | TI hemangiom* OR AB hemangiom* | Expanders - Apply equivalent subjects Search modes - Boolean/Phrase | Interface - EBSCOhost Research Databases Search Screen - Advanced Search Database - CINAHL Complete | 2,765 |
| S70 | TI hemangioendotheliom* OR AB hemangioendotheliom* | Expanders - Apply equivalent subjects Search modes - Boolean/Phrase | Interface - EBSCOhost Research Databases Search Screen - Advanced Search Database - CINAHL Complete | 449 |
| S69 | TI GIST OR AB GIST | Expanders - Apply equivalent subjects Search modes - Boolean/Phrase | Interface - EBSCOhost Research Databases Search Screen - Advanced Search Database - CINAHL Complete | 1,632 |
| S68 | TI GCTOB OR AB GCTOB | Expanders - Apply equivalent subjects Search modes - Boolean/Phrase | Interface - EBSCOhost Research Databases Search Screen - Advanced Search Database - CINAHL Complete | 0 |
| S67 | TI gonadoblastom* OR AB gonadoblastom* | Expanders - Apply equivalent subjects Search modes - Boolean/Phrase | Interface - EBSCOhost Research Databases Search Screen - Advanced Search Database - CINAHL Complete | 64 |
| S66 | TI glucagonoma* OR AB glucagonoma* | Expanders - Apply equivalent subjects Search modes - Boolean/Phrase | Interface - EBSCOhost Research Databases Search Screen - Advanced Search Database - CINAHL Complete | 64 |
| S65 | TI glomus tympanicum* OR AB glomus tympanicum* | Expanders - Apply equivalent subjects Search modes - Boolean/Phrase | Interface - EBSCOhost Research Databases Search Screen - Advanced Search Database - CINAHL Complete | 28 |
| S64 | TI glomus jugulare* OR AB glomus jugulare* | Expanders - Apply equivalent subjects Search modes - Boolean/Phrase | Interface - EBSCOhost Research Databases Search Screen - Advanced Search Database - CINAHL Complete | 60 |
| S63 | TI gliosarcom* OR AB gliosarcom* | Expanders - Apply equivalent subjects Search modes - Boolean/Phrase | Interface - EBSCOhost Research Databases Search Screen - Advanced Search Database - CINAHL Complete | 92 |
| S62 | TI glioma* OR AB glioma* | Expanders - Apply equivalent subjects Search modes - Boolean/Phrase | Interface - EBSCOhost Research Databases Search Screen - Advanced Search Database - CINAHL Complete | 6,348 |
| S61 | TI glioblastom* OR AB glioblastom* | Expanders - Apply equivalent subjects Search modes - Boolean/Phrase | Interface - EBSCOhost Research Databases Search Screen - Advanced Search Database - CINAHL Complete | 5,046 |
| S60 | TI germinoma* OR AB germinoma* | Expanders - Apply equivalent subjects Search modes - Boolean/Phrase | Interface - EBSCOhost Research Databases Search Screen - Advanced Search Database - CINAHL Complete | 216 |
| S59 | TI gastrinoma* OR AB gastrinoma* | Expanders - Apply equivalent subjects Search modes - Boolean/Phrase | Interface - EBSCOhost Research Databases Search Screen - Advanced Search Database - CINAHL Complete | 111 |
| S58 | TI ganglioneurom* OR AB ganglioneurom* | Expanders - Apply equivalent subjects Search modes - Boolean/Phrase | Interface - EBSCOhost Research Databases Search Screen - Advanced Search Database - CINAHL Complete | 219 |
| S57 | TI gangliogliom* OR AB gangliogliom* | Expanders - Apply equivalent subjects Search modes - Boolean/Phrase | Interface - EBSCOhost Research Databases Search Screen - Advanced Search Database - CINAHL Complete | 126 |
| S56 | TI FAMMM OR AB FAMMM | Expanders - Apply equivalent subjects Search modes - Boolean/Phrase | Interface - EBSCOhost Research Databases Search Screen - Advanced Search Database - CINAHL Complete | 4 |
| S55 | TI fibrosarcom* OR AB fibrosarcom* | Expanders - Apply equivalent subjects Search modes - Boolean/Phrase | Interface - EBSCOhost Research Databases Search Screen - Advanced Search Database - CINAHL Complete | 569 |
| S54 | TI fibroma* OR AB fibroma* | Expanders - Apply equivalent subjects Search modes - Boolean/Phrase | Interface - EBSCOhost Research Databases Search Screen - Advanced Search Database - CINAHL Complete | 1,670 |
| S53 | TI fibroepithelial* OR AB fibroepithelial* | Expanders - Apply equivalent subjects Search modes - Boolean/Phrase | Interface - EBSCOhost Research Databases Search Screen - Advanced Search Database - CINAHL Complete | 156 |
| S52 | TI fibroadenom* OR AB fibroadenom* | Expanders - Apply equivalent subjects Search modes - Boolean/Phrase | Interface - EBSCOhost Research Databases Search Screen - Advanced Search Database - CINAHL Complete | 426 |
| S51 | TI Ewing* OR AB Ewing* | Expanders - Apply equivalent subjects Search modes - Boolean/Phrase | Interface - EBSCOhost Research Databases Search Screen - Advanced Search Database - CINAHL Complete | 1,859 |
| S50 | TI ependymom* OR AB ependymom* | Expanders - Apply equivalent subjects Search modes - Boolean/Phrase | Interface - EBSCOhost Research Databases Search Screen - Advanced Search Database - CINAHL Complete | 735 |
| S49 | TI DSRCT OR AB DSRCT | Expanders - Apply equivalent subjects Search modes - Boolean/Phrase | Interface - EBSCOhost Research Databases Search Screen - Advanced Search Database - CINAHL Complete | 77 |
| S48 | TI DCIS OR AB DCIS | Expanders - Apply equivalent subjects Search modes - Boolean/Phrase | Interface - EBSCOhost Research Databases Search Screen - Advanced Search Database - CINAHL Complete | 1,437 |
| S47 | TI dysgerminoma* OR AB dysgerminoma* | Expanders - Apply equivalent subjects Search modes - Boolean/Phrase | Interface - EBSCOhost Research Databases Search Screen - Advanced Search Database - CINAHL Complete | 130 |
| S46 | TI desmoplas* OR AB desmoplas* | Expanders - Apply equivalent subjects Search modes - Boolean/Phrase | Interface - EBSCOhost Research Databases Search Screen - Advanced Search Database - CINAHL Complete | 687 |
| S45 | TI dermatofibrosarcom* OR AB dermatofibrosarcom* | Expanders - Apply equivalent subjects Search modes - Boolean/Phrase | Interface - EBSCOhost Research Databases Search Screen - Advanced Search Database - CINAHL Complete | 241 |
| S44 | TI cytoma* OR AB cytoma* | Expanders - Apply equivalent subjects Search modes - Boolean/Phrase | Interface - EBSCOhost Research Databases Search Screen - Advanced Search Database - CINAHL Complete | 7 |
| S43 | TI cystosarcom* OR AB cystosarcom* | Expanders - Apply equivalent subjects Search modes - Boolean/Phrase | Interface - EBSCOhost Research Databases Search Screen - Advanced Search Database - CINAHL Complete | 27 |
| S42 | TI cystadenofibrom* OR AB cystadenofibrom* | Expanders - Apply equivalent subjects Search modes - Boolean/Phrase | Interface - EBSCOhost Research Databases Search Screen - Advanced Search Database - CINAHL Complete | 42 |
| S41 | TI craniopharyngioma* OR AB craniopharyngioma* | Expanders - Apply equivalent subjects Search modes - Boolean/Phrase | Interface - EBSCOhost Research Databases Search Screen - Advanced Search Database - CINAHL Complete | 443 |
| S40 | TI choriocarcin* OR AB choriocarcin* | Expanders - Apply equivalent subjects Search modes - Boolean/Phrase | Interface - EBSCOhost Research Databases Search Screen - Advanced Search Database - CINAHL Complete | 578 |
| S39 | TI chondrosarcom* OR AB chondrosarcom* | Expanders - Apply equivalent subjects Search modes - Boolean/Phrase | Interface - EBSCOhost Research Databases Search Screen - Advanced Search Database - CINAHL Complete | 1,229 |
| S38 | TI chordoma* OR AB chordoma* | Expanders - Apply equivalent subjects Search modes - Boolean/Phrase | Interface - EBSCOhost Research Databases Search Screen - Advanced Search Database - CINAHL Complete | 609 |
| S37 | TI chondroma* OR AB chondroma* | Expanders - Apply equivalent subjects Search modes - Boolean/Phrase | Interface - EBSCOhost Research Databases Search Screen - Advanced Search Database - CINAHL Complete | 605 |
| S36 | TI chondroblastom* OR AB chondroblastom* | Expanders - Apply equivalent subjects Search modes - Boolean/Phrase | Interface - EBSCOhost Research Databases Search Screen - Advanced Search Database - CINAHL Complete | 167 |
| S35 | TI cholangiocarcin* OR AB cholangiocarcin* | Expanders - Apply equivalent subjects Search modes - Boolean/Phrase | Interface - EBSCOhost Research Databases Search Screen - Advanced Search Database - CINAHL Complete | 2,410 |
| S34 | TI chemodectomas* OR AB chemodectomas* | Expanders - Apply equivalent subjects Search modes - Boolean/Phrase | Interface - EBSCOhost Research Databases Search Screen - Advanced Search Database - CINAHL Complete | 6 |
| S33 | TI cementoma* OR AB cementoma* | Expanders - Apply equivalent subjects Search modes - Boolean/Phrase | Interface - EBSCOhost Research Databases Search Screen - Advanced Search Database - CINAHL Complete | 11 |
| S32 | TI carcin* OR carcin* | Expanders - Apply equivalent subjects Search modes - Boolean/Phrase | Interface - EBSCOhost Research Databases Search Screen - Advanced Search Database - CINAHL Complete | 132,888 |
| S31 | TI Buschke-Lowenstein* OR AB Buschke-Lowenstein* | Expanders - Apply equivalent subjects Search modes - Boolean/Phrase | Interface - EBSCOhost Research Databases Search Screen - Advanced Search Database - CINAHL Complete | 38 |
| S30 | TI Brenner* OR AB Brenner* | Expanders - Apply equivalent subjects Search modes - Boolean/Phrase | Interface - EBSCOhost Research Databases Search Screen - Advanced Search Database - CINAHL Complete | 186 |
| S29 | TI Bowen* OR AB Bowen* | Expanders - Apply equivalent subjects Search modes - Boolean/Phrase | Interface - EBSCOhost Research Databases Search Screen - Advanced Search Database - CINAHL Complete | 605 |
| S28 | TI blastom* OR AB blastom* | Expanders - Apply equivalent subjects Search modes - Boolean/Phrase | Interface - EBSCOhost Research Databases Search Screen - Advanced Search Database - CINAHL Complete | 598 |
| S27 | TI astrocytom* OR AB astrocytom* | Expanders - Apply equivalent subjects Search modes - Boolean/Phrase | Interface - EBSCOhost Research Databases Search Screen - Advanced Search Database - CINAHL Complete | 1,363 |
| S26 | TI arrhenoblastom* OR AB arrhenoblastom* | Expanders - Apply equivalent subjects Search modes - Boolean/Phrase | Interface - EBSCOhost Research Databases Search Screen - Advanced Search Database - CINAHL Complete | 3 |
| S25 | TI apudoma* OR AB apudoma* | Expanders - Apply equivalent subjects Search modes - Boolean/Phrase | Interface - EBSCOhost Research Databases Search Screen - Advanced Search Database - CINAHL Complete | 7 |
| S24 | TI angiosarcom* OR AB angiosarcom* | Expanders - Apply equivalent subjects Search modes - Boolean/Phrase | Interface - EBSCOhost Research Databases Search Screen - Advanced Search Database - CINAHL Complete | 985 |
| S23 | TI angiomyom* OR AB angiomyom* | Expanders - Apply equivalent subjects Search modes - Boolean/Phrase | Interface - EBSCOhost Research Databases Search Screen - Advanced Search Database - CINAHL Complete | 24 |
| S22 | TI angiomyolipom* OR AB angiomyolipom* | Expanders - Apply equivalent subjects Search modes - Boolean/Phrase | Interface - EBSCOhost Research Databases Search Screen - Advanced Search Database - CINAHL Complete | 696 |
| S21 | TI angioma* OR AB angioma* | Expanders - Apply equivalent subjects Search modes - Boolean/Phrase | Interface - EBSCOhost Research Databases Search Screen - Advanced Search Database - CINAHL Complete | 710 |
| S20 | TI angiolipom* OR AB angiolipom* | Expanders - Apply equivalent subjects Search modes - Boolean/Phrase | Interface - EBSCOhost Research Databases Search Screen - Advanced Search Database - CINAHL Complete | 79 |
| S19 | TI angiokeratom* OR AB angiokeratom* | Expanders - Apply equivalent subjects Search modes - Boolean/Phrase | Interface - EBSCOhost Research Databases Search Screen - Advanced Search Database - CINAHL Complete | 106 |
| S18 | TI angiofibrom* OR AB angiofibrom* | Expanders - Apply equivalent subjects Search modes - Boolean/Phrase | Interface - EBSCOhost Research Databases Search Screen - Advanced Search Database - CINAHL Complete | 341 |
| S17 | TI androblastom* OR AB androblastom* | Expanders - Apply equivalent subjects Search modes - Boolean/Phrase | Interface - EBSCOhost Research Databases Search Screen - Advanced Search Database - CINAHL Complete | 3 |
| S16 | TI ameloblastom* OR AB ameloblastom* | Expanders - Apply equivalent subjects Search modes - Boolean/Phrase | Interface - EBSCOhost Research Databases Search Screen - Advanced Search Database - CINAHL Complete | 713 |
| S15 | TI adeno* OR AB adeno* | Expanders - Apply equivalent subjects Search modes - Boolean/Phrase | Interface - EBSCOhost Research Databases Search Screen - Advanced Search Database - CINAHL Complete | 46,168 |
| S14 | TI adamantinom* OR AB adamantinom* | Expanders - Apply equivalent subjects Search modes - Boolean/Phrase | Interface - EBSCOhost Research Databases Search Screen - Advanced Search Database - CINAHL Complete | 76 |
| S13 | TI acrospiroma* OR AB acrospiroma* | Expanders - Apply equivalent subjects Search modes - Boolean/Phrase | Interface - EBSCOhost Research Databases Search Screen - Advanced Search Database - CINAHL Complete | 5 |
| S12 | TI acanthoma* OR AB acanthoma* | Expanders - Apply equivalent subjects Search modes - Boolean/Phrase | Interface - EBSCOhost Research Databases Search Screen - Advanced Search Database - CINAHL Complete | 49 |
| S11 | TI aberrant crypt foci OR AB aberrant crypt foci | Expanders - Apply equivalent subjects Search modes - Boolean/Phrase | Interface - EBSCOhost Research Databases Search Screen - Advanced Search Database - CINAHL Complete | 180 |
| S10 | TI malig* OR AB malig* | Expanders - Apply equivalent subjects Search modes - Boolean/Phrase | Interface - EBSCOhost Research Databases Search Screen - Advanced Search Database - CINAHL Complete | 83,269 |
| S9 | TI metast* OR AB metast* | Expanders - Apply equivalent subjects Search modes - Boolean/Phrase | Interface - EBSCOhost Research Databases Search Screen - Advanced Search Database - CINAHL Complete | 85,811 |
| S8 | TI onco* OR AB onco* | Expanders - Apply equivalent subjects Search modes - Boolean/Phrase | Interface - EBSCOhost Research Databases Search Screen - Advanced Search Database - CINAHL Complete | 81,489 |
| S7 | TI tumo#r* OR AB tumo#r* | Expanders - Apply equivalent subjects Search modes - Boolean/Phrase | Interface - EBSCOhost Research Databases Search Screen - Advanced Search Database - CINAHL Complete | 203,663 |
| S6 | TI * OR AB * | Expanders - Apply equivalent subjects Search modes - Boolean/Phrase | Interface - EBSCOhost Research Databases Search Screen - Advanced Search Database - CINAHL Complete | 451,887 |
| S5 | TI paraneoplas* OR AB paraneoplas* | Expanders - Apply equivalent subjects Search modes - Boolean/Phrase | Interface - EBSCOhost Research Databases Search Screen - Advanced Search Database - CINAHL Complete | 1,922 |
| S4 | TI neoplas* OR AB neoplas* | Expanders - Apply equivalent subjects Search modes - Boolean/Phrase | Interface - EBSCOhost Research Databases Search Screen - Advanced Search Database - CINAHL Complete | 30,040 |
| S3 | (MH " Survivors") | Expanders - Apply equivalent subjects Search modes - Boolean/Phrase | Interface - EBSCOhost Research Databases Search Screen - Advanced Search Database - CINAHL Complete | 12,107 |
| S2 | (MH "Cancer Patients") | Expanders - Apply equivalent subjects Search modes - Boolean/Phrase | Interface - EBSCOhost Research Databases Search Screen - Advanced Search Database - CINAHL Complete | 43,034 |
| S1 | (MH "Neoplasms+") | Expanders - Apply equivalent subjects Search modes - Boolean/Phrase | Interface - EBSCOhost Research Databases Search Screen - Advanced Search Database - CINAHL Complete | 616,934 |

**Scopus from Elsevier February 17, 2022**

( ( TITLE-ABS-KEY-AUTH ( neoplas* OR paraneoplas* OR * OR tumo?r* OR onco* OR metast* OR malignan* OR "aberrant crypt foci" OR acanthoma* OR acrospiroma* OR adamantinom* OR adenocarc* OR adenofibrom* OR adenolymphom* OR adenomat* OR adenomyo* OR adenosarcom* OR adenosquam* OR ameloblastom* OR androblastom* OR angiofibrom* OR angiokeratom* OR angiolipom* OR angioma* OR angiomyolipom* OR angiomyom* OR angiosarcom* OR apudoma* OR arrhenoblastom* OR astrocytom* OR blastom* OR bowen* OR brenner* OR "Buschke-Lowenstein" OR carcin* OR cementoma* OR chemodectomas* OR cholangiocarcin* OR chondroblastom* OR chondroma* OR chordoma* OR chondrosarcom* OR choriocarcin* OR craniopharyngioma* OR cystadenofibrom* OR cystosarcom* OR cytoma* OR dermatofibrosarcom* OR desmoplas* OR dysgerminoma* OR dcis OR dsrct OR ependymom* OR ewing* OR fibroadenom* OR fibroepithelial* OR fibroma* OR fibrosarcom* OR fammm OR gangliogliom* OR ganglioneurom* OR gastrinoma* OR germinoma* OR glioblastom* OR glioma* OR gliosarcom* OR "glomus jugulare" OR "glomus tympanicum" OR glucagonoma* OR gonadoblastom* OR gctob OR gist? OR hemangioendotheliom* OR hemangiom* OR hemangiopericytom* OR hemangiosarcom* OR hamartoblastom* OR hepatoblastom* OR hepatoma* OR histiocytom* OR hodgkin* OR nonhodgkin* OR "hutchinson freckle" OR hnpcc OR immunocytom* OR incidentaloma? OR insulinoma* OR "kasabach merit" OR leiomyoblastom* OR leiomyom* OR leiomyosarcom* OR leukem* OR preleukem* OR leukoplak* OR li-fraumeni* OR lipoblastom* OR lipoma* OR liposarcom* OR luteoma* OR lymphangio* OR lymphoblastom* OR lymphocytom* OR lymphoma* OR lymphosarcom* OR lynch* OR macroglobulinem* OR m?croprolactinom* OR mastocytom* OR mastocytos?s* OR medulloblastom* OR meigs* OR melanoameloblastom* OR melanoblastom* OR melanocarcin* OR melanoma* OR melanosis OR melanotic* OR meningiom* OR mesenchymom* OR mesoblast* OR mesonephrom* OR mesotheliom* OR metaplas* OR micrometast* OR "muir-torre" OR myelolipom* OR myoepitheliom* OR myofibrom* OR myeloma* OR myoma* OR myosarcom* OR myxofibrosarcom* OR myxoma* OR myxosarcom* OR n?evocarcin* OR neurilemmom* OR neurocytom* OR neuroectodermal* OR neurofibroma* OR neurofibrosarcom* OR neurilemmom* OR neuroblastom* OR neuroma* OR neurothekeom* OR nsclc OR odontoma* OR oligo* OR osteoblastom* OR osteochondrom* OR osteoclastom* OR osteoma* OR osteosarcom* OR papilloma* OR papillary* OR paragangliom* OR pheochromocytom* OR phyllo?des* OR pinealocytoma* OR pinealoma* OR pineoblastoma* OR pineocytoma* OR plasmacytom* OR "polycythem* vera" OR prolactinom* OR retinoblastom* OR rhabdoid* OR rhabdomyom* OR rhabdomyosarcom* OR sarcom* OR seminoma* OR "Sertoli- Leydig" OR somatostatinoma* OR somatotrophinom* OR "struma ovarii" OR thecoma* OR teratocarcin* OR teratoma* OR thymom* OR trophoblast* OR vipoma* OR wilms* ) ) AND ( TITLE-ABS-KEY-AUTH ( wearable? OR wear-able? OR acceleromet?r* OR actigraph* OR ( activit* W/3 mobile? ) OR ( activit* W/3 track* ) OR ( activit* W/3 monitor* ) OR ( fitness* W/3 mobile? ) OR ( fitness* W/3 band? ) OR ( fitness* W/3 device? ) OR ( fitness* W/3 track* ) OR ( fitness* W/3 monitor* ) OR ( move* W/3 mobile? ) OR movemonitor* OR move-monitor* OR ( physical* W/3 mobile? ) OR ( physical* W/3 track* ) OR ( ( selfmonitor* OR self-monitor* ) W/3 mobile? ) OR ( ( selfmonitor* OR self-monitor* ) W/3 device? ) OR selftrack* OR ( self W/3 track* ) OR ( ( wellness* OR well-ness* ) W/3 mobile? ) OR ( ( wellness* OR well-ness* ) W/3 device? ) OR ( ( wellness* OR well-ness* ) W/3 track* ) OR "mtrack" OR "m-track" OR "etrack" OR "e-track" OR ( ( biometric? OR bio-metric? ) W/3 mobile? ) OR ( ( biometric? OR bio-metric? ) W/3 monitor* ) OR ( ( biometric? OR bio-metric? ) W/3 sensor? ) OR ( remote* W/3 sensing ) OR ( embedded W/3 sensor? ) OR ( movement? W/3 sensor? ) OR ( ehealth* W/3 mobile? ) OR ( phealth* W/3 mobile? ) OR ( uhealth* W/3 mobile? ) OR ( smart* W/3 device? ) OR ( smart* W/3 technolog* ) OR ( smart* W/3 wireless* ) OR ( smart* W/3 sensor? ) OR ( smart* W/3 biosensor? ) OR ( smart* W/3 monitor* ) OR ( smart* W/3 track* ) OR ( smart* W/3 health* ) OR ( smart* W/3 ehealth* ) OR ( smart* W/3 mhealth* ) OR ( smart* W/3 phealth* ) OR ( smart* W/3 uhealth* ) OR ( smart* W/3 ( count OR counts OR counter? ) ) OR ( smart* W/3 logger? ) OR ( smart* W/3 wear* ) OR ( smart* W/3 worn* ) OR smartband? OR ( smart* W/3 band? ) OR ( smart* W/3 armband? ) OR smartglass* OR ( smart* W/3 glass* ) OR ( smart* W/3 ( eyewear? OR eye-wear? ) ) OR smartring? OR ( smart* W/3 ring? ) OR smartwatch* OR ( smart* W/3 watch* ) OR ( smart* W/3 cloth* ) OR ( smart* W/3 fabric? ) OR ( smart* W/3 apparel? ) OR ( smart* W/3 garment? ) OR ( electronic* W/3 textile? ) OR "e-textile?" OR etextile? OR ( smart* W/3 textile? ) OR ( smart* W/3 cap? ) OR ( smart* W/3 dress* ) OR ( smart* W/3 glove? ) OR ( smart* W/3 hat? ) OR ( smart* W/3 helmet? ) OR ( smart* W/3 jacket? ) OR ( smart* W/3 insoles* ) OR ( smart* W/3 pant? ) OR ( smart* W/3 scarf? ) OR ( smart* W/3 shirt? ) OR ( smart* W/3 footwear? ) OR ( smart* W/3 shoe? ) OR ( smart* W/3 sock? ) OR ( smart* W/3 strap? ) OR ( smart* W/3 suit? ) OR ( smart* W/3 vest? ) OR ( smart* W/3 societ* ) OR ( intelligent W/3 sensor? ) OR ( intelligen* W/3 biosensor? ) OR ( intelligen* W/3 wear* ) OR ( intelligen* W/3 worn* ) OR ( intelligen* W/3 band? ) OR ( intelligen* W/3 armband? ) OR ( intelligen* W/3 glass* ) OR ( intelligen* W/3 ( eyewear? OR eye-wear? ) ) OR ( intelligen* W/3 ring? ) OR ( intelligen* W/3 watch* ) OR ( intelligen* W/3 cloth* ) OR ( intelligen* W/3 fabric? ) OR ( intelligen* W/3 apparel? ) OR ( intelligen* W/3 garment? ) OR ( intelligen* W/3 textile? ) OR ( intelligen* W/3 belt? ) OR ( intelligen* W/3 cap? ) OR ( intelligen* W/3 dress* ) OR ( intelligen* W/3 glove? ) OR ( intelligen* W/3 hat? ) OR ( intelligen* W/3 helmet? ) OR ( intelligen* W/3 jacket? ) OR ( intelligen* W/3 insoles* ) OR ( intelligen* W/3 pant? ) OR ( intelligen* W/3 scarf? ) OR ( intelligen* W/3 shirt? ) OR ( intelligen* W/3 footwear? ) OR ( intelligen* W/3 shoe? ) OR ( intelligen* W/3 sock? ) OR ( intelligen* W/3 strap? ) OR ( intelligen* W/3 suit? ) OR ( intelligen* W/3 vest? ) OR "I-wear" OR ( ambient* W/3 intelligen* ) OR ( ambient* W/3 wear* ) OR ( ambient* W/3 worn* ) OR sensor?-wear* OR sens*wear* OR ( wireless* W/3 wear* ) OR ( wireless* W/3 worn* ) OR ( wireless* W/3 biosensor* ) OR ( wireless* W/3 textile? ) OR ( wireless W/3 body W/3 area W/3 network? ) OR ( wireless W/3 sensor? W/3 network-based ) OR wban? OR "ankle-worn" OR "ankle-mount" OR "finger-worn" OR "waist-worn" OR "waist-mount" OR "wrist-worn" OR "wrist-mount" OR pedometer? OR step?count* OR ( step? W/3 count* ) OR ( adidas W/3 watch* ) OR ( apple W/3 watch* ) OR ( boat W/3 watch* ) OR ( casio W/3 watch* ) OR amazfit? OR fitbit? OR ( fossil W/3 watch* ) OR garmin? OR ( hexoskin W/3 watch* ) OR ( huawei W/3 watch* ) OR ( jawbone W/3 watch* ) OR ( lg W/3 watch* ) OR misfit? OR ( mi W/3 watch* ) OR ( nike W/3 watch* ) OR ( puma W/3 watch* ) OR ( samsung W/3 watch* ) OR ( xiaomi W/3 watch* ) OR whoop OR ( consumer? W/5 ( base? OR level? OR grade? ) W/5 device? ) OR ( consumer? W/5 ( base? OR level? OR grade? ) W/5 measur* ) OR ( consumer? W/5 ( base? OR level? OR grade? ) W/5 monitor* ) OR ( consumer? W/5 ( base? OR level? OR grade? ) W/5 tracker? ) OR ( consumer? W/5 activit* W/5 device? ) OR ( consumer? W/5 activit* W/5 measur* ) OR ( consumer? W/5 activit* W/5 monitor* ) OR ( consumer? W/5 activit* W/5 tracker? ) OR ( consumer? W/5 pam? ) OR ( consumer? W/5 imu? ) ) ) ) AND ( TITLE-ABS-KEY-AUTH ( random* OR nonrandom* OR quasirandom* OR ( ( quasiexperimental OR quasi-experimental ) W/4 ( study OR studies ) ) OR ( control* W/4 ( study OR studies OR group* ) ) OR ( allocate* W/4 ( study OR studies OR group* ) ) OR ( pragmatic* W/4 ( study OR studies ) ) OR ( equivalence W/4 ( study OR studies ) ) OR ( superiority W/4 ( study OR studies ) ) OR ( ( noninferiority OR non-inferiority ) W/4 ( study OR studies ) ) OR ( pivotal* W/4 ( study OR studies ) ) OR ( comparative W/4 ( study OR studies ) ) OR ( comparative W/2 effective* ) OR openlabel* OR open-label* OR closedlabel* OR closed-label* OR ( conceal* W/2 allocat* ) OR ( intention* W/2 treat* W/2 analys?s* ) OR feasibility OR trial? OR phase? OR multicent?r* OR multi-cent?r* OR "single blind*" OR "single mask*" OR "single dumm*" OR "double blind*" OR "double mask*" OR "double dumm*" OR "triple-blind*" OR "triple mask*" OR "triple dumm*" OR "treble blind*" OR "treble mask*" OR "treble dumm*" OR placebo* ) ) AND ( LIMIT-TO ( DOCTYPE , "ar" ) OR LIMIT-TO ( DOCTYPE , "re" ) )

**2615 document results**

**Web of Science Core Collection (Sci-expanded, SSCI, AHCI, ESCI) from Clarivate Analytics February 17, 2022**

**#1 5585377**

TS=(neoplas* OR paraneoplas* OR * OR tumo?r* OR onco* OR metast* OR malignan* OR aberrant crypt foci OR acanthoma* OR acrospiroma* OR adamantinom* OR adenocarc* OR adenofibrom* OR adenolymphom* OR adenomat* OR adenomyo* OR adenosarcom* OR adenosquam* OR ameloblastom* OR androblastom* OR angiofibrom* OR angiokeratom* OR angiolipom* OR angioma* OR angiomyolipom* OR angiomyom* OR angiosarcom* OR apudoma* OR arrhenoblastom* OR astrocytom* OR blastom* OR Bowen* OR Brenner* OR Buschke-Lowenstein* OR carcin* OR cementoma* OR chemodectomas* OR cholangiocarcin* OR chondroblastom* OR chondroma* OR chordoma* OR chondrosarcom* OR choriocarcin* OR craniopharyngioma* OR cystadenofibrom* OR cystosarcom* OR cytoma* OR dermatofibrosarcom* OR desmoplas* OR dysgerminoma* OR DCIS OR DSRCT OR ependymom* OR Ewing* OR fibroadenom* OR fibroepithelial* OR fibroma* OR fibrosarcom* OR FAMMM OR gangliogliom* OR ganglioneurom* OR gastrinoma* OR germinoma* OR glioblastom* OR glioma* OR gliosarcom* OR glomus jugulare* OR glomus tympanicum* OR glucagonoma* OR gonadoblastom* OR GCTOB OR GIST? OR hemangioendotheliom* OR hemangiom* OR hemangiopericytom* OR hemangiosarcom* OR hamartoblastom* OR hepatoblastom* OR hepatoma* OR histiocytom* OR hodgkin* OR nonhodgkin* OR hutchinson* freckle* OR HNPCC OR immunocytom* OR incidentaloma? OR insulinoma* OR kasabach merrit* OR leiomyoblastom* OR leiomyom* OR leiomyosarcom* OR leukem* OR preleukem* OR leukoplak* OR li-fraumeni* OR lipoblastom* OR lipoma* OR liposarcom* OR luteoma* OR lymphangio* OR lymphoblastom* OR lymphocytom* OR lymphoma* OR lymphosarcom* OR lynch* OR macroglobulinem* OR m?croprolactinom* OR mastocytom* OR mastocytos?s* OR medulloblastom* OR meigs* OR melanoameloblastom* OR melanoblastom* OR melanocarcin* OR melanoma* OR melanosis OR melanotic* OR meningiom* OR mesenchymom* OR mesoblast* OR mesonephrom* OR mesotheliom* OR metaplas* OR micrometast* OR muir-torre* OR myelolipom* OR myoepitheliom* OR myofibrom* OR myeloma* OR myoma* OR myosarcom* OR myxofibrosarcom* OR myxoma* OR myxosarcom* OR n?evocarcin* OR neurilemmom* OR neurocytom* OR neuroectodermal* OR neurofibroma* OR neurofibrosarcom* OR neurilemmom* OR neuroblastom* OR neuroma* OR neurothekeom* OR NSCLC OR odontoma* OR oligo* OR osteoblastom* OR osteochondrom* OR osteoclastom* OR osteoma* OR osteosarcom* OR papilloma* OR papillary* OR paragangliom* OR pheochromocytom* OR phyllo?des* OR pinealocytoma* OR pinealoma* OR pineoblastoma* OR pineocytoma* OR plasmacytom* OR polycythem* vera? OR prolactinom* OR retinoblastom* OR rhabdoid* OR rhabdomyom* OR rhabdomyosarcom* OR sarcom* OR seminoma* OR Sertoli- Leydig OR somatostatinoma* OR somatotrophinom* OR struma ovarii* OR thecoma* OR teratocarcin* OR teratoma* OR thymom* OR trophoblast* OR vipoma* OR wilms*)

**#2 239341**

TS=(wearable? OR wear-able? OR acceleromet?r* OR actigraph* OR (activit* NEAR/3 mobile?) OR (activit* NEAR/3 track*) OR (activit* NEAR/3 monitor*) OR (fitness* NEAR/3 mobile?) OR (fitness* NEAR/3 band?) OR (fitness* NEAR/3 device?) OR (fitness* NEAR/3 track*) OR (fitness* NEAR/3 monitor*) OR (move* NEAR/3 mobile?) OR movemonitor* OR move-monitor* OR (physical* NEAR/3 mobile?) OR (physical* NEAR/3 track*) OR ((selfmonitor* or self-monitor*) NEAR/3 mobile?) OR ((selfmonitor* or self-monitor*) NEAR/3 device?) OR selftrack* OR (self NEAR/3 track*) OR ((wellness* or well-ness*) NEAR/3 mobile?) OR ((wellness* or well-ness*) NEAR/3 device?) OR ((wellness* or well-ness*) NEAR/3 track*) OR mtrack* OR m-track* OR etrack* OR e-track* OR ((biometric? or bio-metric?) NEAR/3 mobile?) OR ((biometric? or bio-metric?) NEAR/3 monitor*) OR ((biometric? or bio-metric?) NEAR/3 sensor?) OR (remote* NEAR/3 sensing) OR (embedded NEAR/3 sensor?) OR (movement? NEAR/3 sensor?) OR (ehealth* NEAR/3 mobile?) OR (phealth* NEAR/3 mobile?) OR (uhealth* NEAR/3 mobile?) OR (smart* NEAR/3 device?) OR (smart* NEAR/3 technolog*) OR (smart* NEAR/3 wireless*) OR (smart* NEAR/3 sensor?) OR (smart* NEAR/3 biosensor?) OR (smart* NEAR/3 monitor*) OR (smart* NEAR/3 track*) OR (smart* NEAR/3 health*) OR (smart* NEAR/3 ehealth*) OR (smart* NEAR/3 mhealth*) OR (smart* NEAR/3 phealth*) OR (smart* NEAR/3 uhealth*) OR (smart* NEAR/3 (count or counts or counter?)) OR (smart* NEAR/3 logger?) OR (smart* NEAR/3 wear*) OR (smart* NEAR/3 worn*) OR smartband? OR (smart* NEAR/3 band?) OR (smart* NEAR/3 armband?) OR smartglass* OR (smart* NEAR/3 glass*) OR (smart* NEAR/3 (eyewear? or eye-wear?)) OR smartring? OR (smart* NEAR/3 ring?) OR smartwatch* OR (smart* NEAR/3 watch*) OR (smart* NEAR/3 cloth*) OR (smart* NEAR/3 fabric?) OR (smart* NEAR/3 apparel?) OR (smart* NEAR/3 garment?) OR (electronic* NEAR/3 textile?) OR (e-textile? or etextile?) OR (smart* NEAR/3 textile?) OR (smart* NEAR/3 cap?) OR (smart* NEAR/3 dress*) OR (smart* NEAR/3 glove?) OR (smart* NEAR/3 hat?) OR (smart* NEAR/3 helmet?) OR (smart* NEAR/3 jacket?) OR (smart* NEAR/3 insoles*) OR (smart* NEAR/3 pant?) OR (smart* NEAR/3 scarf?) OR (smart* NEAR/3 shirt?) OR (smart* NEAR/3 footwear?) OR (smart* NEAR/3 shoe?) OR (smart* NEAR/3 sock?) OR (smart* NEAR/3 strap?) OR (smart* NEAR/3 suit?) OR (smart* NEAR/3 vest?) OR (smart* NEAR/3 societ*) OR (intelligent NEAR/3 sensor?) OR (intelligen* NEAR/3 biosensor?) OR (intelligen* NEAR/3 wear*) OR (intelligen* NEAR/3 worn*) OR (intelligen* NEAR/3 band?) OR (intelligen* NEAR/3 armband?) OR (intelligen* NEAR/3 glass*) OR (intelligen* NEAR/3 (eyewear? or eye-wear?)) OR (intelligen* NEAR/3 ring?) OR (intelligen* NEAR/3 watch*) OR (intelligen* NEAR/3 cloth*) OR (intelligen* NEAR/3 fabric?) OR (intelligen* NEAR/3 apparel?) OR (intelligen* NEAR/3 garment?) OR (intelligen* NEAR/3 textile?) OR (intelligen* NEAR/3 belt?) OR (intelligen* NEAR/3 cap?) OR (intelligen* NEAR/3 dress*) OR (intelligen* NEAR/3 glove?) OR (intelligen* NEAR/3 hat?) OR (intelligen* NEAR/3 helmet?) OR (intelligen* NEAR/3 jacket?) OR (intelligen* NEAR/3 insoles*) OR (intelligen* NEAR/3 pant?) OR (intelligen* NEAR/3 scarf?) OR (intelligen* NEAR/3 shirt?) OR (intelligen* NEAR/3 footwear?) OR (intelligen* NEAR/3 shoe?) OR (intelligen* NEAR/3 sock?) OR (intelligen* NEAR/3 strap?) OR (intelligen* NEAR/3 suit?) OR (intelligen* NEAR/3 vest?) OR I-wear* OR (ambient* NEAR/3 intelligen*) OR (ambient* NEAR/3 wear*) OR (ambient* NEAR/3 worn*) OR sensor?-wear* OR sens*wear* OR (wireless* NEAR/3 wear*) OR (wireless* NEAR/3 worn*) OR (wireless* NEAR/3 biosensor*) OR (wireless* NEAR/3 textile?) OR (wireless body NEAR/3 area network?) OR (wireless sensor? NEAR/3 network-based) OR WBAN? OR ankle?-worn* OR ankle?-mount* OR finger?-worn* OR waist?-worn* OR waist?-mount* OR wrist?-worn* OR wrist?-mount* OR pedometer? OR step?count* OR (step? NEAR/3 count*) OR (adidas NEAR/3 watch*) OR (apple NEAR/3 watch*) OR (boat NEAR/3 watch*) OR (casio NEAR/3 watch*) OR amazfit? OR fitbit? OR (fossil NEAR/3 watch*) OR garmin? OR (hexoskin NEAR/3 watch*) OR (huawei NEAR/3 watch*) OR (jawbone NEAR/3 watch*) OR (lg NEAR/3 watch*) OR misfit? OR (mi NEAR/3 watch*) OR (nike NEAR/3 watch*) OR (puma NEAR/3 watch*) OR (samsung NEAR/3 watch*) OR (xiaomi NEAR/3 watch*) OR whoop OR (consumer? NEAR/5 (base? or level? or grade?) NEAR/5 device?) OR (consumer? NEAR/5 (base? or level? or grade?) NEAR/5 measur*) OR (consumer? NEAR/5 (base? or level? or grade?) NEAR/5 monitor*) OR (consumer? NEAR/5 (base? or level? or grade?) NEAR/5 tracker?) OR (consumer? NEAR/5 activit* NEAR/5 device?) OR (consumer? NEAR/5 activit* NEAR/5 measur*) OR (consumer? NEAR/5 activit* NEAR/5 monitor*) OR (consumer? NEAR/5 activit* NEAR/5 tracker?) OR (consumer? NEAR/5 PAM?) OR (consumer? NEAR/5 IMU?))

**#3 5521**

#1 AND #2

**#4 3856605**

TS=(random* OR nonrandom* OR quasirandom* OR quasiexperimental OR quasi-experimental OR closed label OR closed-label OR open label OR open-label OR feasibility OR trial? OR phase? OR multicent?r* OR multi-cent?r* OR "single blind*" OR "single mask*" OR "single dumm*" OR "double blind*" OR "double mask*" OR "double dumm*" OR "triple-blind*" OR "triple mask*" OR "triple dumm*" OR "treble blind*" OR "treble mask*" OR "treble dumm*" OR sham OR placebo*)

**#5 1320414**

TS= ((control* NEAR/4 (study or studies or group*)) OR (allocate* NEAR/4 (study or studies or group*)) OR (pragmatic* NEAR/4 (study or studies)) OR (equivalence NEAR/4 (study or studies)) OR (superiority NEAR/4 (study or studies)) OR ((noninferiority or non-inferiority) NEAR/4 (study or studies)) OR (pivotal* NEAR/4 (study or studies)) OR (comparative NEAR/4 (study or studies)) OR (comparative NEAR/2 effective*) OR (conceal* NEAR/2 allocat*) OR (intention* NEAR/2 treat* NEAR/2 analys?s*))

**#6 4768893**

#4 OR #5

**#7** **1146**

#3 AND #6

**#8 1098**

#7 and Proceedings Papers or Meeting Abstracts or Book Chapters (Exclude – Document Types)
